# Development and Characterization of a Noncovalent Stimulator of Interferon Genes Proteolysis‐Targeting Chimeras

**DOI:** 10.1002/cmdc.202500715

**Published:** 2025-11-18

**Authors:** Bo Hu, Adam S. Duerfeldt

**Affiliations:** ^1^ Department of Medicinal Chemistry College of Pharmacy University of Minnesota Minneapolis MN 55455 USA

**Keywords:** cyclic GMP‐AMP synthase, inflammation, proteolysis targeting chimeras, protein degradation, stimulator of interferon genes, structure–activity relationships

## Abstract

The cyclic GMP‐AMP synthase (cGAS)‐stimulator of interferon genes (STING) pathway activates the immune response upon detection of cytosolic dsDNA and is a key regulator of innate immunity. Overactivation of cGAS‐STING has been implicated in numerous inflammatory diseases, and inhibition of cGAS‐STING signaling has attracted significant interest as a therapeutic approach to attenuate aberrant inflammation. Proteolysis‐targeting chimeras (PROTACs) have become popular modalities for catalyzing the degradation of proteins of interest, thus inhibiting their function. Herein, the design, synthesis, and characterization of noncovalent catalytic STING PROTACs based on a known diphenyl‐dihydroisoquinolone STING inhibitory chemotype are reported. The lead from this series (BH690L) exhibits an effective concentration for half‐maximal degradation (DC_50_) of 11.3 nM and a maximum level of degradation observed for a given concentration of PROTAC (D_max_) of 0.67, and elicits suppression of downstream markers of inflammation.

## Graphical abstract

The design, synthesis, and initial biological evaluation of noncovalent stimulator of interferon genes degraders are presented. The lead from this series, BH690L, exhibits an effective concentration for half‐maximal degradation (DC_50_) of 11.3 nM and a maximum level of degradation (D_max_) of 0.67.

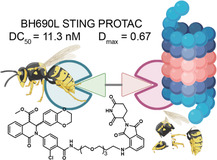

## 1 Introduction

The presence of cytoplasmic double‐stranded DNA (dsDNA) is abnormal and caused by leakage of nucleic acid from the mitochondria or nucleus, localized cell death, or infection.^[^ ^1^ ^]^ Upon binding dsDNA, cyclic GMP‐AMP synthase (cGAS) catalyzes the synthesis of cyclic guanosine monophosphate‐adenosine monophosphate (cGAMP) from ATP and GTP, which in turn activates the stimulator of interferon genes (STING).^[^ ^1^ ^]^ This activation triggers a signaling cascade that leads to the phosphorylation and activation of interferon regulatory factor 3 (IRF3), ultimately triggering the production of interferons and initiating an immune response.^[^ ^1^ ^]^ Overactivation of the cGAS‐STING pathway has been implicated in a wide range of human diseases, especially in the context of autoimmune conditions.^[^ ^2^ ^]^ Consequently, inhibition of cGAS‐STING signaling has generated considerable interest for immunomodulation, with several inhibitory chemotypes having been reported.^[^ ^2^ ^]^

Systemic inhibition of the cGAS‐STING pathway has been met with significant concerns, particularly the risk of immunocompromise due to the pathway's central role in innate immunity.^[^ ^3^ ^,^ ^4^ ^]^ Recently, however, aberrant STING activation has been implicated in retinal diseases, inspiring our group and others to pursue STING inhibitors as therapeutic leads for diabetic retinopathy and other common inflammatory diseases of the retina.^[^ ^5^ ^]^ For these conditions, localized drug administration is the standard of care (e.g., intravitreal injections), providing the opportunity for drug sequestration, especially due to low blood‐retinal barrier permeability.^[^ ^6^ ^]^ As such, the eye is positioned as an optimal organ to target the cGAS‐STING pathway for disease mitigation without the perceived risks associated with systemic modulation of this pathway.^[^ ^6^ ^]^ Small molecules, however, are not typically amenable to intravitreal injection, due to rapid intraocular clearance, limiting efficacy.^[^ ^6^ ^]^ In contrast, larger STING inhibitors >500 g mol^−1^ would be expected to be sequestered in the eye, attenuating the inflammatory pathogenesis, and eliminating the risks associated with broader immune suppression.^[^ ^6^ ^]^

Proteolysis‐targeting chimeras (PROTACs) are large bifunctional molecules that induce proximity between a protein of interest (POI) and a ubiquitin E3 ligase, leading to the polyubiquitination and proteolysis of the target protein.^[^ ^7^ ^]^ PROTACs consist of a POI ligand conjugated to an E3 ligase ligand via a linker.^[^ ^7^ ^]^ Development of PROTACs as therapeutics has drawn considerable interest due to their catalytic nature and the attractiveness of reviving and repurposing previously discovered ligands as PROTACs.^[^ ^7^ ^]^ Given the larger molecular size of PROTACs, these modalities would be well‐suited for local administration into the eye and ocular sequestration.^[^ ^6^ ^]^ To date, the PROTAC approach has been rarely applied to ocular targets, and we hypothesize that targeting retinal STING with this approach would provide proof of concept for this strategy.^[^ ^8^ ^,^ ^9^ ^]^ To enable the testing of this hypothesis, the first step is the development of a STING PROTAC. Although SP23 has been reported as a STING degrader, the covalent nature of this molecule abrogates the catalytic activity of the PROTAC, eliminating a key benefit of PROTAC approaches.^[^ ^10^ ^]^ We chose to pursue the development of a non‐covalent PROTAC chemotype that would retain the ability to operate catalytically.

## 2 Results and Discussion

**Figure 1:**
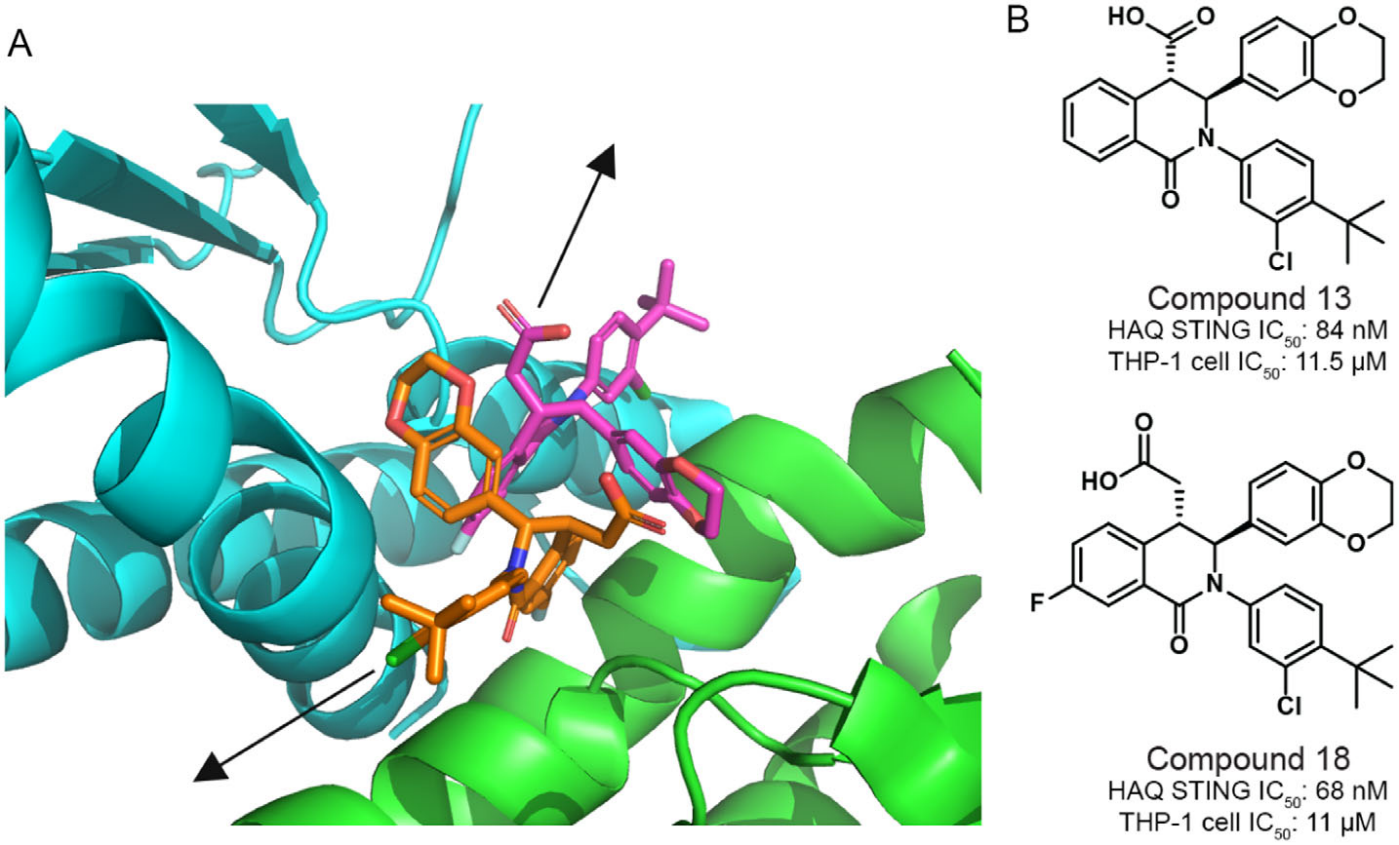
A) Cocrystal structure of compound 18 with human STING (PDBID: 6MXE). Arrows indicate the two putative vectors identified for linker attachment. B) Structures and reported biological activity of compound 13 and compound 18. HAQ STING (a common STING allele with ≈20% frequency, present in THP‐1 cells) in a cell‐free model shows double‐digit nanomolar IC_50_, while poor membrane penetration in intact THP‐1 cells raises IC_50_ of both compounds to ≈11 μM. Compared to compound 13, compound 18 features a higher pKa (4.3 versus 3.4), higher bioavailability (5% versus 60%), and higher membrane penetration (18 × 10^−6^ cm s^−^^1^ versus 9 × 10^−6^ cm s^−^^1^).^[^ ^11^ ^]^

A recent report identified a series of dihydroisoquinolone analogs as small molecule STING inhibitors.^[^ ^11^ ^]^ From the reported series, we selected compound 13 (**Figure** **1**B) as the parent POI ligand to develop a series of PROTACs, due to its synthetic tractability. This compound exhibited an IC_50_ of 84 nM in a cell‐free cGAMP displacement assay, and a STING IC_50_ of 11.5 μM in THP‐1 cells.^[^ ^11^ ^]^ While a ligand with a cell‐based IC_50_ of 11.5 μM is not typically considered a reasonable starting point for PROTAC development, we hypothesized that cell permeation was the key issue and with longer incubations, the cellular IC_50_ would approach the cell‐free value of 84 nM.

We utilized the available cocrystal structure of a highly similar compound 18 to determine possible vectors for linker attachment.^[^ ^11^ ^]^ Of note is that compounds 13 and 18 feature a 2:1 binding stoichiometry with the STING dimer, with a considerable binding interface between the two ligands.^[^ ^11^ ^]^ This limited possible vectors and presented a challenge to consider during PROTAC design and structure–activity relationship interpretation. As shown in Figure 1A, two potential vectors for linker attachment were identified 1) projection from the carboxylic acid location that points toward the unstructured “lid” of STING and 2) projection from the *t*‐butyl position that points along the dimerization interface. Neither vector had previously been explored for extension compatibility. Either vector presented potential liabilities to PROTAC development. For example, the “lid” is disordered in the STING apo state and the presence of unmodeled amino acids could interfere with linker attachment at the carboxylate position. Likewise, modification of the *t*‐butyl group could affect STING dimerization, undermining ligand binding. As such, we synthesized compounds to explore both vectors with several linker lengths and types. To bind E3 ligase, a cereblon (CRBN)‐binding ligand was chosen due to widespread CRBN expression, including in the retina (Figure S1, Supporting Information), and the known ability for CRBN recruitment to degrade STING.^[^ ^10^ ^]^ Because the synthetic route is modular, bifunctional compounds containing a von Hippel–Lindau (VHL)‐binding ligand were also synthesized. As E3 ligase selection can be imperative to PROTAC design, this approach allowed us to assess the potential of the two most traditionally targeted E3 ligases.

**Scheme 1:**
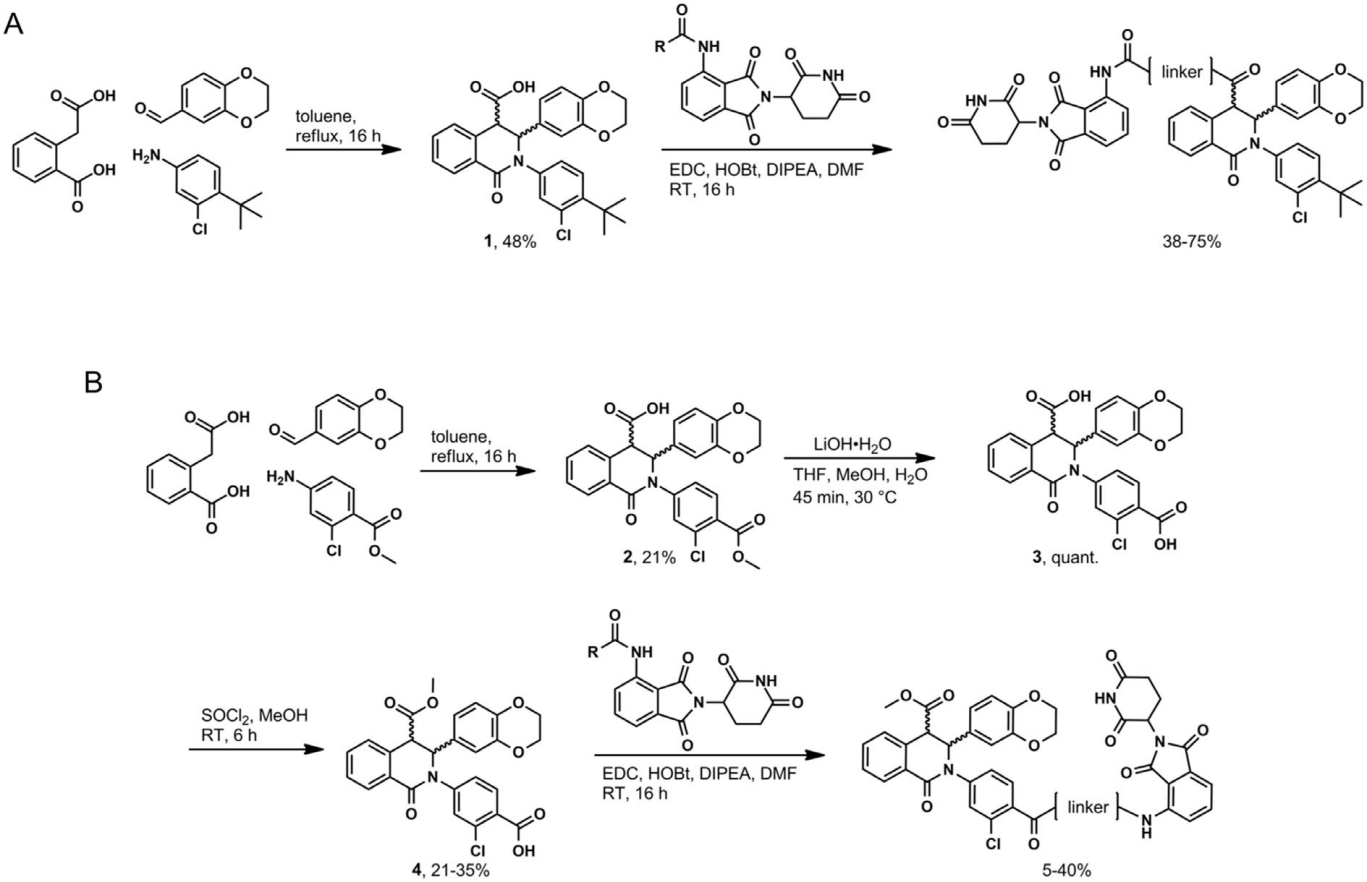
Synthesis of CRBN‐recruiting PROTACs. A) Synthesis of compounds with linker attachment replacing the carboxylic acid. B) Synthesis of compounds with linker attachment replacing the *t*‐butyl group. All yields reported are for the racemic mixture. VHL‐recruiting compounds are synthesized using similar methods and their synthetic routes are found in the supplemental information.

**Figure 2:**
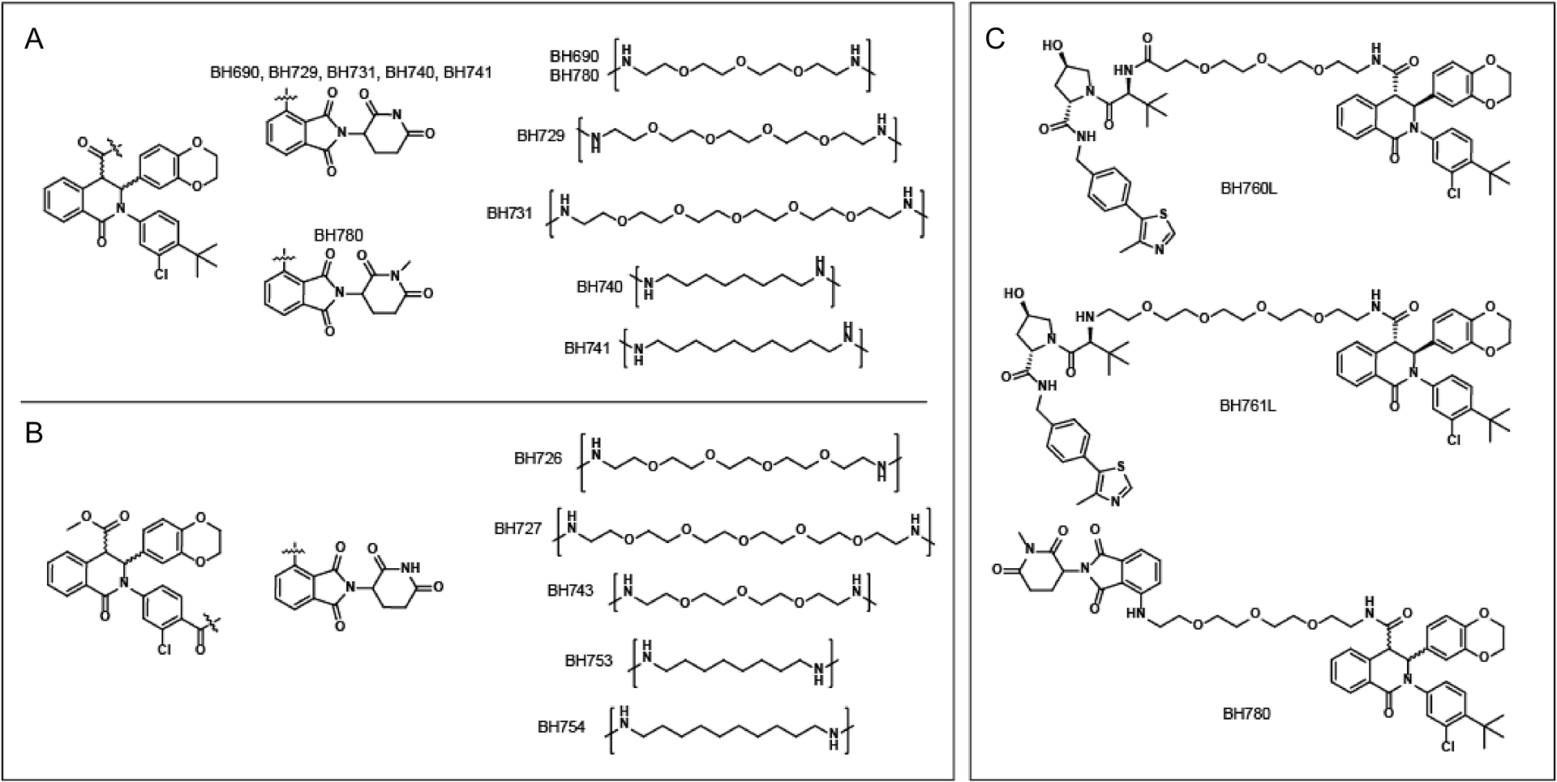
Synthesized bifunctional compounds. A) CRBN‐recruiting compounds linked from the carboxylic acid vector. B) CRBN‐recruiting compounds linked from the *t*‐butyl vector. C) Two VHL‐recruiting compounds (760 and 761L) and a non‐E3‐recruiting control compound (780).

Fragment **1** of compound 13 was synthesized via a three‐component Castagnoli‐Cushman reaction, yielding a 1:1 mixture of *trans* enantiomers.^[^ ^11^ ^]^ For PROTACs with linker attachment through the carboxylic acid vector (**Scheme** **1**A), amide coupling to a preformed thalidomide‐linker conjugate provided the final compounds as racemic mixtures. For scaffolds requiring replacement of the *t*‐butyl group with a carboxylic acid, the Castagnoli‐Cushman reaction was again employed (**2**, Scheme 1B). Saponification to provide the dicarboxylic acids **3**, followed by regioselective esterification to yield intermediates **4**, and subsequent amide coupling, provided the products as mixtures of enantiomers. VHL‐recruiting PROTACs were synthesized using similar chemistry but employed the VHL‐conjugated linkers (Scheme S1, Supporting Information). Racemic mixtures of final compounds could be separated by chiral HPLC as demonstrated for BH690, BH741, BH760, BH761, and BH780 (**Figure** **2** and supplementary data). For example, the first BH690 enantiomer to elute from a Phenomenex Lux Cellulose‐1 chiral column was levorotatory and designated L (e.g., BH690L); the second enantiomer was dextrorotatory and designated R (e.g., BH690R).

**Figure 3:**
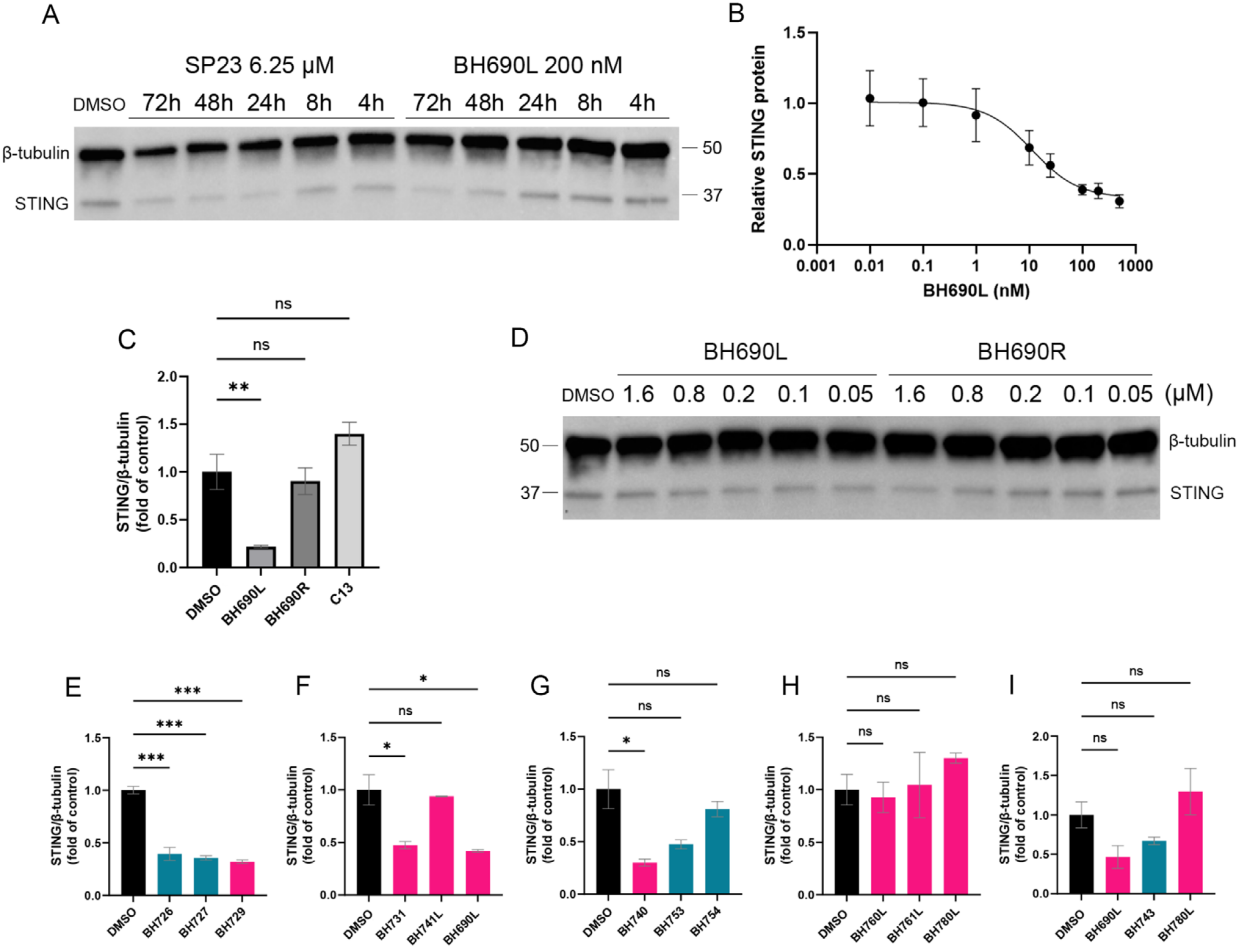
A) Time‐course comparison of SP23 and BH690L. HMC3 cells were treated with compounds for the indicated times then levels of STING were visualized by western blot. B) Dose‐response of BH690L. HMC3 cells were treated with BH690L at indicated concentrations for 72 h and levels of STING were quantified by western blot densitometry and normalized to *β*‐tubulin (*N* = 3). C) Comparison of degradation between BH690 enantiomers. HMC3 cells were treated with BH690L, BH690R, or compound 13 at 200 nM for 72 h, then levels of STING were quantified by western blot densitometry and normalized to *β*‐tubulin (*N* = 3). D) Dose‐response comparison of BH690 enantiomers. HMC3 cells were treated with either enantiomer for 72 h at indicated concentrations, then levels of STING were visualized by western blot. E–I) Screening of PROTACs. HMC3 cells were treated with either 200 nM of enantiopure or racemic mixtures of PROTACs for 72 h then quantified by western blot densitometry and normalized to *β*‐tubulin (*N* = 3). PROTACs with a *t*‐butyl vector are indicated in teal; PROTACs with a carboxylic acid vector are indicated in pink. Values are mean ± SEM. *p < 0.05, **p < 0.01, ***p < 0.001, one‐way ANOVA*.*

With analogs in hand, an initial series of western blots was conducted in human microglial clone 3 (HMC3) cells to characterize time and dose‐dependence of PROTAC‐mediated STING degradation. HMC3 is a microglial cell line, reflecting the population of immune cells active in the CNS, and implicated in retinal degeneration in inflammatory retinal disease.^[^ ^12^ ^]^ As the cell permeability of compound 13 itself is known to be poor, the activity of the PROTAC was expected to be highly dependent on incubation time. An initial series of time‐course investigations for the first analog generated (BH690L) showed delayed degradation of STING compared to SP23 (**Figure** **3**). SP23‐mediated STING degradation is known to be time‐dependent, and degradation is clearly observed at 24 h of incubation, while BH690L showed no observable degradation until the 48 h time point. While the original report of SP23 also noted time‐dependent degradation of STING, covalent inhibitors are time‐dependent by nature, so although both SP23 and BH690L demonstrate time dependency, the source of the phenomenon is likely different between the compounds.^[^ ^13^ ^]^ At 72 h, a dose–response western blot analysis of BH690L (Figure 3B) reveals an effective concentration for half‐maximal degradation (DC_50_) of 11.3 nM and a maximum level of degradation observed for a given concentration of PROTAC (D_max_) of 0.67. Interestingly, the DC_50_ is lower than the reported cell‐free IC_50_ of Compound 13 (84 nM), demonstrating the potential benefit of PROTAC catalytic turnover.

Although the absolute configuration of the enantiopure compounds is unknown, the activity of each PROTAC enantiomer was hypothesized to differ significantly. Indeed, western blot analysis revealed that BH690L is the more active enantiomer, with STING degradation observed as low as 50 nM compared to 1.6 μM for BH690R (Figure 3C,D). From this difference in activity, the levorotatory enantiomer is likely the active (*S,S*) configuration, as the absolute configuration of the active enantiomer of compound 13 was previously reported as the (*S,S*) configuration.^[^ ^11^ ^]^

Western blot analysis of the other synthesized analogs (Figure 2 and 3E–I) revealed that polyethylene glycol (PEG) linkers at either vector location were well‐tolerated (BH690L, BH726, BH727, BH729, BH731), the exception being BH743. Alkyl linkers were poorly tolerated, with BH740 being the only analog to induce degradation under the conditions tested. In the context of the *t*‐butyl vector, the failure of 8–10 carbon alkyl linkers and a PEG_3_ linker to induce STING degradation contrasted with the activity of PEG_4_ and PEG_5_ linkers, implying a minimum required linker length for activity at this linker attachment site. The carboxylic acid vector was more tolerant of varying linker lengths and chemistries, allowing an eight‐carbon alkyl linker, and PEG_3–5_ linkers. The broad tolerance from this vector suggests that the presence of unstructured amino acids in the STING “lid” does not interfere with the ternary complex formation with CRBN. BH780L, containing *N*‐methyl thalidomide, was synthesized as a negative control for CRBN recruitment. As expected, STING degradation activity was not observed in the presence of BH780L (Figure 3H,I), demonstrating that the recruitment of CRBN is critical for degradation.

Replacement of a CRBN‐recruiting ligand with a VHL‐recruiting moiety resulted in no STING degradation (BH760L and BH761L). BH760L was synthesized for direct comparison to BH690L, as the POI ligand and linkers are identical (Figure 2). This matched pair illustrates the nuances between disparate E3 ligases and the linkers (types and lengths) required for inducing degradation. Further, as STING is bound to the endoplasmic reticulum, steric issues may limit the access of different E3 ligases.

**Figure 4:**
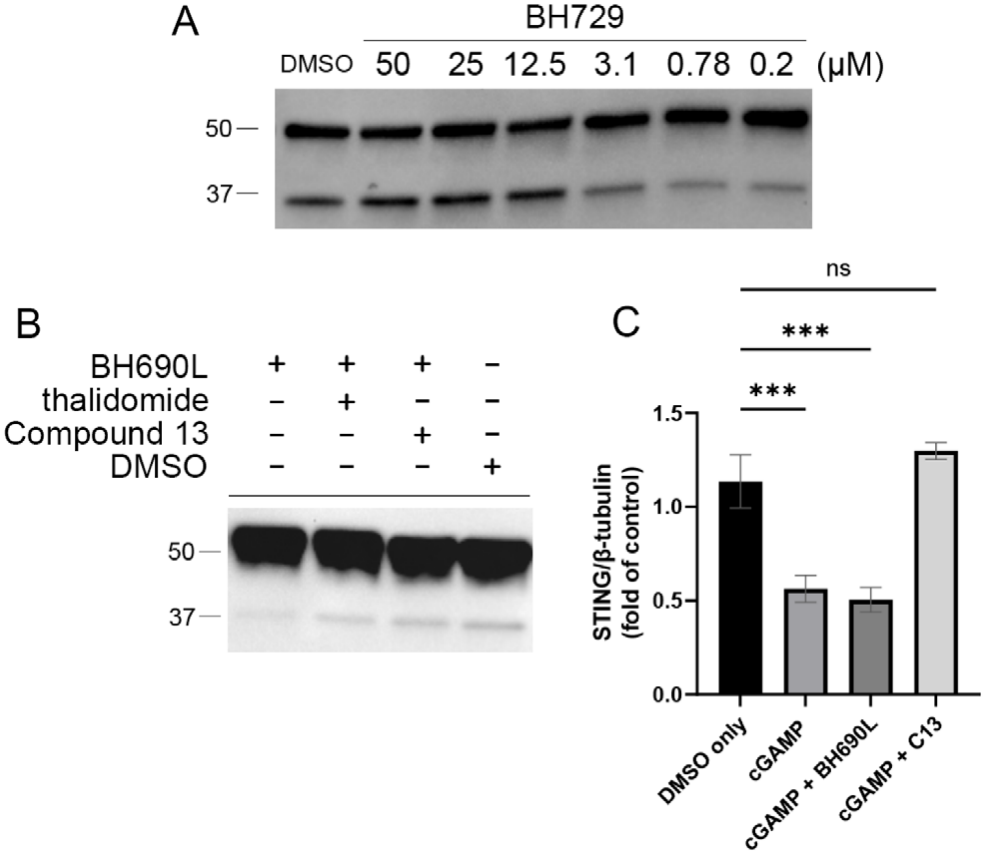
A) Hook effect in racemic BH729 PROTAC mixture. HMC3 cells are incubated with BH729 as a racemic mixture for 72 h at indicated concentrations; then, levels of STING were visualized by western blot. B) Effects of coadministration of thalidomide and compound 13 on the degradation efficiency of BH690L. HMC3 cells are treated with compound 13 at 20 μM or thalidomide at 200 μM for 72 h in the presence of BH690L. Levels of STING were visualized by western blot. C) Characterization of cGAMP‐mediated degradation. HMC3 cells were treated with BH690L or compound 13 (20 μM) for 48 h, then cGAMP was added for 24 h. Levels of STING were quantified by western blot densitometry and normalized to *β*‐tubulin (*N* = 3). Values are mean ± SEM. *p < 0.05, one‐way ANOVA.

PROTACs often demonstrate a hook effect at high concentration, as unproductive binary complexes prevent ternary complex formation, leading to less efficient protein degradation.^[^ ^7^ ^]^ Racemic PROTAC mixtures are hypothesized to be particularly susceptible to hook effect due to equipotent E3 binding from both active and inactive STING ligands. To quantify the dose ranges that separate hook effect from ternary complex formation, higher concentrations of PROTAC were used, up to 50 μM. A racemic mixture of BH729 was used for these studies to reduce the consumption of more valuable enantiopure compounds. The resulting western blot analysis reveals a pronounced hook effect beginning at 3.1 μM (**Figure** **4**A). The presence of the unproductive enantiomer did not inhibit STING degradation at mid‐nanomolar concentrations, obviating the need for time‐consuming chiral separations for every PROTAC synthesized.

To ensure STING degradation following BH690L treatment was mediated by STING and CRBN target engagement, the ability of high concentrations of target‐specific ligands were used to evaluate competitive inhibition of the PROTAC‐mediated STING degradation (Figure 4B). Both compound 13 (20 μM) and thalidomide (200 μM) are able to prevent PROTAC‐mediated degradation. Initial attempts at commonly utilized proteasome‐mediated verification methods with proteasome inhibitors (i.e., MG132, bortezomib) or ubiquitin E1 inhibitors (i.e., TAK‐243) resulted in cytotoxicity due to requisite prolonged incubation (72 h).^[^ ^14^ ^]^ Utilizing modified conditions, however, in which higher concentrations of PROTAC (800 nM) were utilized and MG132 (25 μM) or MLN4924 (10 μM), a neddylation inhibitor, were added in the final 6 h of incubation, revealed attenuated STING degradation (Figure S3, Supporting Information).^[^ ^15^ ^]^ These results provide credence to a proteasome‐mediated mechanism of degradation.

Canonical activation of STING by cGAMP binding leads to the trafficking of STING from the ER to the Golgi, and then to lysosomes where STING is degraded.^[^ ^16^ ^]^ PROTAC‐mediated STING degradation was compared to cGAMP‐mediated degradation (Figure 4C). Treatment of HMC3 cells with cGAMP (1 μM) led to successful degradation of STING; coadministration of cGAMP (1 μM) with BH690L (200 nM) did not lead to any additional degradation, as both compounds occupy the same site and exhibit maximum effect at their respective doses. Lysosomal‐mediated STING degradation induced by cGAMP is entirely abrogated by compound 13 (200 nM), as competitive displacement of cGAMP by compound 13 prevents STING activation.

**Figure 5:**
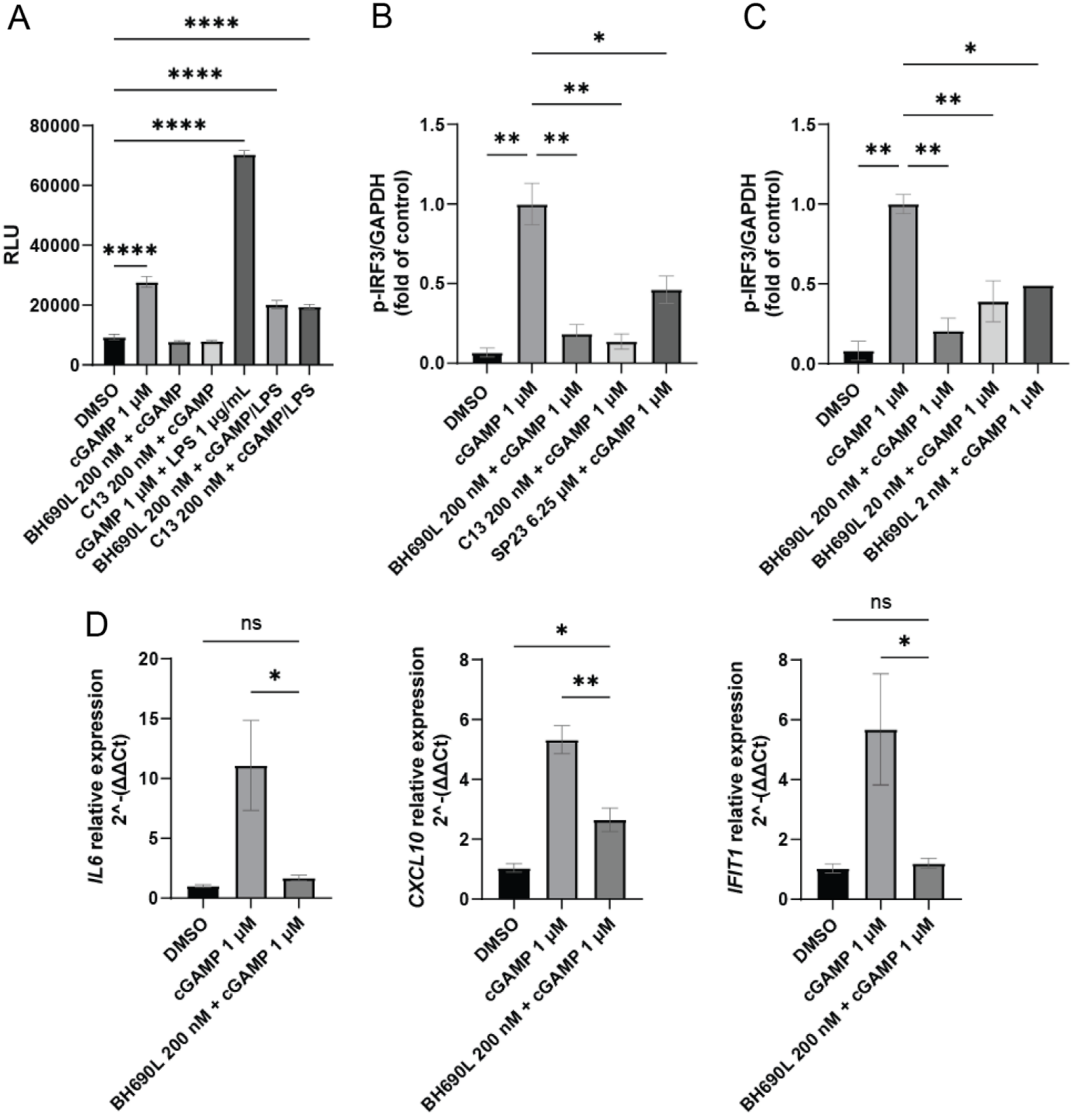
A) Quantification of IL‐6. HMC3 cells are treated with BH690L (200 nM) or compound 13 (200 nM) for 48 h, then cGAMP (1 μM) or cGAMP + LPS (10 μM^ −^^1 ^μg mL^−1^) for 24 h. IL‐6 in cell culture supernatant is then quantified by Lumit assay (*N* = 3). B) Quantification of *p*‐IRF3 following treatment with cGAMP via western blot densitometry. HMC3 cells are treated with BH690L or compound 13 for 66 h, or SP23 for 18 h. Cells were stimulated with cGAMP 1 μM for 6 h, and levels of *p*‐IRF3 were quantified by western blot densitometry and normalized to GAPDH (*N* = 2). C) Quantification of *p*‐IRF3 following treatment with indicated concentrations of BH690L. HMC3 cells were treated with BH690L for 66 h. Cells were stimulated with cGAMP for 6 h, and levels of *p*‐IRF3 are quantified by western blot densitometry and normalized to GAPDH (*N* = 2). D) Quantification of downstream inflammatory targets. HMC3 cells were treated with BH690L for 48 h, then cGAMP for 24 h. Transcripts were quantified by qPCR and normalized to GAPDH (*N* = 4). Values are mean ± SEM. *p < 0.05, **p < 0.01, ***p < 0.001, ****p < 0.0001, one‐way ANOVA.

STING signaling is well‐correlated with the downstream production of interleukin 6 (IL‐6).^[^ ^17^ ^]^ Direct quantification of IL‐6 levels in cell culture supernatant was undertaken via a Lumit immunoassay (**Figure** **5**A).^[^ ^18^ ^]^ Briefly, two antibodies targeting separate epitopes on IL‐6 are each conjugated to a different fragment of a NanoBiT luciferase; antibody binding induces proximity between these luciferase fragments, forming a functional luciferase protein. Detected luminescence thereby directly correlates with levels of secreted IL‐6. HMC3 cells were stimulated with either cGAMP 1 μM or cGAMP 10 μM + lipopolysaccharide (LPS) 1 μg mL^−^^1^ for 24 h. Both cGAMP and LPS stimulate the cGAS‐STING pathway; cGAMP is a direct STING agonist while LPS stimulates cGAS‐STING indirectly via mitochondrial dysfunction and leakage of mitochondrial DNA (mtDNA) into the cytoplasm. When pretreated with BH690L or compound 13 for 48 h, both were able to block cGAMP‐induced IL‐6 upregulation. Both compounds also reduce IL‐6 expression following cGAMP + LPS stimulation, although levels remain significantly higher than control, likely due to the additional TLR4‐mediated response from LPS via a non‐STING‐mediated pathway.^[^ ^19^ ^]^ As expected, this assay confirms no difference between the degree of inhibition for IL‐6 expression between the small molecule POI inhibitor and the PROTAC.

To further probe the downstream effects of STING inhibition versus degradation, a set of western blots was undertaken to characterize change in IRF3 phosphorylation following stimulation with cGAMP in the presence of compound 13 or BH690L (Figure 5B). STING signaling directly results in phosphorylation and activation of IRF3, which mediates the transcription of interferons.^[^ ^1^ ^]^ Hence, p‐IRF3 was chosen as a proximate downstream readout. BH690L, compound 13, and SP23 were all able to reduce levels of p‐IRF3 following 6 h of cGAMP stimulation, with no statistically significant difference between BH690L and compound 13, again implying similar downstream effects for reversible inhibition versus protein degradation. Impressively, BH690L retained p‐IRF3 reduction at concentrations as low as 2 nM in dose–response studies (Figure 5C).

To characterize a broader set of downstream proteins involved in immune signaling, qPCR was used to quantify differences in transcript counts in HMC3 cells pretreated with BH690L and stimulated with cGAMP (Figure 5D). For this study, IL‐6 was chosen as a direct product of STING activity and CXCL10 and IFIT1 were chosen as downstream products of interferon production. Relative expression of IL6, CXCL10, and IFIT1 transcripts were significantly lower with BH690L pretreated and cGAMP stimulated cells compared the expression levels in non‐BH690L pretreated cell populations stimulated with cGAMP. The Lumit assay data paired with qPCR results indicate that in the presence of BH690L, the expression of IL‐6 transcript and protein are comparable to unstimulated cells.

**Figure 6:**
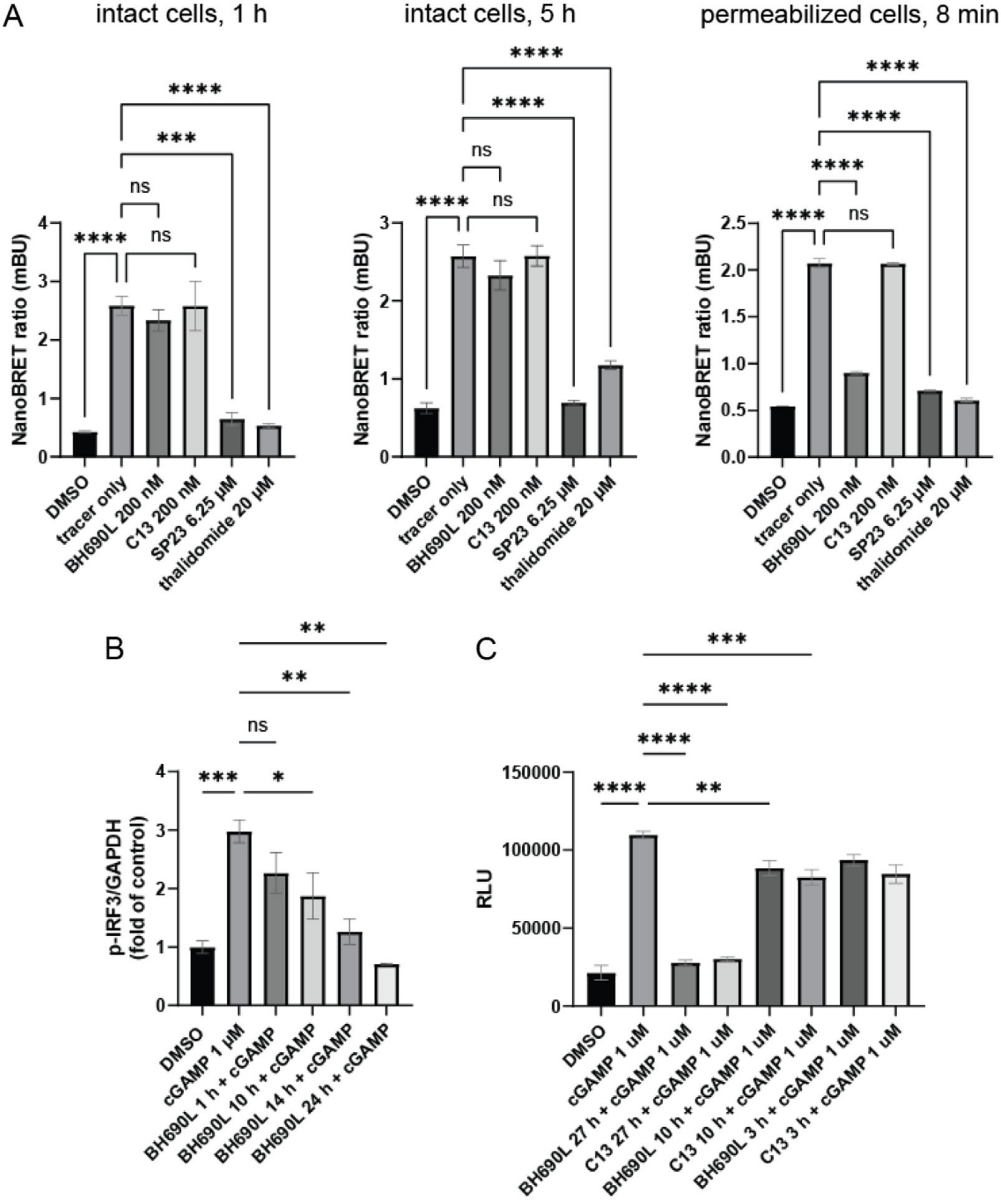
Time‐course studies. A) Quantification of membrane penetration via NanoBRET assay. HMC3 cells are incubated with tracer and/or compounds for the indicated durations. Digitonin 50 μg mL^−1^ is used to permeabilize cells. Luminescence at 450 nm/80 nm BP is measured for the donor and 610 nm LP for the acceptor, and the ratio is reported as milliBRET units (mBU) (*N* = 3). B) Quantification of p‐IRF3 following stimulation with cGAMP via western blot densitometry. HMC3 cells are treated with BH690L 200 nM for indicated durations, then stimulated with cGAMP 1 μM for 6 h, and levels of p‐IRF3 were quantified by western blot densitometry and normalized to GAPDH (*N* = 3). C) Quantification of IL‐6. HMC3 cells are treated with BH690L (200 nM) or compound 13 (200 nM) for indicated durations, then cGAMP 1 μM is added for 24 h. IL‐6 in cell culture supernatant is then quantified by Lumit assay (*N* = 3). Values are mean ± SEM. *p < 0.05, **p < 0.01, ***p < 0.001, ****p < 0.0001, one‐way ANOVA.

Although PROTACs commonly demonstrate poor membrane penetration, which we believed the likely culprit for delayed degradation, several other factors could contribute to the observed time course for STING degradation, including inefficient ternary complex formation at the endoplasmic reticulum, or steric issues with proteasome function against an ER‐bound protein. To specifically investigate the membrane penetration of BH690L, an initial PAMPA assay resulted in no detectable UV absorbance in the acceptor well after 72 h, implying membrane permeability is significantly worse than conventional small molecules (Figure S2, Supporting Information). Characterization of membrane penetration was then undertaken via a NanoBRET assay (**Figure** **6**A), in which the displacement of a bioluminescent tracer from intracellularly located CRBN results in suppression of NanoBRET acceptor signal.^[^ ^20^ ^]^ Following treatment with small molecule CRBN ligands of interest, decrease in the ratio of NanoBRET acceptor to donor signal thereby indicates successful membrane penetration. The NanoBRET assay is run with both intact cells and cells permeabilized with digitonin. Highly permeable compounds will bind CRBN in either mode, poorly permeable compounds will bind CRBN only when cells are permeabilized.

The NanoBRET assay was performed with BH690L, compound 13, SP23, and thalidomide, all at doses associated with functional activity. After 1 or 5 h of incubation with intact cells, BH690L is unable to displace tracer. When the cells are permeabilized for 8 min, BH690L successfully displaces tracer (Figure 6A). Both SP23 and thalidomide are able to penetrate intact cell membranes within 1 h, while compound 13 lacks a CRBN‐binding motif and does not displace tracer under any tested condition. This suggests that for BH690L, membrane penetration is a key barrier to PROTAC‐mediated degradation and is a significant contributor toward the delayed degradation effect. The NanoBRET assay did not detect penetration for BH690L in intact cells up to 5 h, and the assay was not amenable to further prolonging the incubation period. Instead, prolonged time course assays were performed via western blot analysis and Lumit assay. Quantification of p‐IRF3 (Figure 6B) served as a proxy for STING activation following cGAMP stimulation of cells pretreated with BH690L. BH690L shows time dependence, with no effect seen at 1 h and significant effect at 10 h, decreasing in a time‐dependent manner to 72 h. The Lumit assay (Figure 6C) showed no effect at 10 h, but a significant decrease in IL‐6 detection at 27 h. As p‐IRF3 requires only phosphorylation of existing protein, and IL‐6 requires de novo protein synthesis and accumulation in the cell culture supernatant, detection of p‐IRF3 before IL‐6 is expected. These effects are based only on STING inhibition and measure the contribution of membrane penetration toward delayed degradation. As STING degradation is seen at 48 h, other steps may also significantly delay degradation, like recruitment of ubiquitin or proteasome machinery to the ER membrane.

In summary, STING has attracted significant attention as a therapeutic target for various diseases, and novel molecules and new approaches to manipulate STING biology are desirable. In this study, we selected a previous published STING inhibitor, compound 13, to develop noncovalent PROTACs of STING. While a covalent PROTAC of STING has been disclosed previously, to the best of our knowledge successful elaboration of compound 13 to a PROTAC would provide the first reported noncovalent small molecule catalyst for STING degradation based on an inhibitory scaffold. To design target molecules, a crystal structure of a highly related molecule, compound 18, was used to identify possible locations for linker attachment that were compatible with ligand binding. Two possible points of attachment for the linker were identified. Alkyl‐ and PEG‐based linkers in varying lengths were appended containing one of two E3‐ligase ligands (CRBN or VHL) to provide a small library for screening. BH690L, containing a PEG_3_ linker and CRBN‐ligand, was identified as the most potent analog. In contrast with the more membrane permeable and covalent STING PROTAC, SP23, BH690L is slow‐acting and poorly permeable but exhibits improved potency. Initial characterization of the downstream effects of BH690L reveals inhibition of IRF3 phosphorylation, suppression of IL‐6 signaling, and decrease in interferon‐stimulated gene expression. Attenuation of STING degradation in the presence of a proteasome inhibitor (MG132) or a neddylation inhibitor (MLN4924) provides evidence of a proteasome‐mediated degradation mechanism.

The present study observed functional equivalence between traditional STING inhibition and PROTAC‐mediated degradation. A full elucidation of any potential benefit of degradation over inhibition will require additional methodologies, including in vivo approaches amenable to prolonged treatments. Additionally, only a few downstream targets were characterized, and degradation of STING is likely to have broader effects in the proteome. Our studies were conducted exclusively in microglia; however, the retinal neurovascular unit is characterized by a heterogeneous grouping of diverse cell types, including photoreceptors and endothelial cells. As the expression of both STING and E3 ligases are variable in these cell types, further work is needed to characterize the biological effects of small molecule induced STING degradation.^[^ ^21^ ^]^ The continued development of STING PROTACs based on the BH690L chemotype will likely require optimization of linker length, chemistry, and rigidification. Pertinent to this research, a dual inhibitor bidentate ligand was recently reported, consisting of Compound 13 and SN‐011, with many promising optimizations that improve potency.^[^ ^22^ ^,^ ^23^ ^]^ Remarkably, this ligand is seen to only degrade STING^S154^ and STING^M155^ mutants, providing an additional avenue for targeted STING degradation spatiotemporally.^[^ ^23^ ^]^

Although BH690L shows no additional inhibitory benefit compared to compound 13, the increased size of BH690L is expected to diminish the ability of the compound to escape the vitreous humor, and the noncovalent and therefore presumed catalytic nature of the PROTAC sets the stage for the use of BH690L as an in vivo tool to interrogate PROTAC strategies in the eye.

## Supporting Information

The supporting information is available free of charge.

Biological assay protocols, CRBN and STING expression profiles in retinal cells, BH690 PAMPA results, western blots of MG132 and MLN4924‐mediated attenuation of PROTAC‐mediated degradation, supplemental synthetic scheme, synthetic procedures, NMR spectra for all final compounds, and HPLC traces.

## Conflict of Interest

The authors declare no conflict interest.

## Author Contributions

**Bo Hu** and **Adam S. Duerfeldt** designed all compounds and **Bo Hu** synthesized all molecules and conducted all biological assessment. The manuscript was written and edited by **Bo Hu** and **Adam S. Duerfeldt** and approved by both authors.

## Supporting information

Supplementary Material

## Data Availability

The data that support the findings of this study are available from the corresponding author upon reasonable request.

## References

[1] M. Motwani , S. Pesiridis , K. A. Fitzgerald , Nat. Rev. Genet. 2019, 20, 657. 31358977 10.1038/s41576-019-0151-1

[2] A. Decout , J. D. Katz , S. Venkatraman , A. Ablasser , Nat. Rev. Immunol. 2021, 21, 548. 33833439 10.1038/s41577-021-00524-z PMC8029610

[3] N. Sasaki , M. Homme , S. Kitajima , Cancer Sci. 2023, 114, 3806. 37475576 10.1111/cas.15913 PMC10551601

[4] J. C. Martinez , F. Morandini , L. Fitzgibbons , N. Sieczkiewicz , S. J. Bae , M. E. Meadow , E. Hillpot , J. Cutting , V. Paige , S. A. Biashad , M. Simon , J. Sedivy , A. Seluanov , V. Gorbunova , *bioRxiv* 2024, October 11, 2024, 10.1101/2024.10.10.617645.

[5] B. Hu , J.‐X. Ma , A. S. Duerfeldt , Future Med. Chem. 2023, 15, 717. 37166075 10.4155/fmc-2022-0301 PMC10194038

[6] E. M. D. Amo , A.‐K. Rimpelä , E. Heikkinen , O. K. Kari , E. Ramsay , T. Lajunen , M. Schmitt , L. Pelkonen , M. Bhattacharya , D. Richardson , A. Subrizi , T. Turunen , M. Reinisalo , J. Itkonen , E. Toropainen , M. Casteleijn , H. Kidron , M. Antopolsky , K.‐S. Vellonen , M. Ruponen , A. Urtti , Prog. Retinal Eye Res. 2017, 57, 134. 10.1016/j.preteyeres.2016.12.001 28028001

[7] M. Békés , D. R. Langley , C. M. Crews , Nat. Rev. Drug Discov. 2022, 21, 181. 35042991 10.1038/s41573-021-00371-6 PMC8765495

[8] J. Zhang , Y. Qi , Y. Li , F. Zhu , Y. Geng , Y. Li , B. Xue , H. Bi , Y. Jiao , H. Min , D. Jiang , G. Nie , Y. Qi , J. Controlled Release 2025, 381, 113567. 10.1016/j.jconrel.2025.02.063 39993640

[9] X. Zhu , W. Liu , X. Tang , Y. Chen , X. Ge , Q. Ke , X. Liang , Y. Gan , Y. Zheng , M. Zou , M. Deng , Y. Liu , D. W.‐C. Li , L. Gong , J. Neuroinflammation 2023, 20, 119. 37217935 10.1186/s12974-023-02804-y PMC10201800

[10] J. Liu , L. Yuan , Y. Ruan , B. Deng , Z. Yang , Y. Ren , L. Li , T. Liu , H. Zhao , R. Mai , J. Chen , J. Med. Chem. 2022, 65, 6593. 35452223 10.1021/acs.jmedchem.1c01948

[11] T. Siu , M. D. Altman , G. A. Baltus , M. Childers , J. M. Ellis , H. Gunaydin , H. Hatch , T. Ho , J. Jewell , B. M. Lacey , C. A. Lesburg , B.‐S. Pan , B. Sauvagnat , G. K. Schroeder , S. Xu , ACS Med. Chem. Lett. 2019, 10, 92. 30655953 10.1021/acsmedchemlett.8b00466 PMC6331172

[12] P. Quiriconi , V. Hristov , M. Aburaya , U. Greferath , A. I. Jobling , E. L. Fletcher , npj Metab. Health Dis. 2024, 2, 7. 40603568 10.1038/s44324-024-00009-2 PMC12118658

[13] J. M. Strelow , SLAS Discovery 2017, 22, 3. 27703080 10.1177/1087057116671509

[14] M. P. Schwalm , A. Menge , L. Elson , F. A. Greco , M. B. Robers , S. Müller , S. Knapp , ACS Chem. Biol. 2025, 20, 94. 39753207 10.1021/acschembio.4c00450 PMC11745162

[15] F. Meng , C. Xu , K.‐S. Park , H.Ü. Kaniskan , G. G. Wang , J. Jin , J. Med. Chem. 2022, 65, 10611. 35895319 10.1021/acs.jmedchem.2c00807 PMC9378504

[16] M. Gentili , B. Liu , M. Papanastasiou , D. Dele‐Oni , M. A. Schwartz , R. J. Carlson , A. M. Al’Khafaji , K. Krug , A. Brown , J. G. Doench , S. A. Carr , N. Hacohen , Nat. Commun. 2023, 14, 611. 36739287 10.1038/s41467-023-36132-9 PMC9899276

[17] C. Hong , M. Schubert , A. E. Tijhuis , M. Requesens , M. Roorda , A. Van Den Brink , L. A. Ruiz , P. L. Bakker , T. Van Der Sluis , W. Pieters , M. Chen , R. Wardenaar , B. Van Der Vegt , D. C. J. Spierings , M. De Bruyn , M. A. T. M. Van Vugt , F. Foijer , Nature 2022, 607, 366. 35705809 10.1038/s41586-022-04847-2

[18] B. B. Hwang , J. Alves , D. Lazar , N. Nath , L. Engel , M. O’Brien , K. Hsiao , K. Kupcho , B. Godat , R. Flemming , S. Goueli , H. Zegzouti , Methods Mol. Biol. 2023, 2612, 195. 36795369 10.1007/978-1-0716-2903-1_15

[19] A. Tesser , G. M. Piperno , A. Pin , E. Piscianz , V. Boz , F. Benvenuti , A. Tommasini , Cells 2021, 10, 785. 33916318 10.3390/cells10040785 PMC8067196

[20] T. Machleidt , C. C. Woodroofe , M. K. Schwinn , J. Méndez , M. B. Robers , K. Zimmerman , P. Otto , D. L. Daniels , T. A. Kirkland , K. V. Wood , ACS Chem. Biol. 2015, 10, 1797. 26006698 10.1021/acschembio.5b00143

[21] M. Karlsson , C. Zhang , L. Méar , W. Zhong , A. Digre , B. Katona , E. Sjöstedt , L. Butler , J. Odeberg , P. Dusart , F. Edfors , P. Oksvold , K. Von Feilitzen , M. Zwahlen , M. Arif , O. Altay , X. Li , M. Ozcan , A. Mardinoglu , L. Fagerberg , J. Mulder , Y. Luo , F. Ponten , M. Uhlén , C. Lindskog , Sci. Adv. 2021, 7, eabh2169. 34321199 10.1126/sciadv.abh2169 PMC8318366

[22] Z. Hong , J. Mei , C. Li , G. Bai , M. Maimaiti , H. Hu , W. Yu , L. Sun , L. Zhang , D. Cheng , Y. Liao , S. Li , Y. You , H. Sun , J. Huang , X. Liu , J. Lieberman , C. Wang , Proc. Natl. Acad. Sci. 2021, 118, e2105465118. 34099558 10.1073/pnas.2105465118 PMC8214703

[23] H.‐Y. Zhao , L. Zhang , Z. Liu , M. He , M. Wang , Q. Li , F. Sarkari , J. Tao , B. Wen , V. Basrur , H. Myatt , A. Nesvizhskii , D. Sun , J. Med. Chem. 2025, 68, 11100. 40386971 10.1021/acs.jmedchem.5c00123

