# Supplementary material for: Development and Characterization of a Noncovalent Stimulator of Interferon Genes Proteolysis‐Targeting Chimeras
Source: ChemMedChem. 2025 Nov 18;21(1):e202500715. doi: 10.1002/cmdc.202500715 (PMC12811993; doi:10.1002/cmdc.202500715)

SUPPLEMENTARY INFORMATION
Development and Characterization of a Non-covalent STING PROTAC

Bo Hu[1] and Adam S. Duerfeldt[1*]

[1]*Department of Medicinal Chemistry, College of Pharmacy, University of Minnesota, Minneapolis, MN 55414, United States*

TABLE OF CONTENTS

I.	Biological Procedures	1
II.	Chemistry Details	2
III.	Supplemental Synthetic Scheme	3
IV.	Supplemental Figures	3
V.	Synthetic Procedures	6
VI.	NMR spectra	22
VII.	HPLC traces and references	37

BIOLOGICAL PROCEDURES

Cell culture

HMC3 cells (ATCC, CRL-3304) were cultured in EMEM media (ATCC, 30-2003) supplemented with 10% heat-inactivated FBS (Cytiva, SH30071.03HI) and 1% penicillin–streptomycin (Gibco, 15140122). Cells were then incubated in T75 flasks (Corning, 430641U) at 37 °C and 5% CO2 and passaged every three days according to manufacturer's protocol. Two days after seeding, the culture medium was replaced. Cells of passages 8-18 were used in the respective assays.

Western Blot

HMC3 cells were cultured in a 12-well plate (Corning, 3513) and treated with DMSO (control) or compounds of interest at desired concentrations. The cells were incubated at 37 °C with a 5% CO_2 atmosphere for reported time points, and lysed with RIPA buffer (Thermo Scientific, 89901) containing Pierce Protease Inhibitor (Thermo, A32955) and PhosStop (Roche, 4906837001). Each well is washed twice with 600 µL cold PBS and lysed with 54 µL of lysis buffer. The lysate was collected, mixed with 1.4 µL NuPAGE Sample Reducing Agent (Thermo, NP0009) and 3.3 µL Laemmli Sample Buffer (Bio-Rad, 1610747), heated at 95 °C for 5 min, resolved by electrophoresis through 4-15% Mini-PROTEAN TGX Stain-Free gels (Bio-Rad, 4568085), and electrotransferred onto a 0.2 µm nitrocellulose membrane (Bio-Rad, 1704158) with a Trans-Blot Turbo Transfer System (Bio-Rad, 1704150). The membrane was incubated with EveryBlot Blocking Buffer (Bio-Rad, 12010020) for 1 h, then incubated with primary antibody for 18 h at 4 °C. The membrane was then washed and incubated with anti-rabbit IgG HRP secondary antibody (Cell Signaling Technology, 7074S) for 1 h and the bands were visualized with Clarity ECL substrate (Bio-Rad, 1705060). Bands were quantified by densitometry using Image Lab 6.1.

RT-qPCR

RNA was extracted and purified from HMC3 cells with a ReliaPrep RNA Cell Miniprep System (Promega, Z6010) following the manufacturer's protocol. Purity of RNA was measured by Nanodrop for A260/A280 ≥ 2.0. RNA was then reverse transcribed into cDNA by GoTaq 2-Step RT-qPCR System (Promega, A6010) following the manufacturer's protocol. The cDNA was thermocycled with an Applied Biosystems QuantStudio 7 Pro with the following parameters: 50 °C, 2 min, 1 cycle; 95 °C 2 min, 1 cycle; 95 °C, 15 s, 40 cycles; 55 °C, 15 s, 40 cycles, 72 °C, 1 min, 40 cycles. The relative expression of target genes was normalized to GAPDH and expressed as $2^{-\Delta\Delta Ct}$.

Primer sequences

GAPDH
ACAACTTTGGTATCGTGGAAGG
GCCATCACGCCACAGTTTC

CXCL10
GTGGCATTCAAGGAGTACCTC
TGATGGCCTTCGATTCTGGATT

IFIT1
GCGCTGGGTATGCGATCTC
CAGCCTGCCTTAGGGGAAG

IL6
CCTGAACCTTCCAAAGATGGC

TTCACCAGGCAAGTCTCCTCA

Lumit IL-6 immunoassay (Promega, W6030)
HMC3 cells were cultured in a 96 well plate (SPL, 30196) at 8,000 cells per well and compounds of interest were added. The plate was incubated at 37 °C and 5% CO_2 for 48 h. cGAMP 1 μM was then added and plates were incubated for an additional 24 h. From each well, 80 μL of supernatant was collected and the Lumit assay was run per the manufacturer's protocol. Luminescence was recorded using a Glomax Explorer.

NanoBRET (Promega, N2910)
HMC3 cells were grown to 70% confluence in a 6-well plate and transfected with Lipofectamine 3000 (Thermo, L3000001) per the manufacturer's instructions. After 24 h of incubation at 37 °C with a 5% CO_2 atmosphere, cells were trypsinized, diluted with Opti-MEM (Thermo, 31985070), and 12,000 cells were plated in each well of an NBS-treated 96-well plate (Corning, 3990). The NanoBRET assay was then run per the manufacturer's instructions. Luminescence was recorded with a Clariostar using donor 450/80 nm BP and acceptor 610 nm LP.

Statistical analysis
All statistical analyses were performed using Prism 7.0c (GraphPad Software). Comparison between multiple groups was performed by one-way ANOVA. Comparison between pairs of groups was performed by Student's *t*-test. Unless otherwise stated, values were obtained in triplicate. Values of $p \leq 0.05$ were considered significant and values lower than $p \leq 0.05$ are denoted accordingly with the accompanying data.

<u>CHEMISTRY</u>
General Information: Starting materials, ACS grade methylene chloride (DCM), methanol (MeOH), hexanes, ethyl acetate (EtOAc), acetone, acetonitrile (CH_3CN), dimethyl formamide (DMF), ethanol, tetrahydrofuran (THF), toluene, and trifluoroacetic acid (TFA) were purchased from Ambeed, TCI, Oakwood, Alfa Aesar, Fisher Scientific, or Sigma Aldrich. All commercially available solvents and reagents were used without further purification. Deionized water was used for all experimental procedures where "water" is indicated. All reactions requiring anhydrous conditions were run under a nitrogen atmosphere. Analytical thin-layer chromatography (TLC) was performed on silica gel 60 F_{254} plates (Sigma-Aldrich 1.05715). Flash column chromatography was performed on a Buchi Pure C-815 flash system using prepacked Buchi Ecoflex columns. NMR was performed on a 400 MHz Bruker Avance, 600 MHz Bruker Avance NEO, or 850 MHz Bruker Avance III. All NMR data were processed in MestReNova. High-resolution mass spectrometry was performed on a Sciex X500R QTOF-MS and analyzed at the Mass Spectrometry Facility at the University of Minnesota. All final compounds are >95% pure by HPLC. All yields are unoptimized.

Supplemental Scheme 1

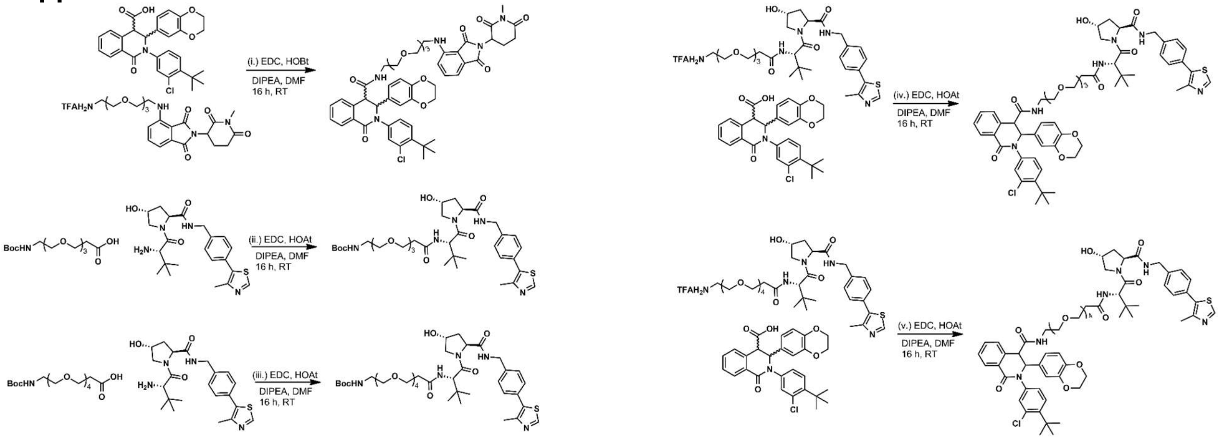

Scheme S1. Synthetic approach to additional molecules not articulated in the manuscript. (i.) EDC, HOBt, DIPEA, DMF, RT, 16 h, 70%; (ii.) EDC, HOAt, DIPEA, DMF, RT, 16 h, 64%; (iii.) EDC, HOAt, DIPEA, DMF, RT, 16 h, 62%; (iv.) EDC, HOAt, DIPEA, DMF, RT, 16 h, 48%; (v.) EDC, HOAt, DIPEA, DMF, RT, 16 h, 72%.

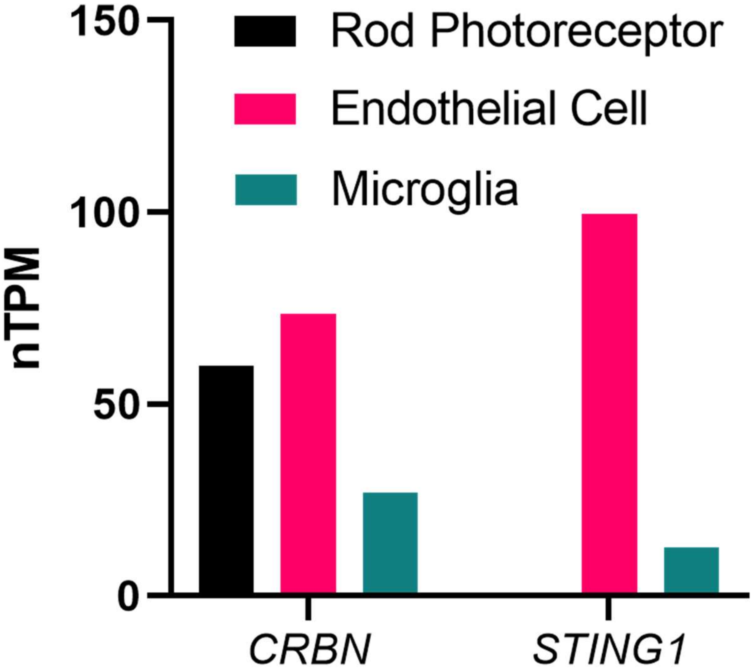

Figure S1. Single-cell RNA expression of *CRBN* and *STING1* in several cell types implicated in inflammatory retinal disease. *CRBN* is considered ubiquitously expressed although *STING1* shows higher variation between these cell types. Notably, *STING1* is nearly absent in rod photoreceptors (0.3 nTPM).

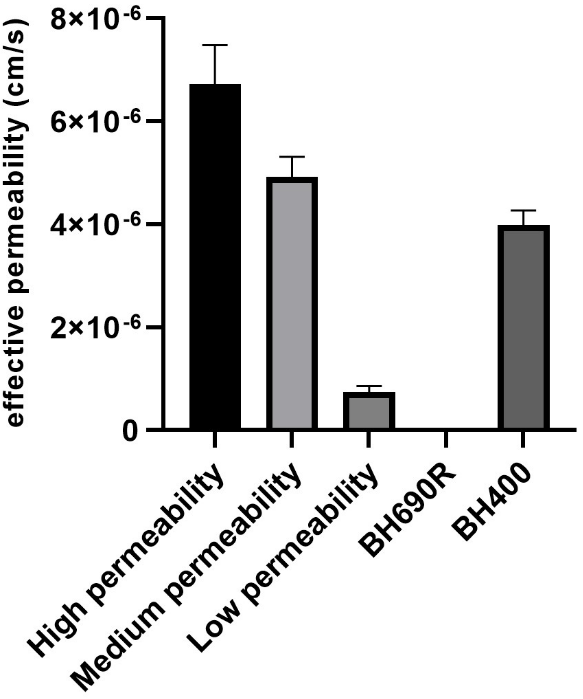

Figure S2. Parallel artificial membrane permeability assay (PAMPA) of BH690R and a druglike small molecule STING inhibitor BH400. Compared to three permeability controls, permeability of BH690L at 24 or 72 h in the PAMPA acceptor wells is below the limit of detection by UV absorbance (~12.5 µM).

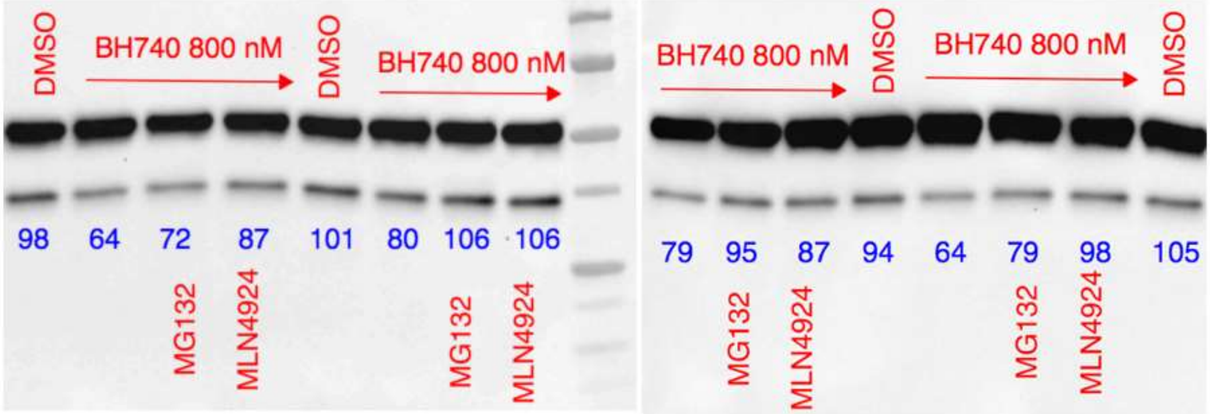

Figure S3. Attenuation of PROTAC-mediated degradation by proteasome inhibition (MG132) or neddylation inhibition (MLN4924). HMC3 cells were incubated with BH740 for 30 hours, then MG132 (5 µM) or MLN4924 (10 µM) were added and incubated for 6 hours. STING was quantified (blue, % of DMSO) by western blot densitometry and normalized to β-tubulin (N = 3). BH740 at 800 nM was used for these studies due to more robust responses at a 36 hour time point.

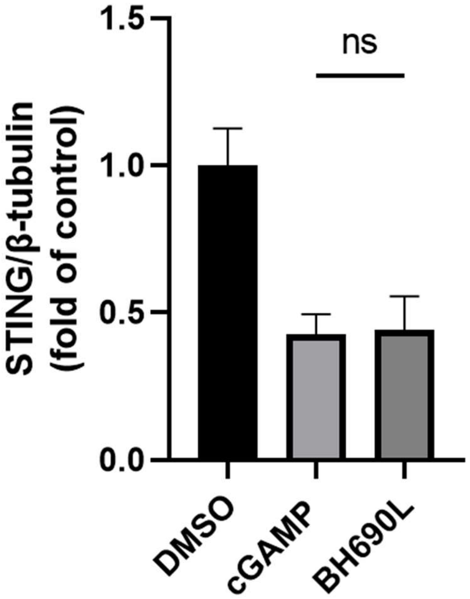

Figure S4. Comparison of small molecule degradation mediated by BH690L or cGAMP. HMC3 cells were treated with DMSO, BH690L (200 nM) for 72 hours, or cGAMP (1 μM) for 24 hours. Levels of STING were quantified by western blot densitometry and normalized to β-tubulin (N = 3). Values are mean ± SEM. *P < 0.05, Student's *t*-test.

Synthetic Procedures

General Procedure 1: *Castagnoli-Cushman reaction*. In a 20 mL scintillation vial, a mixture of homophthalic acid (1 eq), aniline (1 eq), and aldehyde (1 eq) in toluene (5 mL) was stirred at 110 °C for 16 h. The solvent was boiled off under a stream of nitrogen gas. The residue was dissolved in EtOAc and washed with 0.1 M aq. HCl and brine. The organics were collected, dried over anhydrous sodium sulfate, filtered, and concentrated under reduced pressure by rotary evaporation. The crude residue was purified by flash column chromatography (SiO_2).

General Procedure 2: *Amide coupling to access PROTACs.* In a 20 mL scintillation vial, a solution of carboxylic acid (1 eq) in DMF (5 mL) was stirred. DIPEA (2.4 eq) was added, then EDC (1.2 eq), and HOAt or HOBt (1.3 eq) were added, and the solution was stirred for 20 min. A solution of amine (1 eq) in DMF (2 mL) was then added, and the reaction mixture was stirred for 16 h at room temperature. The reaction mixture was quenched with deionized water, extracted with EtOAc. The organics were collected, washed with 0.1 M aq. HCl, saturated sodium bicarbonate, and brine. The organic phase was dried over anhydrous sodium sulfate, filtered, and concentrated under reduced pressure by rotary evaporation. The crude residue was purified by flash column chromatography (SiO_2).

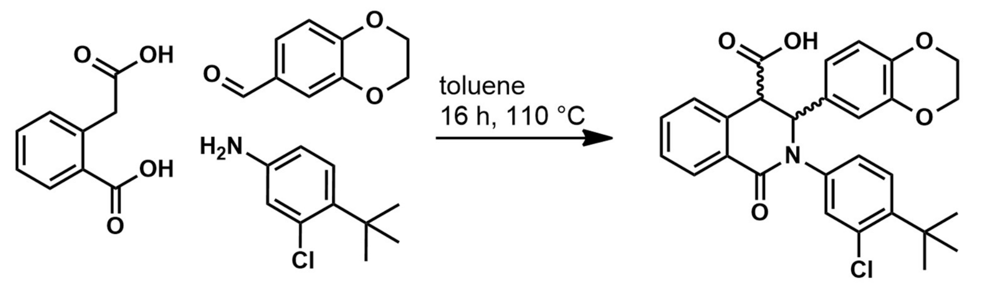

(3S,4S)-2-(4-(tert-butyl)-3-chlorophenyl)-3-(2,3-dihydrobenzo[b][1,4]dioxin-6-yl)-1-oxo-1,2,3,4-tetrahydroisoquinoline-4-carboxylic acid was synthesized in accordance with General Procedure 1, employing homophthalic acid (118 mg, 0.653 mmol), 4-tert-butyl-3-chlorobenzenamine (120 mg, 0.653 mmol), 2,3-dihydro-1,4-benzodioxin-6-carbaldehyde (107 mg, 0.65 mmol). The product was obtained in 48% yield (154 mg) as a yellow amorphous solid. ^{1}H NMR (400 MHz, DMSO-d_6) δ 7.98 (dd, *J* = 7.8, 1.5 Hz, 1H), 7.53 – 7.40 (m, 4H), 7.33 – 7.29 (m, 1H), 7.25 (dd, *J* = 8.6, 2.4 Hz, 1H), 6.72 (d, *J* = 8.4 Hz, 1H), 6.68 (d, *J* = 2.3 Hz, 1H), 6.63 (d, *J* = 10.5 Hz, 1H), 5.64 (s, 1H), 4.14 (s, 5H), 1.43 (s, 9H).

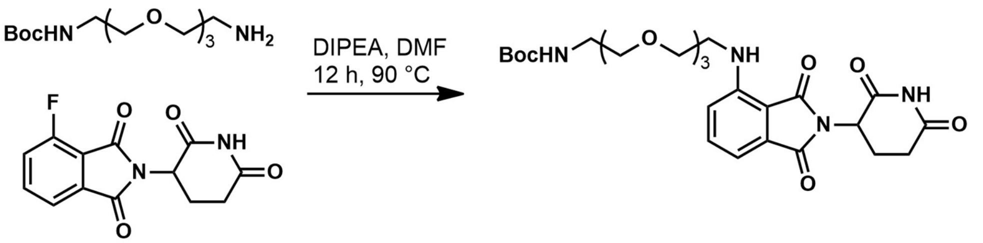

tert-butyl (2-(2-(2-(2-((2-(2,6-dioxopiperidin-3-yl)-1,3-dioxoisoindolin-4-yl)amino)ethoxy)ethoxy)ethoxy)ethyl)carbamate. DIPEA (166 mg, 1.26 mmol), tert-butyl (2-(2-(2-(2-aminoethoxy)ethoxy)ethoxy)ethyl)carbamate (250 mg, 0.85 mmol), and 2-(2,6-dioxopiperidin-3-yl)-4-fluoroisoindoline-1,3-dione (236 mg, 0.85 mmol) were stirred in 2.5 mL DMF at 90 °C for 12 h. The reaction was quenched with 25 mL water and extracted with EtOAc. The organics were collected, dried over anhydrous sodium sulfate, filtered, and concentrated under reduced pressure by rotary evaporation. The crude residue was purified by column chromatography (SiO_2, eluted with a gradient of 0-100% EtOAc in hexanes). The product was obtained in 51% yield (239 mg) as a yellow amorphous solid. ^{1}H NMR (400 MHz, Chloroform-*d*) δ^{1}H NMR (400 MHz, Chloroform-*d*) δ 8.35 (s, 1H), 7.49 (dd, *J* = 8.5, 7.1 Hz, 1H), 7.10 (d, *J* = 7.0 Hz, 1H), 6.92 (d, *J* = 8.5 Hz, 1H), 6.49 (s, 1H), 5.10 (s, 1H), 4.91 (dd, *J* = 12.0, 5.3 Hz, 1H), 3.72 (t, *J* = 5.4 Hz, 2H), 3.67 (s, 3H), 3.66 (d, *J* = 2.7 Hz, 2H), 3.64 – 3.59 (m, 3H), 3.53 (t, *J* = 5.1 Hz, 2H), 3.46 (t, *J* = 5.4 Hz, 2H), 3.30 (s, 2H), 2.91 – 2.67 (m, 4H), 2.11 (ddd, *J* = 9.5, 5.0, 2.5 Hz, 1H), 1.43 (s, 9H). To the isolated intermediate, 4 mL of a 3:1 v/v mixture of DCM:TFA was added and stirred for 3 h at 35 °C. The solvent was then evaporated under a stream of N_2 and the crude TFA salt used without further purification.

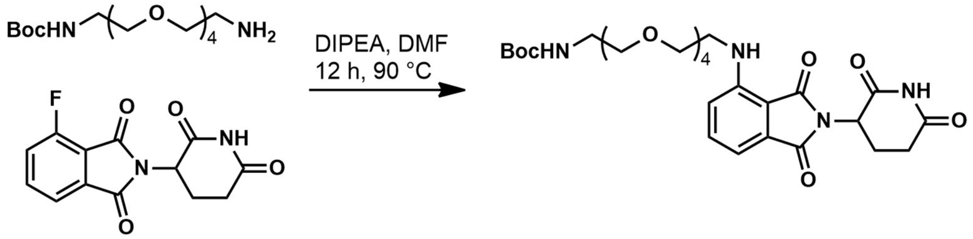

tert-butyl (14-((2-(2,6-dioxopiperidin-3-yl)-1,3-dioxoisoindolin-4-yl)amino)-3,6,9,12-tetraoxatetradecyl)carbamate. DIPEA (288 mg, 2.23 mmol), O-(2-aminoethyl)-O'-[2-(Boc-amino)ethyl]triethylene glycol (500 mg, 1.49 mmol), and 2-(2,6-dioxopiperidin-3-yl)-4-fluoroisoindoline-1,3-dione (411 mg, 1.49 mmol) were stirred in 5 mL DMF at 90 °C for 12 h. The reaction was quenched with 25 mL water, extracted with EtOAc. The organics were collected, dried over anhydrous sodium sulfate, filtered, and concentrated under reduced pressure by rotary evaporation. The crude residue was purified by column chromatography (SiO_2, eluted with a gradient of 0-100% EtOAc in hexanes followed by 0-50% acetone in EtOAc). The product was obtained in 17% yield (151 mg) as a yellow amorphous solid. ^1H NMR (400 MHz, Chloroform-*d*) δ 8.35 (s, 1H), 7.49 (dd, *J* = 8.5, 7.1 Hz, 1H), 7.10 (d, *J* = 7.0 Hz, 1H), 6.92 (d, *J* = 8.5 Hz, 1H), 6.49 (s, 1H), 5.10 (s, 1H), 4.91 (dd, *J* = 12.0, 5.3 Hz, 1H), 3.72 (t, *J* = 5.4 Hz, 2H), 3.67 (s, 4H), 3.67 – 3.64 (m, 3H), 3.64 – 3.59 (m, 4H), 3.53 (t, *J* = 5.1 Hz, 2H), 3.46 (t, *J* = 5.4 Hz, 2H), 3.30 (s, 2H), 2.92 – 2.67 (m, 3H), 2.17 – 2.08 (m, 1H), 1.43 (s, 9H). To the isolated intermediate, 4 mL of a 3:1 v/v mixture of DCM:TFA was added and stirred for 3 h at 35 °C. The solvent was then evaporated under a stream of N_2 and the crude TFA salt used without further purification.

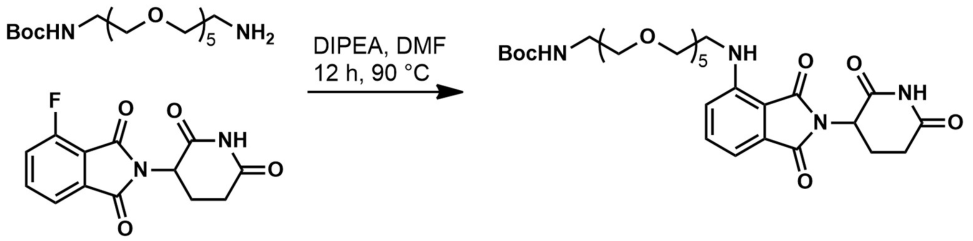

tert-butyl (17-((2-(2,6-dioxopiperidin-3-yl)-1,3-dioxoisoindolin-4-yl)amino)-3,6,9,12,15-pentaoxaheptadecyl)carbamate. DIPEA (255 mg, 1.98 mmol), O-(2-aminoethyl)-O'-[2-(Boc-amino)ethyl]tetraethylene glycol (500 mg, 1.32 mmol), and 2-(2,6-dioxopiperidin-3-yl)-4-fluoroisoindoline-1,3-dione (364 mg, 1.32 mmol) were stirred in 5 mL DMF at 90 °C for 12 h. The reaction was quenched with 25 mL water, extracted with EtOAc. The organics were collected, dried over anhydrous sodium sulfate, filtered, and concentrated under reduced pressure by rotary evaporation. The crude residue was purified by column chromatography (SiO_2, eluted with a gradient of 50-100% EtOAc in hexanes followed by 0-50% acetone in EtOAc). The product was obtained in 25% yield (214 mg) as a yellow amorphous solid. ^1H NMR (400 MHz, Chloroform-*d*) δ 8.44 (s, 1H), 7.48 (dd, *J* = 8.5, 7.1 Hz, 1H), 7.09 (d, *J* = 7.1 Hz, 1H), 6.91 (d, *J* = 8.5 Hz, 1H), 6.48 (s, 1H), 5.07 (s, 1H), 4.90 (dd, *J* = 11.9, 5.3 Hz, 1H), 3.71 (t, *J* = 5.4 Hz, 2H), 3.69 – 3.58 (m, 16H), 3.52 (t, *J* = 5.2 Hz, 2H), 3.46 (d, *J* = 5.0 Hz, 2H), 3.29 (d, *J* = 5.4 Hz, 2H), 2.90 – 2.82 (m, 1H), 2.82 – 2.71 (m, 2H), 2.15 – 2.08 (m, 1H), 1.43 (s, 9H). To the isolated intermediate, 4 mL of a 3:1 v/v mixture of DCM:TFA was added and stirred for 3 h at 35 °C. The solvent was then evaporated under a stream of N_2 and the crude TFA salt used without further purification.

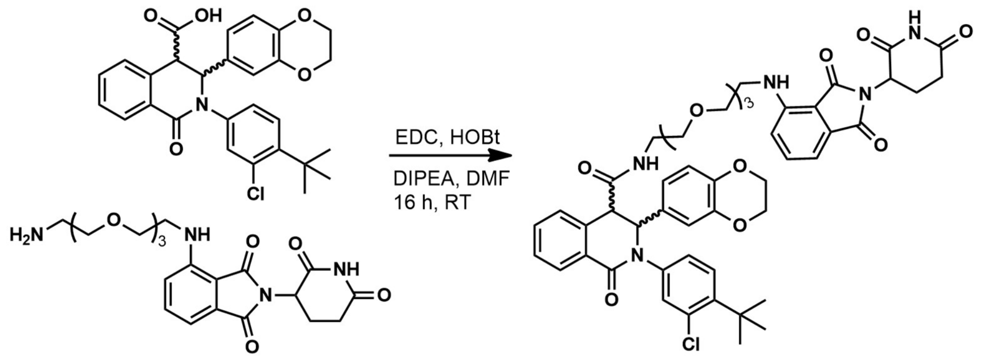

2-(4-(tert-butyl)-3-chlorophenyl)-3-(2,3-dihydrobenzo[b][1,4]dioxin-6-yl)-N-(2-(2-(2-(2-((2-(2,6-dioxopiperidin-3-yl)-1,3-dioxoisoindolin-4-yl)amino)ethoxy)ethoxy)ethoxy)ethyl)-1-oxo-1,2,3,4-tetrahydroisoquinoline-4-carboxamide (BH690) was synthesized in accordance with General Procedure 2, employing 4-((2-(2-(2-(2-aminoethoxy)ethoxy)ethoxy)ethyl)amino)-2-(2,6-dioxopiperidin-3-yl)isoindoline-1,3-dione (40 mg, 0.089 mmol), 2-(4-(tert-butyl)-3-chlorophenyl)-3-(2,3-dihydrobenzo[b][1,4]dioxin-6-yl)-1-oxo-1,2,3,4-tetrahydroisoquinoline-4-carboxylic acid (44 mg, 0.089 mmol), EDC (21 mg, 0.11 mmol), HOBt (18 mg, 0.12 mmol), and DIPEA (28 mg, 0.21 mmol). The product was obtained in 60% yield (49 mg) as a yellow amorphous solid. ^{1}H NMR (400 MHz, DMSO-d_6) δ 11.09 (s, 1H), 8.27 (t, J = 5.6 Hz, 1H), 7.97 (dd, J = 7.6, 1.6 Hz, 1H), 7.56 (dd, J = 8.6, 7.1 Hz, 1H), 7.50 – 7.38 (m, 3H), 7.34 (d, J = 2.3 Hz, 1H), 7.27 – 7.23 (m, 1H), 7.16 – 7.10 (m, 2H), 7.03 (d, J = 7.0 Hz, 1H), 6.75 (d, J = 2.2 Hz, 1H), 6.72 (d, J = 8.4 Hz, 1H), 6.67 – 6.62 (m, 1H), 6.59 (t, J = 5.8 Hz, 1H), 5.32 (s, 1H), 5.05 (dd, J = 12.9, 5.4 Hz, 1H), 4.15 (s, 4H), 4.07 (d, J = 1.7 Hz, 1H), 3.59 (t, J = 5.4 Hz, 2H), 3.55 – 3.51 (m, 1H), 3.49 (d, J = 5.1 Hz, 1H), 3.44 (q, J = 3.8, 3.2 Hz, 2H), 3.43 – 3.35 (m, 2H), 3.23 (t, J = 5.8 Hz, 1H), 2.87 (ddd, J = 18.6, 13.9, 5.3 Hz, 1H), 2.57 (d, J = 15.8 Hz, 1H), 2.06 – 1.96 (m, 1H), 1.41 (s, 7H), 1.23 (s, 1H). ^{13}C NMR (214 MHz, DMSO) δ 173.2, 170.6, 170.5, 169.4, 167.8, 163.3, 146.9, 144.2, 143.7, 143.2, 142.1, 136.7, 135.0, 133.3, 132.9, 132.6, 132.5, 130.1, 129.5, 128.9, 128.6, 128.4, 127.9, 125.0, 119.2, 117.9, 117.5, 115.3, 111.2, 109.7, 70.2 (2C), 70.2, 70.1, 69.4, 69.4, 64.7, 64.5, 64.4, 51.6, 49.0, 42.2, 40.3, 40.2, 40.1, 40.0, 39.9, 39.8, 39.7, 39.4, 36.0, 31.4, 29.8 (3C), 22.6. HRMS (ESI, m/z) for $C_{49}H_{52}ClN_5O_{11}$ [M + Na]: expected, 944.3244; found, 944.3250; 0.6 ppm. The mixture of trans enantiomers was further purified by chiral HPLC (Phenomenex Lux Cellulose-1 column, 100% MeCN) to afford the separate isomers. BH690L: $[\alpha]_D^{25}$ -2.6 (*c* 1, MeOH). BH690R: $[\alpha]_D^{25}$ +4.4 (*c* 1, MeOH).

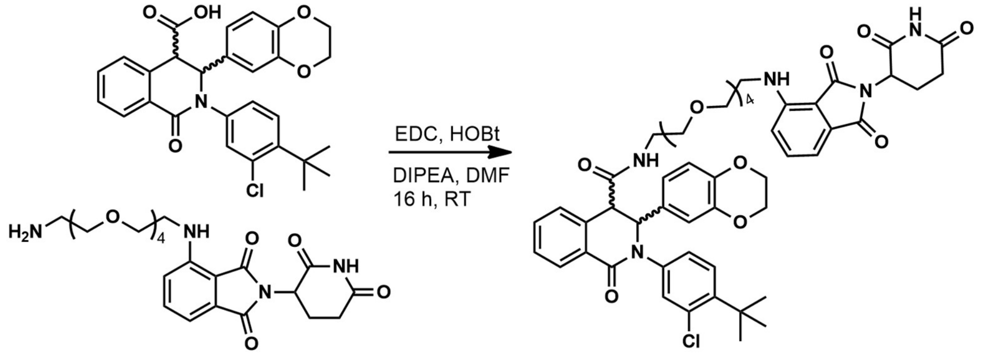

2-(4-(tert-butyl)-3-chlorophenyl)-3-(2,3-dihydrobenzo[b][1,4]dioxin-6-yl)-N-(14-((2-(2,6-dioxopiperidin-3-yl)-1,3-dioxoisoindolin-4-yl)amino)-3,6,9,12-tetraoxatetradecyl)-1-oxo-1,2,3,4-tetrahydroisoquinoline-4-carboxamide (BH729) was synthesized in accordance with General Procedure 2, employing 4-((14-amino-3,6,9,12-tetraoxatetradecyl)amino)-2-(2,6-dioxopiperidin-3-yl)isoindoline-1,3-dione (25 mg, 0.051 mmol), 2-(4-(tert-butyl)-3-chlorophenyl)-3-(2,3-dihydrobenzo[b][1,4]dioxin-6-yl)-1-oxo-1,2,3,4-tetrahydroisoquinoline-4-carboxylic acid (25 mg, 0.051 mmol), EDC (12 mg, 0.061 mmol), HOBt (13 mg, 0.066 mmol), and DIPEA (16 mg, 0.12 mmol). The product was obtained in 35% yield (17 mg) as a yellow amorphous solid. ^{1}H NMR (400 MHz, Acetonitrile-d_3) δ 8.97 (s, 1H), 8.05 (dd, *J* = 7.6, 1.7 Hz, 1H), 7.54 – 7.42 (m, 4H), 7.36 (d, *J* = 2.4 Hz, 1H), 7.20 (ddd, *J* = 8.6, 6.4, 1.9 Hz, 2H), 7.01 (d, *J* = 7.8 Hz, 2H), 6.69 – 6.58 (m, 3H), 6.46 (t, *J* = 5.7 Hz, 1H), 6.37 (s, 1H), 5.55 (d, *J* = 1.9 Hz, 1H), 4.94 – 4.86 (m, 1H), 4.11 (s, 4H), 3.95 (d, *J* = 1.9 Hz, 1H), 3.62 (t, *J* = 5.3 Hz, 2H), 3.57 – 3.35 (m, 14H), 3.27 (dd, *J* = 6.3, 5.0 Hz, 1H), 2.75 – 2.59 (m, 2H), 2.09 – 2.01 (m, 1H), 1.92 (p, *J* = 2.5 Hz, 3H), 1.42 (s, 9H). ^{13}C NMR (151 MHz, CD_3CN) δ 172.0, 170.0, 169.6, 169.5, 167.6, 163.1, 146.9, 144.7, 143.7, 143.2, 141.5, 136.1, 134.1, 132.9, 132.8, 132.6, 130.0, 129.5, 128.8, 128.6, 128.2, 128.2, 124.8, 119.1, 115.0, 110.8, 110.0, 70.2, 70.2 (2C), 70.1, 70.1, 70.0, 70.0, 70.0, 69.1, 69.0, 68.9, 64.5, 64.3, 64.2, 52.6, 48.9, 42.0, 39.4, 35.6, 31.1, 28.8 (3C), 22.3. HRMS (ESI, m/z) for $C_{51}H_{56}ClN_5O_{12}$ [M + Na]: expected, 988.3517; found, 988.3558; 4.1 ppm.

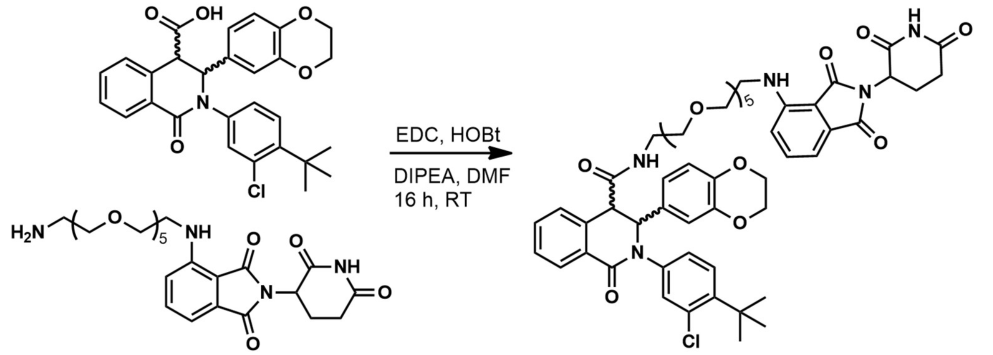

2-(4-(tert-butyl)-3-chlorophenyl)-3-(2,3-dihydrobenzo[b][1,4]dioxin-6-yl)-N-(17-((2-(2,6-dioxopiperidin-3-yl)-1,3-dioxoisoindolin-4-yl)amino)-3,6,9,12,15-pentaoxaheptadecyl)-1-oxo-1,2,3,4-tetrahydroisoquinoline-4-carboxamide (BH731) was synthesized in accordance with General Procedure 2, employing 4-((17-amino-3,6,9,12,15-pentaoxaheptadecyl)amino)-2-(2,6-dioxopiperidin-3-yl)isoindoline-1,3-dione (27 mg, 0.051 mmol), 2-(4-(tert-butyl)-3-chlorophenyl)-3-(2,3-dihydrobenzo[b][1,4]dioxin-6-yl)-1-oxo-1,2,3,4-tetrahydroisoquinoline-4-carboxylic acid (25 mg, 0.051 mmol), EDC (12 mg, 0.061 mmol), HOBt (13 mg, 0.066 mmol), and DIPEA (16 mg, 0.12 mmol). The product was obtained in 39% yield (20 mg) as a yellow

amorphous solid. ^{1}H NMR (400 MHz, Acetonitrile-d_3) δ 8.96 (s, 1H), 8.05 (dd, *J* = 7.5, 1.7 Hz, 1H), 7.54 – 7.43 (m, 4H), 7.36 (d, *J* = 2.3 Hz, 1H), 7.23 – 7.17 (m, 2H), 7.04 – 6.98 (m, 2H), 6.69 – 6.58 (m, 3H), 6.46 (t, *J* = 5.6 Hz, 1H), 6.38 (s, 1H), 5.55 (d, *J* = 1.9 Hz, 1H), 4.95 – 4.87 (m, 1H), 4.11 (s, 4H), 3.95 (d, *J* = 1.9 Hz, 1H), 2.79 – 2.57 (m, 3H), 2.09 – 2.02 (m, 1H), 1.92 (h, *J* = 2.8 Hz, 5H), 1.42 (s, 9H). ^{13}C NMR (151 MHz, CD_3CN) δ 172.0, 170.0, 169.6, 169.5, 167.6, 163.1, 146.9, 144.6, 143.7, 143.2, 141.5, 136.1, 134.1, 132.9, 132.8, 132.6, 132.6, 130.0, 129.5, 128.8, 128.6, 128.2, 128.2, 124.8, 119.1, 115.0, 110.8, 110.0, 70.2, 70.2, 70.2 (2 C), 70.1, 70.1, 70.1, 70.0, 70.0, 69.1, 69.0, 64.5, 64.3, 64.2, 52.6, 48.9, 42.0, 39.4, 35.6, 29.0, 28.8 (3 C), 22.3. HRMS (ESI, m/z) for $C_{53}H_{60}ClN_5O_{13}$ [M + Na]: expected, 1032.3779; found, 1032.3757; -2.1 ppm.

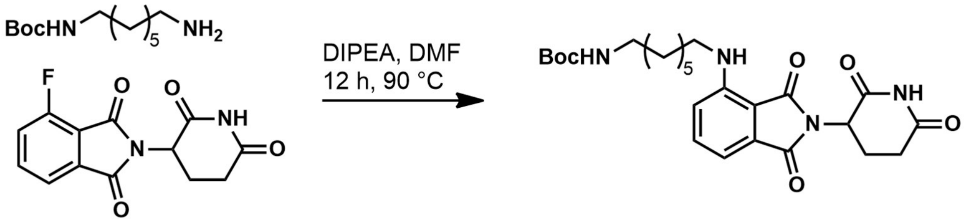

2-(2,6-dioxopiperidin-3-yl)-4-((8-((2,2,2-trifluoroacetyl)-l4-azaneyl)octyl)amino)isoindoline-1,3-dione. DIPEA (397 mg, 3.07 mmol), N-Boc-1,8-diaminooctane (500 mg, 2.05 mmol), and 2-(2,6-dioxopiperidin-3-yl)-4-fluoroisoindoline-1,3-dione (565 mg, 2.05 mmol) were stirred in 5 mL DMF at 90 °C for 12 h. The reaction was quenched with 25 mL water, extracted with EtOAc. The organics were collected, dried over anhydrous sodium sulfate, filtered, and concentrated under reduced pressure by rotary evaporation. The crude residue was purified by column chromatography (SiO_2, eluted with a gradient of 50-100% EtOAc in hexanes followed by 0-50% acetone in EtOAc). The product was obtained in 30% yield (307 mg) as a yellow amorphous solid. ^{1}H NMR (400 MHz, Chloroform-*d*) δ 8.05 (d, *J* = 18.2 Hz, 1H), 7.49 (dd, *J* = 8.6, 7.1 Hz, 1H), 7.09 (d, *J* = 7.0 Hz, 1H), 6.88 (d, *J* = 8.5 Hz, 1H), 6.23 (s, 1H), 4.92 (dd, *J* = 12.1, 5.3 Hz, 1H), 4.53 (s, 1H), 3.26 (s, 2H), 3.10 (d, *J* = 6.8 Hz, 2H), 2.97 – 2.67 (m, 4H), 2.23 – 2.09 (m, 1H), 1.65 (p, *J* = 7.1 Hz, 3H), 1.44 (s, 9H), 1.35 – 1.28 (m, 5H). To the isolated intermediate, 4 mL of a 3:1 v/v mixture of DCM:TFA was added and stirred for 3 h at 35 °C. The solvent was then evaporated under a stream of N_2 and the crude TFA salt used without further purification.

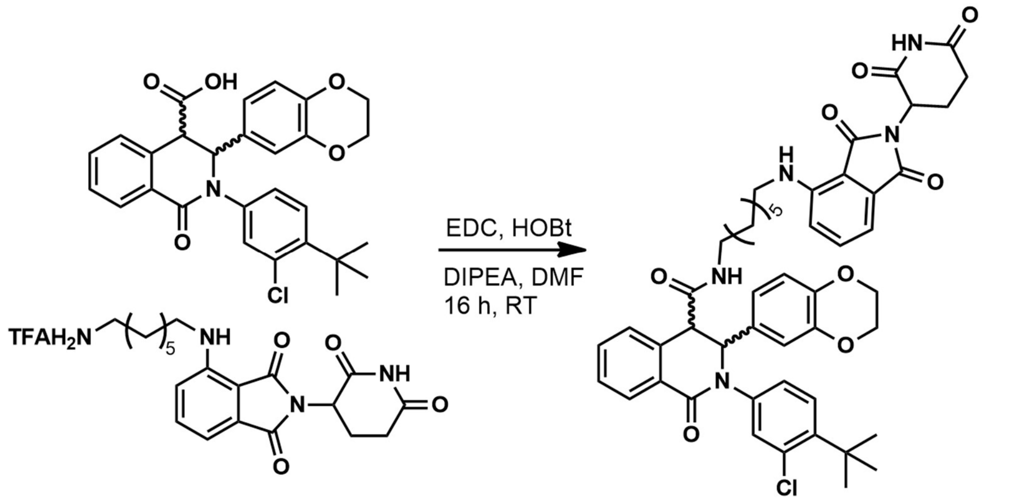

2-(4-(tert-butyl)-3-chlorophenyl)-3-(2,3-dihydrobenzo[b][1,4]dioxin-6-yl)-N-(8-((2-(2,6-dioxopiperidin-3-yl)-1,3-dioxoisoindolin-4-yl)amino)octyl)-1-oxo-1,2,3,4-tetrahydroisoquinoline-4-carboxamide (BH740) was synthesized in accordance with General Procedure 2, employing 2-(2,6-dioxopiperidin-3-yl)-4-((8-((2,2,2-trifluoroacetyl)-l4-azaneyl)octyl)amino)isoindoline-1,3-dione (25 mg, 0.051 mmol), 2-(4-(tert-butyl)-3-chlorophenyl)-3-(2,3-dihydrobenzo[b][1,4]dioxin-6-yl)-1-oxo-1,2,3,4-tetrahydroisoquinoline-4-carboxylic acid (25 mg, 0.051 mmol), EDC (12 mg, 0.061 mmol), HOBt (13 mg, 0.066 mmol), and DIPEA (16 mg, 0.12 mmol). The product was obtained in 38% yield (17 mg) as a yellow amorphous solid. ^{1}H NMR (400 MHz, Acetonitrile-d_3) δ 8.94 (s, 1H), 8.07 (dd, *J* = 7.4, 1.7 Hz, 1H), 7.56 – 7.42 (m, 4H), 7.39 (d, *J* = 2.3 Hz, 1H), 7.21 (dt, *J* = 8.5, 1.9 Hz, 2H), 7.00 (dd, *J* = 7.8, 6.0 Hz, 2H), 6.71 – 6.60 (m, 3H), 6.30 (t, *J* = 5.8 Hz, 1H), 6.22 (t, *J* = 5.8 Hz, 1H), 5.59 (d, *J* = 1.8 Hz, 1H), 4.92 (dd, *J* = 12.3, 5.0 Hz, 1H), 4.13 (s, 4H), 3.95 (d, *J* = 1.9 Hz, 1H), 3.23 (ddd, *J* = 12.0, 6.9, 5.3 Hz, 3H), 3.03 (dd, *J* = 13.1, 6.4 Hz, 1H), 2.82 – 2.61 (m, 3H), 2.08 (ddd, *J* = 10.2, 5.3, 3.0 Hz, 1H), 1.56 (q, *J* = 7.2 Hz, 2H), 1.42 (s, 9H), 1.40 – 1.10 (m, 7H). ^{13}C NMR (151 MHz, CD_3CN) δ 172.0, 169.8, 169.6, 169.5, 167.7, 163.1, 147.0, 144.6, 143.7, 143.2, 141.5, 136.2, 134.1, 132.9, 132.9, 132.6, 132.6, 130.0, 129.5, 128.9, 128.6, 128.2, 128.2, 124.8, 119.1, 117.0, 115.0, 110.5, 109.6, 64.8, 64.8, 64.3, 64.2, 52.9, 52.8, 52.8, 48.9, 42.2, 39.3, 35.6, 31.1, 29.1, 28.8, 28.8 (2C), 28.8, 26.4, 26.2, 22.3. HRMS (ESI, m/z) for $C_{49}H_{52}ClN_5O_8$ [M + H]: expected, 874.3577; found, 874.3609; 3.7 ppm.

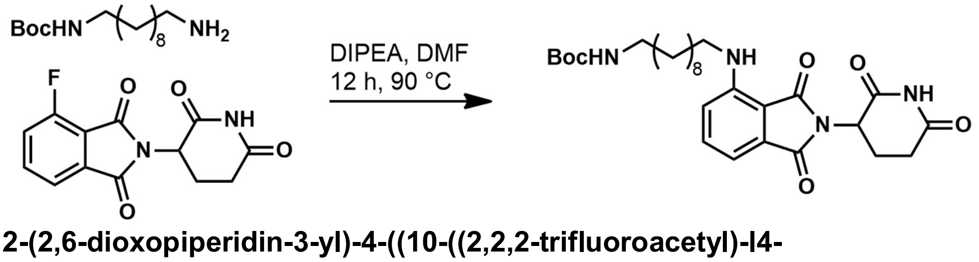

2-(2,6-dioxopiperidin-3-yl)-4-((10-((2,2,2-trifluoroacetyl)-l4-azaneyl)decyl)amino)isoindoline-1,3-dione. DIPEA (397 mg, 3.07 mmol), N-Boc-decane-1,10-diamine (500 mg, 1.84 mmol), and 2-(2,6-dioxopiperidin-3-yl)-4-fluoroisoindoline-1,3-dione (507 mg, 1.84 mmol) were stirred in 5 mL DMF at 90 °C for 12 h. The reaction was quenched with 25 mL water, extracted with EtOAc. The organics were collected, dried over anhydrous sodium sulfate, filtered, and concentrated under reduced pressure by rotary evaporation. The

crude residue was purified by column chromatography (SiO_2, eluted with a gradient of 50-100% EtOAc in hexanes followed by 0-50% acetone in EtOAc). The product was obtained in 35% yield (335 mg) as a yellow amorphous solid. ^1H NMR (400 MHz, Chloroform-*d*) δ 8.15 (s, 1H), 7.49 (dd, *J* = 8.5, 7.1 Hz, 1H), 7.08 (d, *J* = 7.0 Hz, 1H), 6.88 (d, *J* = 8.6 Hz, 1H), 6.22 (s, 1H), 4.91 (dd, *J* = 12.1, 5.3 Hz, 1H), 4.52 (s, 1H), 3.25 (t, *J* = 7.0 Hz, 3H), 3.16 – 3.04 (m, 3H), 2.95 – 2.68 (m, 4H), 2.20 – 2.08 (m, 1H), 1.64 (q, *J* = 7.3 Hz, 2H), 1.44 (s, 16H), 1.29 (d, *J* = 5.1 Hz, 11H). To the isolated intermediate, 4 mL of a 3:1 v/v mixture of DCM:TFA was added and stirred for 3 h at 35 °C. The solvent was then evaporated under a stream of N_2 and the crude TFA salt used without further purification.

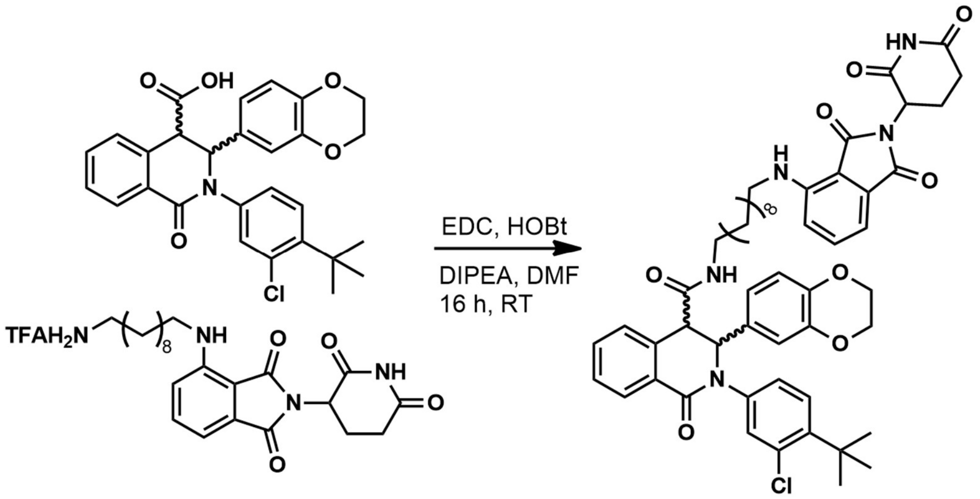

2-(4-(tert-butyl)-3-chlorophenyl)-3-(2,3-dihydrobenzo[b][1,4]dioxin-6-yl)-N-(10-((2-(2,6-dioxopiperidin-3-yl)-1,3-dioxoisoindolin-4-yl)amino)decyl)-1-oxo-1,2,3,4-tetrahydroisoquinoline-4-carboxamide (BH741) was synthesized in accordance with General Procedure 2, employing 2-(2,6-dioxopiperidin-3-yl)-4-((10-((2,2,2-trifluoroacetyl)-l4-azaneyl)decyl)amino)isoindoline-1,3-dione (128 mg, 0.244 mmol), 2-(4-(tert-butyl)-3-chlorophenyl)-3-(2,3-dihydrobenzo[b][1,4]dioxin-6-yl)-1-oxo-1,2,3,4-tetrahydroisoquinoline-4-carboxylic acid (120 mg, 0.244 mmol), EDC (56 mg, 0.293 mmol), HOBt (61 mg, 0.317 mmol), and DIPEA (76 mg, 0.585 mmol). The product was obtained in 75% yield (165 mg) as a yellow amorphous solid. ^1H NMR (400 MHz, Acetonitrile-d_3) δ 8.95 (s, 1H), 8.09 (dd, *J* = 7.5, 1.8 Hz, 1H), 7.58 – 7.45 (m, 4H), 7.40 (d, *J* = 2.3 Hz, 1H), 7.26 – 7.20 (m, 2H), 7.06 – 7.01 (m, 2H), 6.71 (d, *J* = 8.3 Hz, 1H), 6.69 – 6.62 (m, 2H), 6.36 – 6.21 (m, 2H), 5.60 (d, *J* = 1.9 Hz, 1H), 4.95 (dd, *J* = 12.0, 5.3 Hz, 1H), 4.16 (s, 4H), 3.97 (d, *J* = 1.9 Hz, 1H), 3.26 (dq, *J* = 20.7, 6.6 Hz, 3H), 3.06 (dd, *J* = 13.0, 6.4 Hz, 1H), 2.83 – 2.60 (m, 3H), 1.61 (q, *J* = 7.3 Hz, 2H), 1.45 (s, 8H), 1.42 – 1.26 (m, 3H), 1.20 (s, 8H).^{13}C NMR (151 MHz, CD_3CN) δ 172.0, 169.8, 169.5, 167.7, 163.1, 147.0, 144.6, 143.7, 143.2, 141.5, 136.2, 134.1, 132.9, 132.9, 132.6, 132.6, 130.0, 129.5, 128.9, 128.5, 128.4, 128.2, 128.0, 124.8, 119.1, 117.0, 115.0, 110.5, 109.7, 64.8, 64.3, 64.2, 52.8, 48.9, 42.2, 42.1, 39.3, 35.6, 31.1, 31.0, 29.1, 29.1 (3C), 29.0, 28.9, 28.9, 28.8, 26.5, 26.3, 22.3. HRMS (ESI, m/z) for $C_{51}H_{56}ClN_5O_8$ [M + H]: expected, 902.3890; found, 902.3915; 2.8 ppm. The mixture of trans enantiomers was further purified by chiral HPLC (Phenomenex Lux Cellulose-1 column, 100% MeCN) to afford the separate isomers.

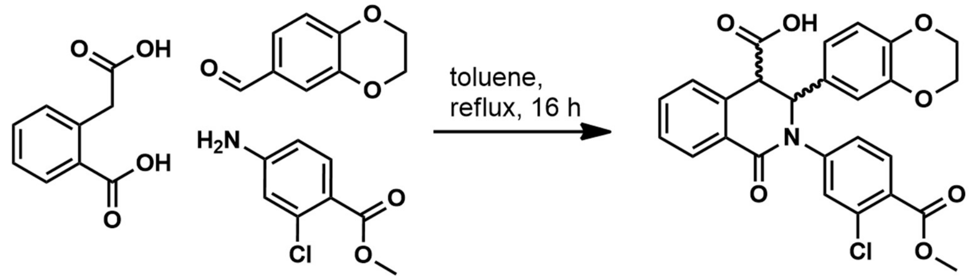

2-(3-chloro-4-(methoxycarbonyl)phenyl)-3-(2,3-dihydrobenzo[b][1,4]dioxin-6-yl)-1-oxo-1,2,3,4-tetrahydroisoquinoline-4-carboxylic acid was synthesized in accordance with General Procedure 1, employing homophthalic acid (970 mg, 5.39 mmol), methyl 4-amino-2-chlorobenzoate (1000 mg, 5.39 mmol), 2,3-dihydro-1,4-benzodioxin-6-carbaldehyde (884 mg, 5.39 mmol), in toluene (40 mL). The reaction was quenched with deionized water, extracted with EtOAc, and washed with 1 M aq. HCl and brine. The organic phase was dried over anhydrous anhydrous sodium sulfate, filtered, and concentrated under reduced pressure by rotary evaporation. The crude residue was purified by flash column chromatography (SiO_2, eluted with a gradient of 0-10% MeOH in DCM). The impure product was used without further purification.

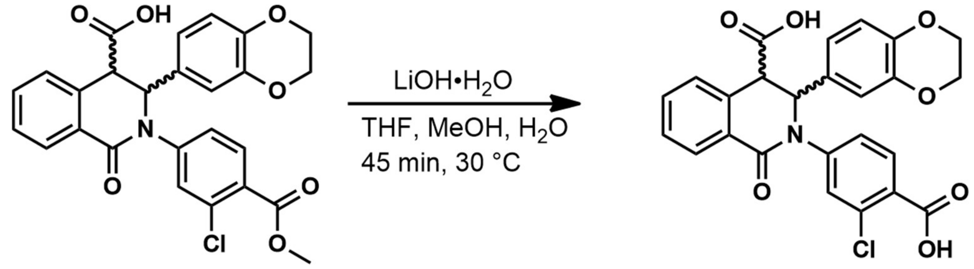

2-(4-carboxy-3-chlorophenyl)-3-(2,3-dihydrobenzo[b][1,4]dioxin-6-yl)-1-oxo-1,2,3,4-tetrahydroisoquinoline-4-carboxylic acid. Lithium hydroxide monohydrate (1 g, 20 mmol) was added to a stirred solution of 2-(4-carboxy-3-chlorophenyl)-3-(2,3-dihydrobenzo[b][1,4]dioxin-6-yl)-1-oxo-1,2,3,4-tetrahydroisoquinoline-4-carboxylic acid (1.5 g, 5.4 mmol) in a 5 mL mixture of THF/MeOH/H_2O at a 3:1:1 (v/v/v) ratio. The reaction was stirred for 2 hr at 35 °C, quenched with 1 M aq. HCl, extracted with EtOAc, washed with 1 M aq. HCl and brine, dried over anhydrous sodium sulfate, and filtered. The organic phase was concentrated under reduced pressure by rotary evaporation. The crude residue was purified by column chromatography (SiO_2, eluted with a gradient of 40-80% EtOAc in hexanes). The product was obtained in 9.2% yield (238 mg) over two steps as a white amorphous solid.

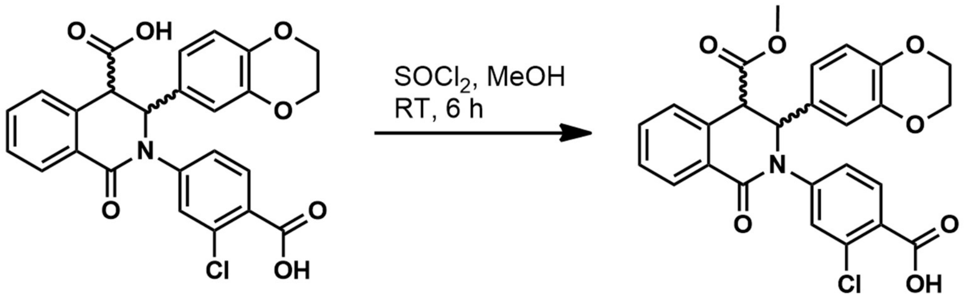

2-chloro-4-(3-(2,3-dihydrobenzo[b][1,4]dioxin-6-yl)-4-(methoxycarbonyl)-1-oxo-3,4-dihydroisoquinolin-2(1H)-yl)benzoic acid. Thionyl chloride (21.6 mg, 0.182 mmol) was added dropwise to a stirred solution of 2-(4-carboxy-3-chlorophenyl)-3-(2,3-dihydrobenzo[b][1,4]dioxin-6-yl)-1-oxo-1,2,3,4-tetrahydroisoquinoline-4-carboxylic acid in (415 mg, 0.865 mmol) in MeOH (10 mL) under a nitrogen atmosphere. The reaction was stirred for 16 h at room temperature, quenched with 1 M aq. HCl, extracted with EtOAc, washed with 1 M aq. HCl and brine, dried over anhydrous sodium sulfate, and filtered. The organic phase was concentrated under reduced pressure by rotary evaporation. The crude residue was purified by flash column chromatography (SiO_2, eluted with a gradient of 25-55% EtOAc in hexanes). The product was obtained in 2.1% yield over 3 steps as a white amorphous solid. ^{1}H NMR (400 MHz, DMSO-d_6) δ 8.01 (dd, J = 7.6, 1.4 Hz, 1H), 7.84 (d, J = 8.4 Hz, 1H), 7.58 – 7.52 (m, 2H), 7.48 (td, J = 7.5, 1.3 Hz, 1H), 7.41 – 7.33 (m, 2H), 6.76 – 6.69 (m, 2H), 6.62 (dd, J = 8.5, 2.3 Hz, 1H), 5.77 – 5.68 (m, 1H), 4.39 (d, J = 1.8 Hz, 1H), 4.15 (s, 4H), 1.91 (s, 3H).

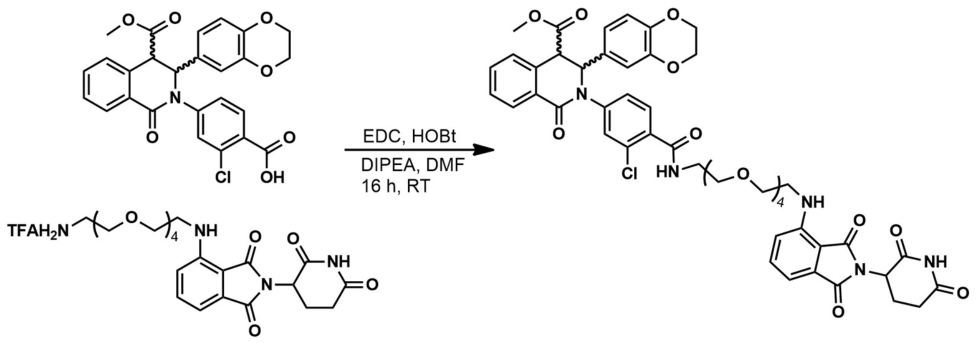

methyl 2-(3-chloro-4-((14-((2-(2,6-dioxopiperidin-3-yl)-1,3-dioxoisoindolin-4-yl)amino)-3,6,9,12-tetraoxatetradecyl)carbamoyl)phenyl)-3-(2,3-dihydrobenzo[b][1,4]dioxin-6-yl)-1-oxo-1,2,3,4-tetrahydroisoquinoline-4-carboxylate (BH726) was synthesized in accordance with General Procedure 2, employing 2-chloro-4-(3-(2,3-dihydrobenzo[b][1,4]dioxin-6-yl)-4-(methoxycarbonyl)-1-oxo-3,4-dihydroisoquinolin-2(1H)-yl)benzoic acid (25 mg, 0.051 mmol), 2-(2,6-dioxopiperidin-3-yl)-4-((1,1,1-trifluoro-2-oxo-6,9,12,15-tetraoxa-3l4-azaheptadecan-17-yl)amino)isoindoline-1,3-dione (27 mg, 0.051 mmol), EDC (13 mg, 0.066 mmol), HOBt (12 mg, 0.061 mmol), and DIPEA (20 mg, 0.150 mmol). The product was obtained in 90% yield (44 mg) as a yellow amorphous solid. ^{1}H NMR (400 MHz, Acetonitrile-d_3) δ 8.98 (s, 1H), 8.07 (dd, J = 7.6, 1.6 Hz, 1H), 7.53 (dd, J = 8.5, 7.0 Hz, 2H), 7.51 – 7.44 (m, 3H), 7.34 (dd, J = 8.3, 2.1 Hz, 1H), 7.29 (dd, J = 7.4, 1.4 Hz, 1H), 7.07 – 6.98 (m, 3H), 6.69 (d, J = 8.2 Hz, 1H), 6.67 – 6.60 (m, 2H), 6.48 (t, J = 5.7 Hz, 1H), 5.63 (d, J = 1.7 Hz, 1H), 4.92 (dd, J = 12.3, 5.0 Hz, 1H), 4.21 (d, J = 1.8 Hz, 1H), 4.14 (s, 4H), 3.68 (s, 4H), 3.66 (t, J = 5.6 Hz, 2H), 3.60 – 3.52 (m, 7H), 3.48 (ddt, J = 10.7, 5.8, 2.8 Hz, 10H), 2.81 – 2.58 (m, 3H), 2.12 – 2.05 (m, 1H). ^{13}C NMR (214 MHz, CD_3CN) δ 172.0, 171.0, 169.5, 169.5, 167.7, 166.1, 163.0, 146.9, 144.4, 143.8, 143.4, 136.1, 134.6, 133.3, 132.9, 132.6, 131.7, 130.4, 129.7, 129.4, 129.2, 128.6, 127.9, 127.7, 124.9, 119.2, 117.3, 117.2, 115.1, 110.8, 109.9, 70.2, 70.2, 70.2, 70.1, 70.1 (2C), 70.0, 69.1, 69.0, 64.3, 64.2,

63.5, 52.5, 51.0, 48.9, 42.0, 39.5, 31.1, 22.3. HRMS (ESI, m/z) for $C_{49}H_{50}ClN_5O_{14}$ [M + Na]: expected, 990.2935; found, 990.2931; -0.4 ppm.

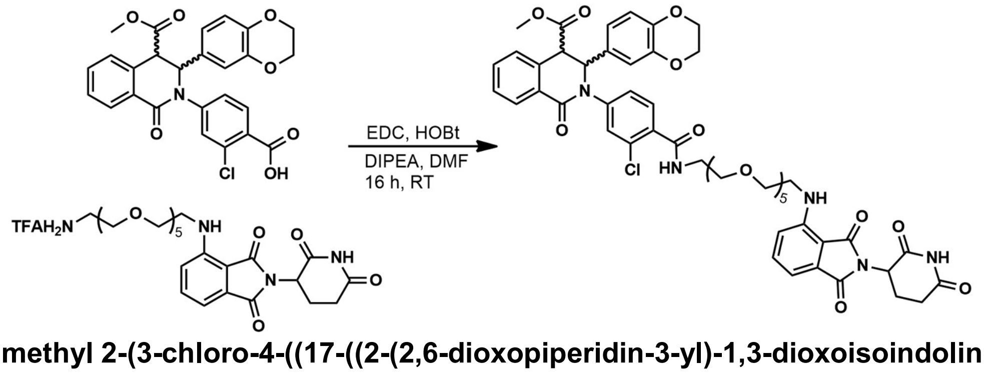

methyl 2-(3-chloro-4-((17-((2-(2,6-dioxopiperidin-3-yl)-1,3-dioxoisoindolin-4-yl)amino)-3,6,9,12,15-pentaoxaheptadecyl)carbamoyl)phenyl)-3-(2,3-dihydrobenzo[b][1,4]dioxin-6-yl)-1-oxo-1,2,3,4-tetrahydroisoquinoline-4-carboxylate (BH727) was synthesized in accordance with General Procedure 2, employing 2-chloro-4-(3-(2,3-dihydrobenzo[b][1,4]dioxin-6-yl)-4-(methoxycarbonyl)-1-oxo-3,4-dihydroisoquinolin-2(1H)-yl)benzoic acid (25 mg, 0.051 mmol), 2-(2,6-dioxopiperidin-3-yl)-4-((1,1,1-trifluoro-2-oxo-6,9,12,15,18-pentaoxa-3l4-azaicosan-20-yl)amino)isoindoline-1,3-dione (32 mg, 0.051 mmol), EDC (13 mg, 0.066 mmol), HOBt (12 mg, 0.061 mmol), and DIPEA (20 mg, 0.150 mmol). The product was obtained in 94% yield (48 mg) as a yellow amorphous solid. ^{1}H NMR (400 MHz, Acetonitrile-d_3) δ 8.99 (s, 1H), 8.10 (dd, J = 7.6, 1.6 Hz, 1H), 7.60 – 7.45 (m, 5H), 7.37 (dd, J = 8.3, 2.0 Hz, 1H), 7.32 (dd, J = 7.5, 1.4 Hz, 1H), 7.07 (d, J = 3.6 Hz, 1H), 7.06 – 7.01 (m, 2H), 6.72 (d, J = 8.3 Hz, 1H), 6.69 – 6.62 (m, 2H), 6.50 (t, J = 5.7 Hz, 1H), 5.66 (d, J = 1.8 Hz, 1H), 4.95 (dd, J = 12.3, 5.0 Hz, 1H), 4.23 (d, J = 1.8 Hz, 1H), 4.17 (s, 4H), 3.71 (s, 3H), 3.67 (t, J = 5.3 Hz, 2H), 3.64 – 3.43 (m, 19H), 2.84 – 2.62 (m, 4H), 2.10 (dtd, J = 10.1, 4.9, 3.1 Hz, 2H). ^{13}C NMR (214 MHz, CD_3CN) δ 172.0, 171.0, 169.5, 169.5, 167.7, 166.1, 163.0, 146.9, 144.4, 143.8, 143.4, 136.1, 134.6, 133.3, 132.9, 132.6, 131.7, 130.4, 129.7, 129.4, 129.2, 128.6, 127.9, 127.7, 124.9, 119.2, 117.2, 115.1, 110.8, 109.9, 70.2, 70.2, 70.2, 70.1 (2C), 70.1, 70.0, 69.1, 69.0, 64.3, 64.2, 63.5, 52.5, 51.0, 48.9, 42.0, 41.9, 39.5, 31.1, 31.0, 22.3. HRMS (ESI, m/z) for $C_{51}H_{54}ClN_5O_{15}$ [M + Na]: expected, 1034.3197; found, 1034.3192; -0.5 ppm.

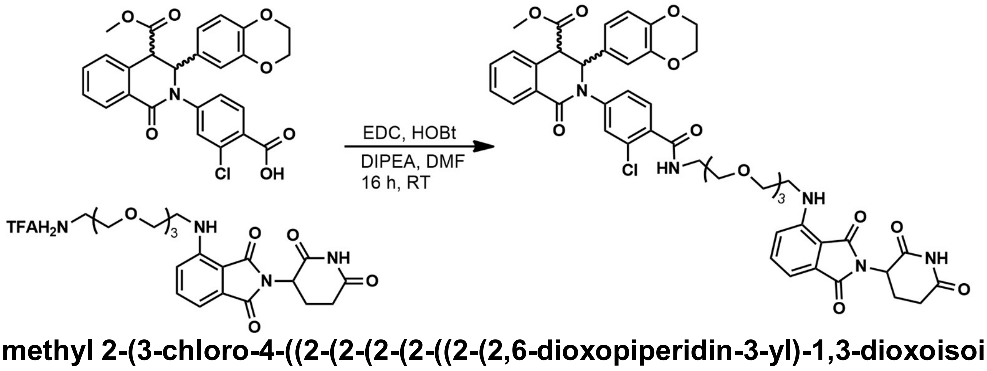

methyl 2-(3-chloro-4-((2-(2-(2-(2-((2-(2,6-dioxopiperidin-3-yl)-1,3-dioxoisoindolin-4-yl)amino)ethoxy)ethoxy)ethoxy)ethyl)carbamoyl)phenyl)-3-(2,3-dihydrobenzo[b][1,4]dioxin-6-yl)-1-oxo-1,2,3,4-tetrahydroisoquinoline-4-carboxylate (BH743) was synthesized in accordance with General Procedure 2, employing 2-chloro-4-(3-(2,3-dihydrobenzo[b][1,4]dioxin-6-yl)-4-(methoxycarbonyl)-1-oxo-3,4-dihydroisoquinolin-2(1H)-

yl)benzoic acid (25 mg, 0.051 mmol), 2-(2,6-dioxopiperidin-3-yl)-4-((1,1,1-trifluoro-2-oxo-6,9,12-trioxa-3l4-azatetradecan-14-yl)amino)isoindoline-1,3-dione (28 mg, 0.051 mmol), EDC (13 mg, 0.066 mmol), HOBt (12 mg, 0.061 mmol), and DIPEA (16 mg, 0.120 mmol). The product was obtained in 36% yield (17 mg) as a yellow amorphous solid. ^{1}H NMR (400 MHz, Acetonitrile-d_3) δ 8.98 (s, 1H), 8.09 (dd, J = 7.5, 1.6 Hz, 1H), 7.81 (d, J = 8.5 Hz, 1H), 7.58 – 7.45 (m, 4H), 7.37 (dt, J = 8.5, 1.8 Hz, 1H), 7.25 (d, J = 7.9 Hz, 1H), 7.02 (dd, J = 7.8, 3.5 Hz, 2H), 6.71 – 6.58 (m, 3H), 6.50 – 6.38 (m, 2H), 5.63 (d, J = 2.0 Hz, 1H), 4.92 (dd, J = 12.1, 5.4 Hz, 1H), 4.14 (s, 4H), 4.02 (d, J = 2.1 Hz, 1H), 3.85 (s, 3H), 3.64 (t, J = 5.3 Hz, 2H), 3.59 – 3.49 (m, 4H), 3.47 – 3.35 (m, 8H), 3.31 – 3.19 (m, 2H), 2.81 – 2.58 (m, 3H), 2.11 – 2.04 (m, 1H). ^{13}C NMR (101 MHz, CD_3CN) δ 171.7, 169.5, 169.2, 169.2, 169.1, 167.3, 164.8, 162.8, 146.5, 145.9, 143.4, 142.9, 135.8, 133.9, 132.9, 132.6, 132.2, 131.9, 131.9, 131.4, 129.3, 128.4, 128.3, 128.0, 128.0, 127.2, 124.2, 118.7, 114.7, 110.5, 69.9, 69.8, 69.8, 69.6, 68.7, 68.5, 64.0, 64.0, 63.9, 63.9, 52.1, 51.7, 48.6, 41.7, 39.0, 30.7, 22.0. HRMS (ESI, m/z) for $C_{47}H_{46}ClN_5O_{13}$ [M + H]: expected, 924.2853; found, 924.2874; 2.3 ppm.

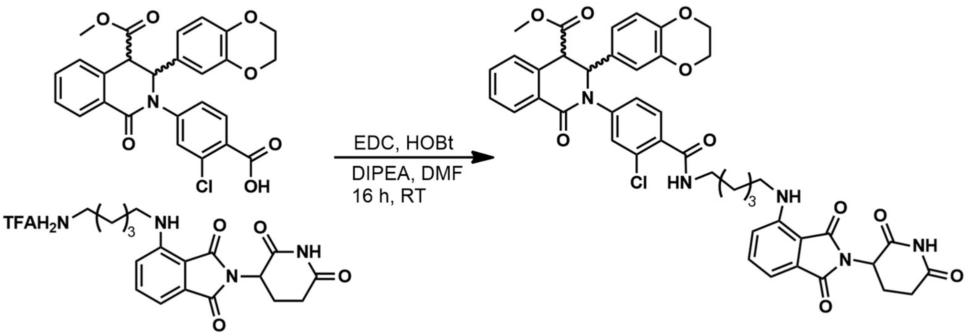

methyl 2-(3-chloro-4-((8-((2-(2,6-dioxopiperidin-3-yl)-1,3-dioxoisoindolin-4-yl)amino)octyl)carbamoyl)phenyl)-3-(2,3-dihydrobenzo[b][1,4]dioxin-6-yl)-1-oxo-1,2,3,4-tetrahydroisoquinoline-4-carboxylate (BH753) was synthesized in accordance with General Procedure 2, employing 2-chloro-4-(3-(2,3-dihydrobenzo[b][1,4]dioxin-6-yl)-4-(methoxycarbonyl)-1-oxo-3,4-dihydroisoquinolin-2(1H)-yl)benzoic acid (28 mg, 0.057 mmol), 2-(2,6-dioxopiperidin-3-yl)-4-((8-((2,2,2-trifluoroacetyl)-l4-azaneyl)octyl)amino)isoindoline-1,3-dione (28 mg, 0.057 mmol), EDC (14 mg, 0.074 mmol), HOBt (13 mg, 0.068 mmol), and DIPEA (18 mg, 0.140 mmol). The product was obtained in 11% yield (5.7 mg) as a yellow amorphous solid. ^{1}H NMR (400 MHz, Acetonitrile-d_3) δ 8.92 (s, 1H), 8.08 (dt, J = 7.7, 1.8 Hz, 1H), 7.57 – 7.45 (m, 4H), 7.42 (dd, J = 8.2, 1.9 Hz, 1H), 7.37 – 7.27 (m, 2H), 7.05 – 6.98 (m, 2H), 6.83 (s, 1H), 6.70 (dd, J = 8.4, 1.9 Hz, 1H), 6.63 (ddd, J = 10.4, 5.2, 2.1 Hz, 2H), 6.32 (s, 1H), 5.63 (d, J = 2.0 Hz, 1H), 4.96 – 4.88 (m, 1H), 4.21 (d, J = 1.8 Hz, 1H), 4.15 (d, J = 1.9 Hz, 4H), 3.69 (d, J = 1.9 Hz, 3H), 3.35 – 3.24 (m, 4H), 2.82 – 2.59 (m, 3H), 2.12 – 2.05 (m, 2H), 1.69 – 1.49 (m, 4H), 1.37 (t, J = 7.4 Hz, 8H). ^{13}C NMR (214 MHz, CD_3CN) δ 172.0, 171.1, 169.6, 167.7, 166.0, 163.0, 147.0, 144.2, 143.8, 143.4, 136.2, 135.0, 133.3, 132.9, 132.6, 131.7, 130.3, 129.7, 129.2, 128.6, 127.9, 127.6, 124.9, 119.2, 117.3, 117.0, 115.1, 110.5, 109.7, 64.3, 64.2, 63.5, 52.5, 51.0, 48.9, 48.9, 42.2, 42.0, 39.3, 31.1, 29.0, 28.9, 28.8, 28.8, 26.4, 26.4, 22.3. HRMS (ESI, m/z) for $C_{47}H_{46}ClN_5O_{10}$ [M + H]: expected, 876.3005; found, 876.3025; 2.2 ppm.

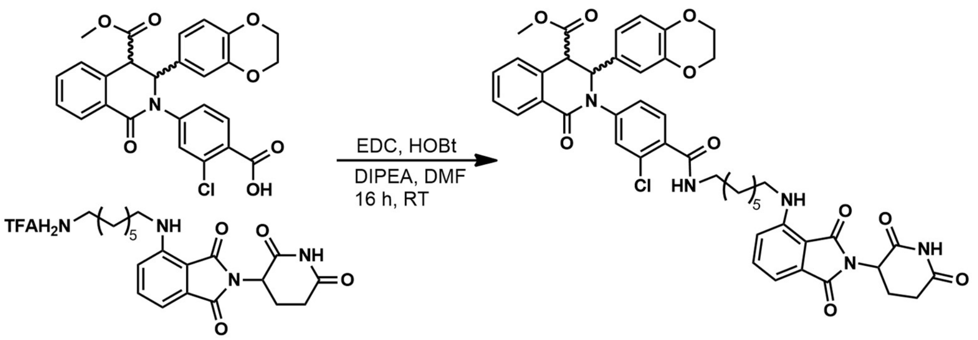

methyl 2-(3-chloro-4-((10-((2-(2,6-dioxopiperidin-3-yl)-1,3-dioxoisoindolin-4-yl)amino)decyl)carbamoyl)phenyl)-3-(2,3-dihydrobenzo[b][1,4]dioxin-6-yl)-1-oxo-1,2,3,4-tetrahydroisoquinoline-4-carboxylate (BH754) was synthesized in accordance with General Procedure 2, employing 2-chloro-4-(3-(2,3-dihydrobenzo[b][1,4]dioxin-6-yl)-4-(methoxycarbonyl)-1-oxo-3,4-dihydroisoquinolin-2(1H)-yl)benzoic acid (28 mg, 0.057 mmol), 2-(2,6-dioxopiperidin-3-yl)-4-((10-((2,2,2-trifluoroacetyl)-l4-azaneyl)decyl)amino)isoindoline-1,3-dione (30 mg, 0.057 mmol), EDC (14 mg, 0.074 mmol), HOBt (13 mg, 0.068 mmol), and DIPEA (18 mg, 0.140 mmol). The product was obtained in 6% yield (3 mg) as a yellow amorphous solid. ^{1}H NMR (400 MHz, Acetonitrile-*d*$_3$) δ 8.92 (s, 1H), 8.07 (dd, *J* = 7.6, 1.6 Hz, 1H), 7.57 – 7.45 (m, 4H), 7.42 (d, *J* = 8.2 Hz, 1H), 7.31 (ddd, *J* = 15.9, 7.8, 1.7 Hz, 2H), 7.04 – 6.99 (m, 2H), 6.82 (t, *J* = 5.9 Hz, 1H), 6.70 (d, *J* = 8.2 Hz, 1H), 6.66 – 6.59 (m, 2H), 6.31 (t, *J* = 5.8 Hz, 1H), 5.63 (d, *J* = 1.8 Hz, 1H), 4.93 (dd, *J* = 12.2, 5.3 Hz, 1H), 4.21 (d, *J* = 1.8 Hz, 1H), 4.14 (s, 4H), 3.68 (s, 3H), 3.29 (p, *J* = 6.4 Hz, 4H), 2.81 – 2.60 (m, 3H), 2.13 – 2.04 (m, 2H), 1.57 (dq, *J* = 36.7, 6.9 Hz, 4H), 1.33 (d, *J* = 18.2 Hz, 12H). ^{13}C NMR (214 MHz, CD_3CN) δ 172.0, 171.1, 169.6, 167.7, 166.0, 163.0, 147.0, 144.2, 143.8, 143.4, 136.1, 135.1, 133.3, 132.9, 132.6, 131.7, 130.3, 129.7, 129.2, 128.6, 127.9, 127.6, 124.9, 119.2, 117.0, 115.1, 110.5, 109.7, 64.3, 64.2, 63.5, 52.5, 51.0, 48.9, 48.9, 42.2, 42.1, 39.3, 31.1, 31.0, 29.1, 29.0, 28.9, 28.9, 28.8, 28.8, 26.5, 26.5, 22.3. HRMS (ESI, m/z) for $C_{49}H_{50}ClN_5O_{10}$ [M + H]: expected, 904.3319; found, 904.3343; 2.7 ppm.

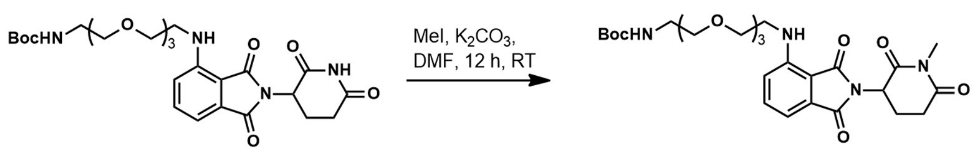

tert-butyl (2-(2-(2-(2-((2-(1-methyl-2,6-dioxopiperidin-3-yl)-1,3-dioxoisoindolin-4-yl)amino)ethoxy)ethoxy)ethoxy)ethyl)carbamate. To a stirred solution of tert-butyl (2-(2-(2-(2-((2-(2,6-dioxopiperidin-3-yl)-1,3-dioxoisoindolin-4-yl)amino)ethoxy)ethoxy)ethoxy)ethyl)carbamate (250 mg, 0.456 mmol) in DMF (5 mL), potassium carbonate (95 mg, 0.684 mmol) was added, then methyl iodide (78 mg, 0.547 mmol) was added dropwise. The reaction was stirred for 12 h at room temperature. The reaction was quenched with deionized water, extracted with EtOAc, and washed with 0.1 M aq. HCl, saturated sodium bicarbonate, and brine. The organic phase was dried over anhydrous sodium sulfate, filtered, and concentrated under reduced pressure by rotary evaporation. The crude residue was purified by flash column chromatography (SiO_2, eluted with a gradient of 50-100% EtOAc in hexanes, followed by 0-4% MeOH in EtOAc). The product was obtained in 84% yield (215 mg) as a yellow solid. ^{1}H NMR (400 MHz, Chloroform-*d*) δ 7.49 (dd, *J* = 8.5, 7.1 Hz, 1H), 7.10 (d, *J* = 7.1 Hz, 1H), 6.92 (d, *J* = 8.5 Hz, 1H), 6.48 (s, 1H), 5.01 (s, 1H), 4.95 – 4.88 (m, 1H),

3.72 (t, *J* = 5.5 Hz, 2H), 3.67 (s, 4H), 3.66 – 3.58 (m, 4H), 3.50 (dt, *J* = 20.6, 5.3 Hz, 4H), 3.30 (q, *J* = 5.5 Hz, 2H), 3.21 (s, 3H), 3.01 – 2.93 (m, 1H), 2.82 – 2.67 (m, 2H), 2.09 (td, *J* = 6.9, 4.8 Hz, 1H), 1.62 (d, *J* = 31.1 Hz, 1H), 1.43 (s, 9H). To the isolated intermediate, 4 mL of a 3:1 v/v mixture of DCM:TFA was added and stirred for 3 h at 35 °C. The solvent was then evaporated under a stream of N_2 and the crude TFA salt used without further purification.

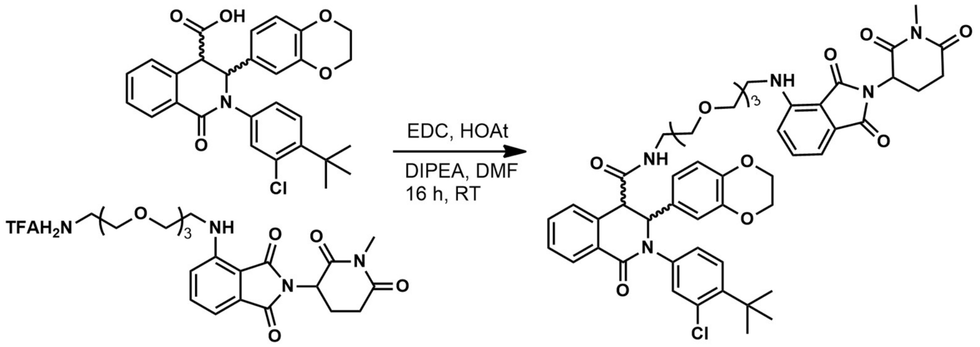

2-(4-(tert-butyl)-3-chlorophenyl)-3-(2,3-dihydrobenzo[b][1,4]dioxin-6-yl)-N-(2-(2-(2-(2-((2-(1-methyl-2,6-dioxopiperidin-3-yl)-1,3-dioxoisoindolin-4-yl)amino)ethoxy)ethoxy)ethoxy)ethyl)-1-oxo-1,2,3,4-tetrahydroisoquinoline-4-carboxamide (BH780) was synthesized in accordance with General Procedure 2, employing 2-chloro-4-(3-(2,3-dihydrobenzo[b][1,4]dioxin-6-yl)-4-(methoxycarbonyl)-1-oxo-3,4-dihydroisoquinolin-2(1H)-yl)benzoic acid (35 mg, 0.072 mmol), 2,2,2-trifluoro-N-(2-(2-(2-(2-((2-(1-methyl-2,6-dioxopiperidin-3-yl)-1,3-dioxoisoindolin-4-yl)amino)ethoxy)ethoxy)ethoxy)ethyl)acetamide (40 mg, 0.072 mmol), EDC (16 mg, 0.086 mmol), HOAt (13 mg, 0.093 mmol), and DIPEA (22 mg, 0.170 mmol). The product was obtained in 70% yield (47 mg) as a yellow amorphous solid. ^{1}H NMR (400 MHz, Chloroform-*d*) δ 8.27 (dd, *J* = 5.7, 3.5 Hz, 1H), 7.55 – 7.47 (m, 3H), 7.38 (dd, *J* = 5.4, 3.1 Hz, 2H), 7.18 (tt, *J* = 5.8, 3.4 Hz, 2H), 7.12 (d, *J* = 7.1 Hz, 1H), 6.92 (d, *J* = 8.5 Hz, 1H), 6.73 – 6.66 (m, 2H), 6.63 (dd, *J* = 8.5, 2.3 Hz, 1H), 5.83 – 5.79 (m, 1H), 5.77 (d, *J* = 5.8 Hz, 1H), 4.93 (td, *J* = 7.9, 3.3 Hz, 1H), 4.18 (s, 4H), 3.87 (s, 1H), 3.70 (t, *J* = 5.5 Hz, 2H), 3.66 – 3.57 (m, 4H), 3.48 (dtd, *J* = 13.1, 7.1, 6.6, 2.9 Hz, 8H), 3.43 – 3.33 (m, 2H), 3.21 (s, 3H), 3.05 – 2.92 (m, 1H), 2.84 – 2.71 (m, 2H), 2.10 (q, *J* = 7.4, 6.6 Hz, 1H), 2.03 (s, 3H), 1.45 (s, 9H). ^{13}C NMR (214 MHz, $CDCl_3$) δ 171.3, 169.9, 169.5, 169.0, 167.8, 162.9, 146.8, 145.0, 143.6, 143.1, 140.4, 136.0, 133.6, 133.1, 133.0, 132.6, 132.2, 129.9, 129.6, 129.2, 129.1, 129.0, 128.0, 124.3, 119.1, 117.5, 116.7, 115.2, 111.6, 110.4, 70.6, 70.5, 70.4, 69.5, 69.5, 64.7, 64.2, 64.2, 54.1, 49.6, 42.4, 39.8, 35.9, 31.9, 29.6 (3 C), 27.3, 22.1, 1.9. HRMS (ESI, m/z) for $C_{50}H_{54}ClN_5O_{11}$ [M + H]: expected, 936.3581; found, 936.3582; 0.1 ppm.

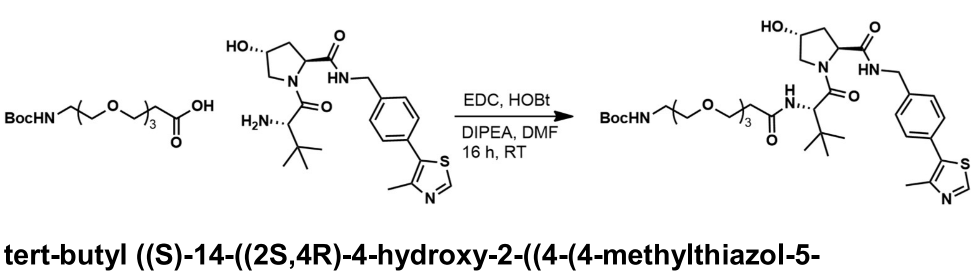

tert-butyl ((S)-14-((2S,4R)-4-hydroxy-2-((4-(4-methylthiazol-5-yl)benzyl)carbamoyl)pyrrolidine-1-carbonyl)-15,15-dimethyl-12-oxo-3,6,9-trioxa-13-azahexadecyl)carbamate. To a stirred solution of 2,2-dimethyl-4-oxo-3,8,11,14-tetraoxa-5-azaheptadecan-17-oic acid (200 mg, 0.622 mmol) in DMF (4 mL), DIPEA (193 mg, 1.49 mmol)

was added, then EDC (143 mg, 0.747 mmol) and HOBt (155 mg, 0.809 mmol) were added and the solution was stirred for 20 min. A solution of (2S,4R)-1-((S)-2-amino-3,3-dimethylbutanoyl)-4-hydroxy-N-(4-(4-methylthiazol-5-yl)benzyl)pyrrolidine-2-carboxamide hydrochloride (291 mg, 0.622 mmol) in DMF was then added, and the reaction was stirred for 16 h at room temperature. The reaction was quenched with deionized water, extracted with EtOAc, and washed with 0.1 M aq. HCl, saturated sodium bicarbonate, and brine. The organic phase was dried over anhydrous sodium sulfate, filtered, and concentrated under reduced pressure by rotary evaporation. The crude residue was purified by flash column chromatography (SiO_2). The product was obtained in 64% yield (292 mg) as a yellow solid. ^{1}H NMR (400 MHz, Chloroform-*d*) δ 8.70 (s, 1H), 7.44 – 7.32 (m, 5H), 6.96 (d, *J* = 7.9 Hz, 1H), 5.08 (s, 1H), 4.75 (t, *J* = 8.0 Hz, 1H), 4.58 (dd, *J* = 15.0, 6.7 Hz, 1H), 4.51 (s, 1H), 4.43 (d, *J* = 8.1 Hz, 1H), 4.33 (dd, *J* = 15.0, 5.2 Hz, 1H), 4.19 – 4.13 (m, 1H), 3.73 (t, *J* = 5.6 Hz, 2H), 3.64 (s, 4H), 3.60 (s, 5H), 3.57 – 3.47 (m, 3H), 3.29 (d, *J* = 6.3 Hz, 2H), 3.08 (s, 1H), 2.55 (s, 1H), 2.52 (d, *J* = 1.5 Hz, 3H), 2.49 (dt, *J* = 8.3, 3.9 Hz, 2H), 2.13 (dd, *J* = 13.5, 8.3 Hz, 1H), 1.43 (s, 9H), 0.93 (s, 9H). To the isolated intermediate, 4 mL of a 3:1 v/v mixture of DCM:TFA was added and stirred for 3 h at 35 °C. The solvent was then evaporated under a stream of N_2 and the crude TFA salt used without further purification.

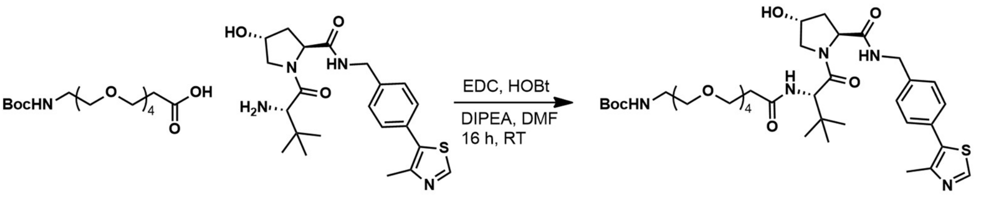

tert-butyl ((S)-17-((2S,4R)-4-hydroxy-2-((4-(4-methylthiazol-5-yl)benzyl)carbamoyl)pyrrolidine-1-carbonyl)-18,18-dimethyl-15-oxo-3,6,9,12-tetraoxa-16-azanonadecyl)carbamate. To a stirred solution of 5,8,11,14-tetraoxa-2-azaheptadecanedioic acid 1-(1,1-dimethylethyl) ester (200 mg, 0.547 mmol) in DMF (10 mL), DIPEA (170 mg, 1.31 mmol) was added, then EDC (126 mg, 0.657 mmol) and HOBt (136 mg, 0.712 mmol) were added and the solution was stirred for 20 min. A solution of (2S,4R)-1-((S)-2-amino-3,3-dimethylbutanoyl)-4-hydroxy-N-(4-(4-methylthiazol-5-yl)benzyl)pyrrolidine-2-carboxamide hydrochloride (256 mg, 0.547 mmol) in DMF was then added, and the reaction was stirred for 16 h at room temperature. The reaction was quenched with deionized water, extracted with EtOAc, and washed with 0.1 M aq. HCl, saturated sodium bicarbonate, and brine. The organic phase was dried over anhydrous sodium sulfate, filtered, and concentrated under reduced pressure by rotary evaporation. The crude residue was purified by flash column chromatography (SiO_2). The product was obtained in 62% yield (262 mg) as a yellow solid. ^{1}H NMR (400 MHz, Chloroform-*d*) δ 8.69 (s, 1H), 7.42 (s, 1H), 7.39 – 7.32 (m, 5H), 7.00 (d, *J* = 8.0 Hz, 1H), 5.09 (s, 1H), 4.74 (t, *J* = 8.0 Hz, 1H), 4.57 (dd, *J* = 15.0, 6.7 Hz, 1H), 4.50 (s, 1H), 4.43 (d, *J* = 8.1 Hz, 1H), 4.33 (dd, *J* = 15.0, 5.2 Hz, 1H), 4.14 (s, 1H), 3.71 (t, *J* = 5.7 Hz, 3H), 3.66 – 3.57 (m, 16H), 3.57 – 3.49 (m, 3H), 3.30 (d, *J* = 5.6 Hz, 4H), 2.56 (s, 1H), 2.52 (s, 4H), 2.48 (dt, *J* = 5.6, 3.0 Hz, 2H), 2.12 (dd, *J* = 13.6, 8.3 Hz, 1H), 1.43 (s, 11H), 0.93 (s, 11H). To the isolated intermediate, 4 mL of a 3:1 v/v mixture of DCM:TFA was added and stirred for 3 h at 35 °C. The solvent was then evaporated under a stream of N_2 and the crude TFA salt used without further purification.

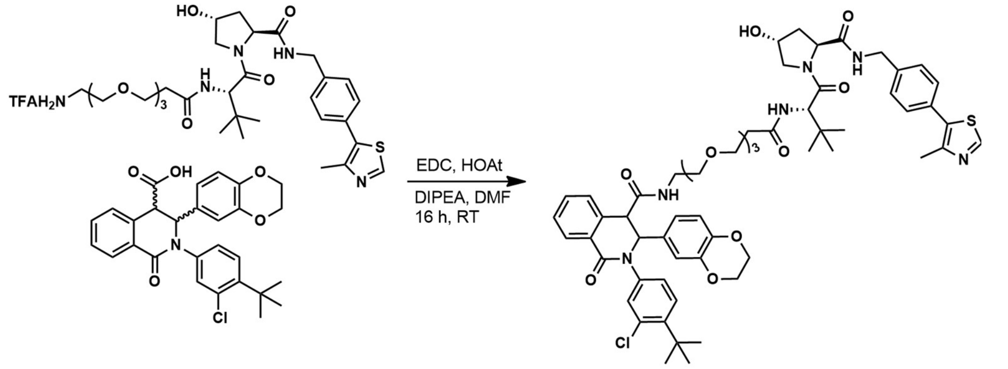

2-(4-(tert-butyl)-3-chlorophenyl)-3-(2,3-dihydrobenzo[b][1,4]dioxin-6-yl)-N-((S)-14-((2S,4R)-4-hydroxy-2-((4-(4-methylthiazol-5-yl)benzyl)carbamoyl)pyrrolidine-1-carbonyl)-15,15-dimethyl-12-oxo-3,6,9-trioxa-13-azahexadecyl)-1-oxo-1,2,3,4-tetrahydroisoquinoline-4-carboxamide (BH760) was synthesized in accordance with General Procedure 2, employing 2-chloro-4-(3-(2,3-dihydrobenzo[b][1,4]dioxin-6-yl)-4-(methoxycarbonyl)-1-oxo-3,4-dihydroisoquinolin-2(1H)-yl)benzoic acid (33 mg, 0.067 mmol), (2S,4R)-1-((S)-17-(tert-butyl)-1,1,1-trifluoro-2,15-dioxo-6,9,12-trioxa-3,16-diazaoctadecan-18-oyl)-4-hydroxy-N-(4-(4-methylthiazol-5-yl)benzyl)pyrrolidine-2-carboxamide (49 mg, 0.067 mmol), EDC (15 mg, 0.080 mmol), HOAt (17 mg, 0.087 mmol), and DIPEA (26 mg, 0.200 mmol). The product was obtained in 48% yield (36 mg) as a yellow amorphous solid. as a pair of diastereomers. The mixture of diastereomers was further purified by chiral HPLC (Phenomenex Lux Cellulose-1 column, 100% MeCN) to afford the separate isomers. ^{1}H NMR (400 MHz, Acetonitrile-d_3) δ 8.72 (s, 1H), 8.08 (dt, J = 7.4, 1.8 Hz, 1H), 7.55 – 7.44 (m, 3H), 7.39 (dt, J = 2.8, 1.3 Hz, 4H), 7.29 – 7.19 (m, 3H), 6.92 (d, J = 9.0 Hz, 1H), 6.71 – 6.60 (m, 3H), 6.48 (s, 1H), 5.57 (dd, J = 6.3, 1.9 Hz, 1H), 4.56 (dd, J = 9.1, 2.5 Hz, 1H), 4.44 (td, J = 13.7, 12.2, 6.3 Hz, 3H), 4.28 (ddd, J = 15.5, 5.7, 2.2 Hz, 1H), 4.13 (s, 4H), 4.00 (dd, J = 4.4, 1.9 Hz, 1H), 3.78 (d, J = 11.0 Hz, 1H), 3.70 – 3.61 (m, 3H), 3.50 (d, J = 1.8 Hz, 4H), 3.47 – 3.22 (m, 9H), 2.48 – 2.31 (m, 5H), 2.07 (dd, J = 8.5, 4.2 Hz, 2H), 1.44 (d, J = 1.3 Hz, 8H), 0.94 (s, 8H). ^{13}C NMR (151 MHz, CD_3CN) δ 173.3, 172.2, 172.2, 171.6, 164.6, 152.1, 149.8, 146.1, 145.2, 144.7, 143.0, 140.8, 135.6, 134.4, 134.3, 134.0, 132.9, 131.9, 131.4, 131.0, 130.6 (2C), 130.3, 130.0, 129.7, 129.7, 129.2 (2C), 126.3, 120.6, 116.5, 71.6, 71.5, 71.4, 71.2 (2C), 70.4, 68.4, 66.0, 65.8, 65.7, 60.6, 58.5, 58.0, 54.0, 43.6, 40.9, 38.8, 37.7, 37.1, 36.7, 30.3 (3C), 27.3 (3C), 16.9. HRMS (ESI, m/z) for $C_{59}H_{71}ClN_6O_{11}S$ [M + H]: expected, 1107.4662; found, 1107.4678; 1.5 ppm.

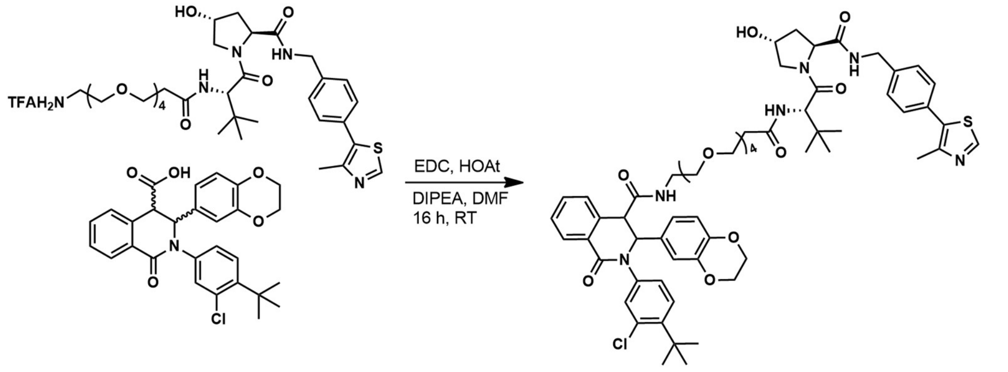

2-(4-(tert-butyl)-3-chlorophenyl)-3-(2,3-dihydrobenzo[b][1,4]dioxin-6-yl)-N-((S)-17-((2S,4R)-4-hydroxy-2-((4-(4-methylthiazol-5-yl)benzyl)carbamoyl)pyrrolidine-1-carbonyl)-18,18-dimethyl-15-oxo-3,6,9,12-tetraoxa-16-azanonadecyl)-1-oxo-1,2,3,4-tetrahydroisoquinoline-

4-carboxamide (BH761) was synthesized in accordance with General Procedure 2, employing 2-chloro-4-(3-(2,3-dihydrobenzo[b][1,4]dioxin-6-yl)-4-(methoxycarbonyl)-1-oxo-3,4-dihydroisoquinolin-2(1H)-yl)benzoic acid (32 mg, 0.065 mmol), (2S,4R)-1-((S)-20-(tert-butyl)-1,1,1-trifluoro-2,18-dioxo-6,9,12,15-tetraoxa-3,19-diazahenicosan-21-oyl)-4-hydroxy-N-(4-(4-methylthiazol-5-yl)benzyl)pyrrolidine-2-carboxamide (50 mg, 0.065 mmol), EDC (15 mg, 0.078 mmol), HOAt (16 mg, 0.085 mmol), and DIPEA (20 mg, 0.160 mmol). The product was obtained in 72% yield (54 mg) as a yellow amorphous solid as a pair of diastereomers. The mixture of diastereomers was further purified by chiral HPLC (Phenomenex Lux Cellulose-1 column, 100% MeCN) to afford the separate isomers. ^{1}H NMR (400 MHz, Acetonitrile-d_3) δ 8.73 (s, 1H), 8.11 – 8.06 (m, 1H), 7.55 – 7.44 (m, 3H), 7.39 (d, *J* = 1.6 Hz, 4H), 7.28 – 7.18 (m, 3H), 6.92 (d, *J* = 9.0 Hz, 1H), 6.71 – 6.60 (m, 3H), 6.46 (s, 1H), 5.57 (t, *J* = 2.3 Hz, 1H), 4.58 – 4.53 (m, 1H), 4.51 – 4.37 (m, 3H), 4.33 – 4.25 (m, 1H), 4.14 (s, 4H), 3.99 (t, *J* = 2.2 Hz, 1H), 3.78 (d, *J* = 11.0 Hz, 1H), 3.65 (ddt, *J* = 11.9, 6.0, 2.7 Hz, 3H), 3.52 (s, 4H), 3.45 – 3.37 (m, 5H), 3.37 – 3.25 (m, 3H), 2.45 (d, *J* = 1.2 Hz, 3H), 2.43 – 2.32 (m, 2H), 2.08 (dd, *J* = 8.6, 3.9 Hz, 2H), 1.45 (s, 8H), 0.94 (s, 8H). ^{13}C NMR (151 MHz, CD_3CN) δ 173.3, 172.2, 172.2, 171.6, 164.6, 152.1, 149.8, 146.1, 145.2, 144.7, 143.0, 140.8, 135.6, 134.4, 134.3, 134.0, 132.9, 131.9, 131.4, 131.0, 130.6 (2C), 130.3, 130.0, 129.7, 129.6, 129.2, 126.3, 120.6, 116.5, 71.6, 71.6, 71.5, 71.5, 71.4, 71.2 (2C), 70.5, 68.4, 66.0, 65.8, 65.7, 60.6, 58.5, 58.0, 54.0, 43.6, 40.9, 38.8, 37.7, 37.1, 36.7, 30.3 (3C), 27.3 (3C), 16.9. HRMS (ESI, m/z) for $C_{61}H_{75}ClN_6O_{12}S$ [M + H]: expected, 1151.4925; found, 1151.4933; 0.8 ppm.

NMR spectra

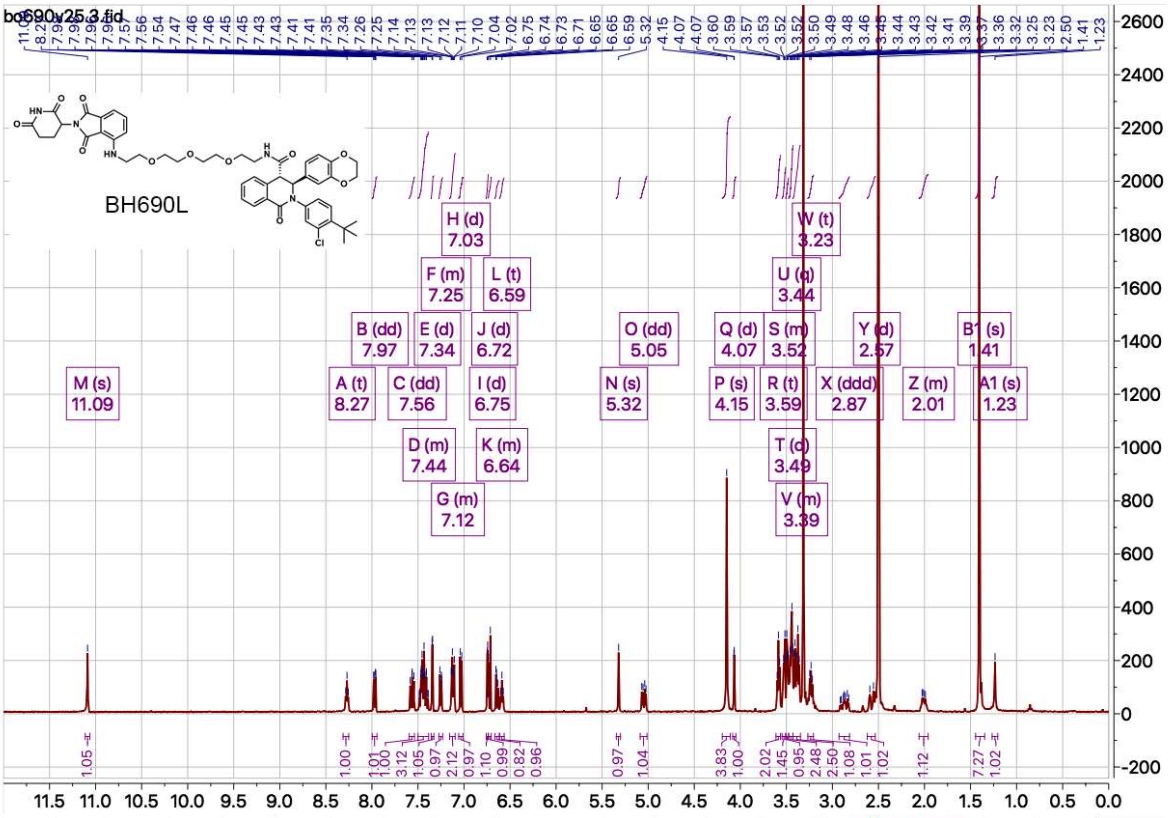

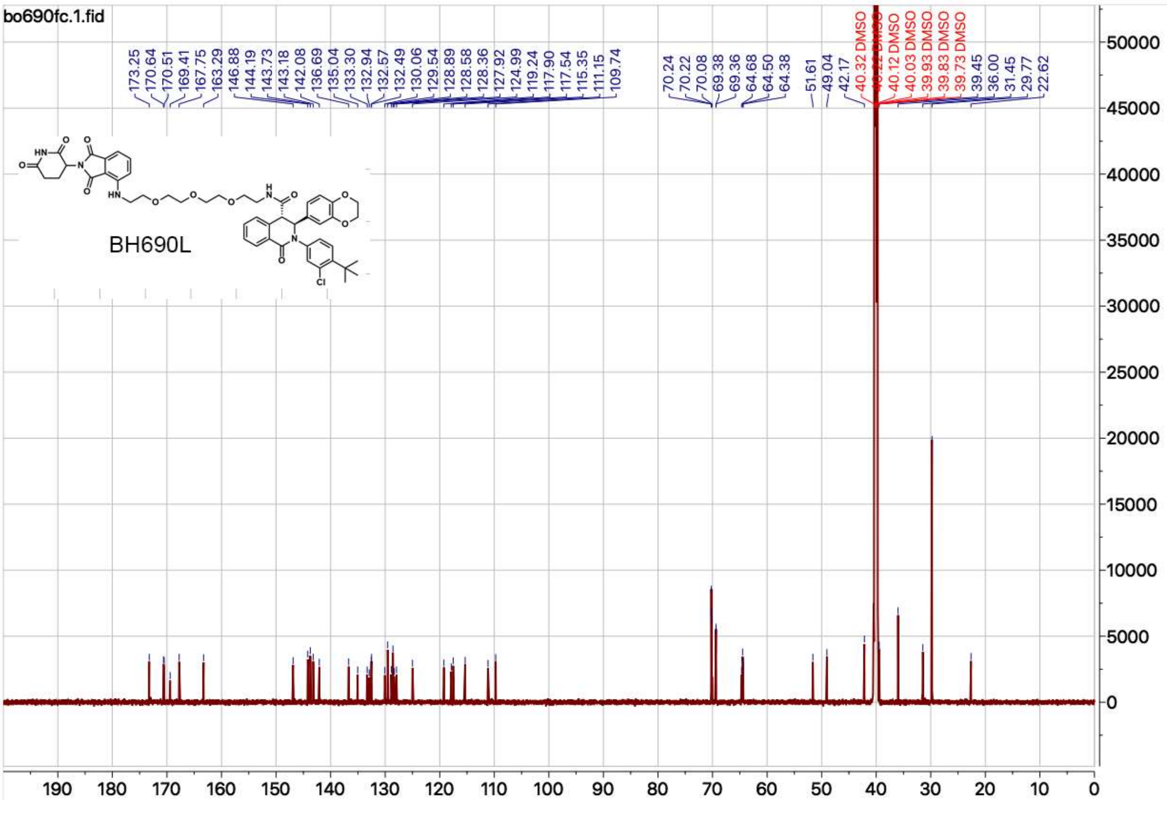

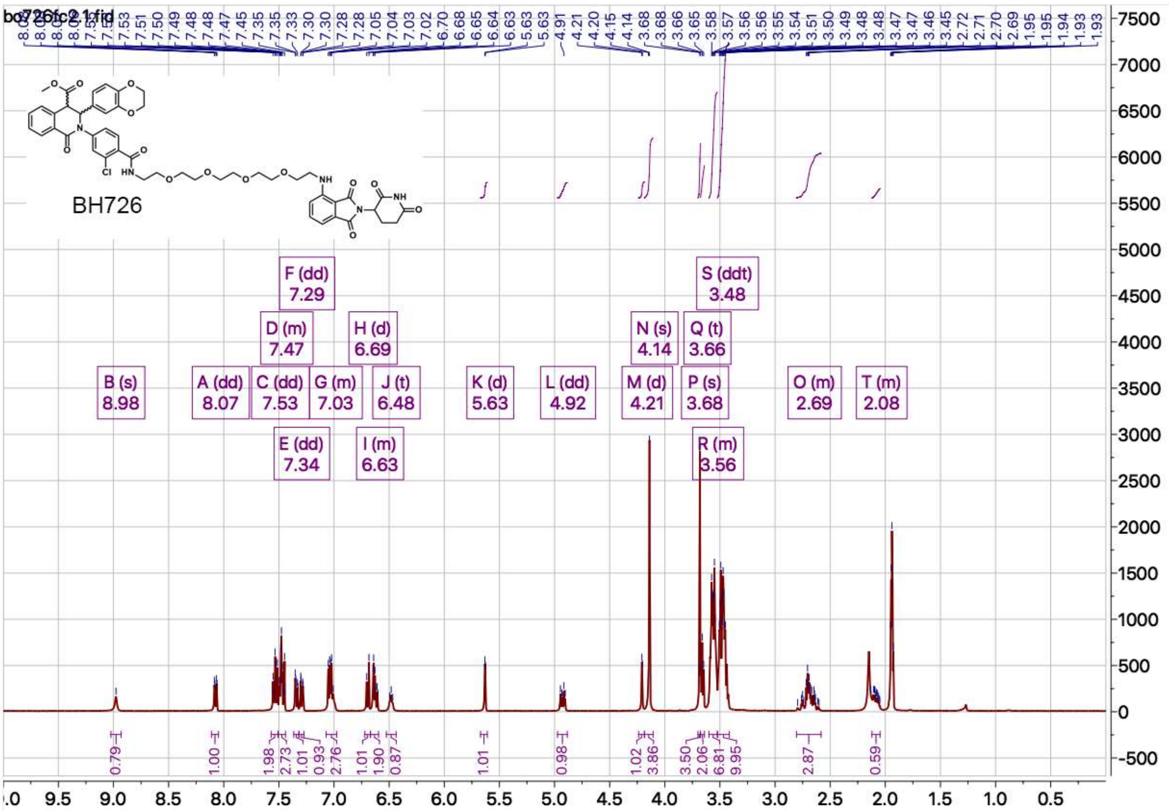
bo726fc2.1.fid
BH726
B (s) 8.98
A (dd) 8.07
C (dd) 7.53
D (m) 7.47
E (dd) 7.34
F (dd) 7.29
G (m) 7.03
H (d) 6.69
I (m) 6.63
J (t) 6.48
K (d) 5.63
L (dd) 4.92
M (d) 4.21
N (s) 4.14
P (s) 3.68
Q (t) 3.66
R (m) 3.56
S (ddt) 3.48
O (m) 2.69
T (m) 2.08

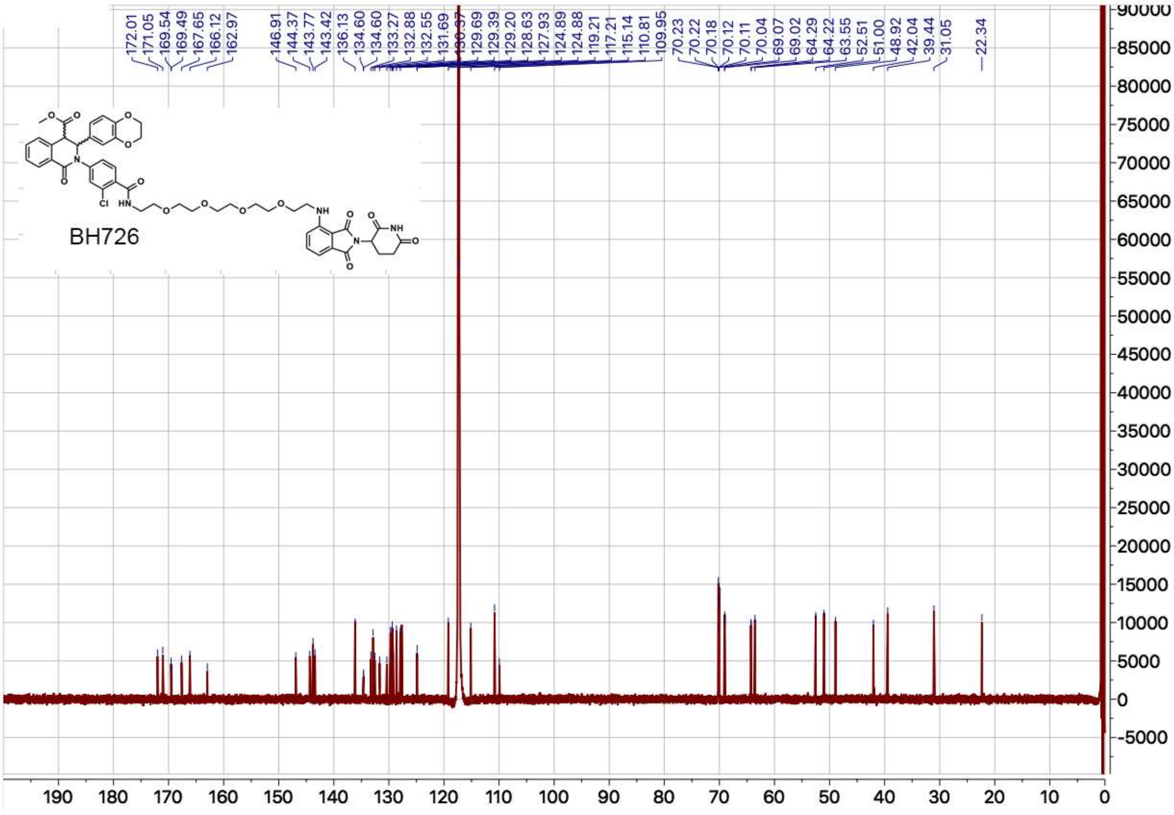
BH726
172.01
171.05
169.54
169.49
167.65
166.12
162.97
146.91
144.37
143.77
143.42
136.13
134.60
134.60
133.27
132.88
132.55
131.69
129.69
129.39
129.20
128.63
127.93
124.89
124.88
119.21
117.21
115.14
110.81
109.95
70.23
70.22
70.18
70.12
70.11
70.04
69.07
69.02
64.29
64.22
63.55
52.51
51.00
48.92
42.04
39.44
31.05
22.34

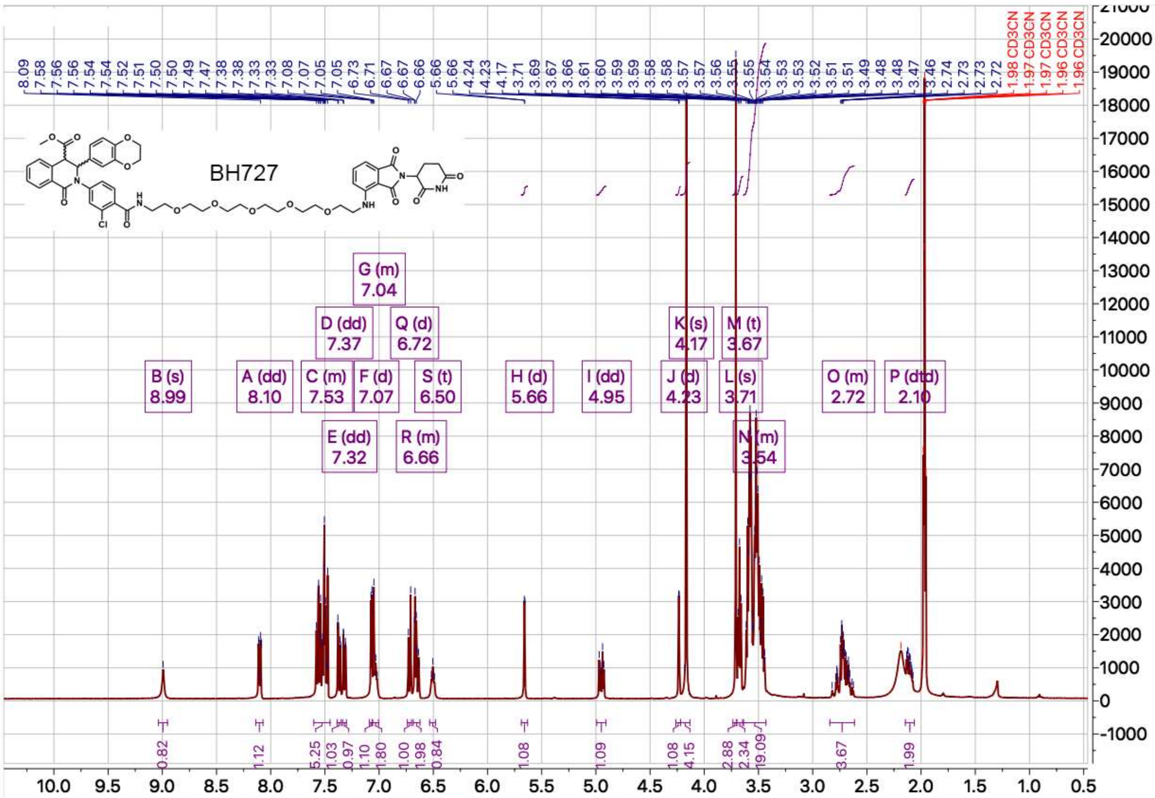
BH727
B (s) 8.99
A (dd) 8.10
C (m) 7.53
D (dd) 7.37
E (dd) 7.32
F (d) 7.07
G (m) 7.04
Q (d) 6.72
R (m) 6.66
S (t) 6.50
H (d) 5.66
I (dd) 4.95
J (d) 4.23
K (s) 4.17
L (s) 3.71
M (t) 3.67
N (m) 3.54
O (m) 2.72
P (dtd) 2.10
1.98 CD3CN
1.97 CD3CN
1.96 CD3CN
1.96 CD3CN

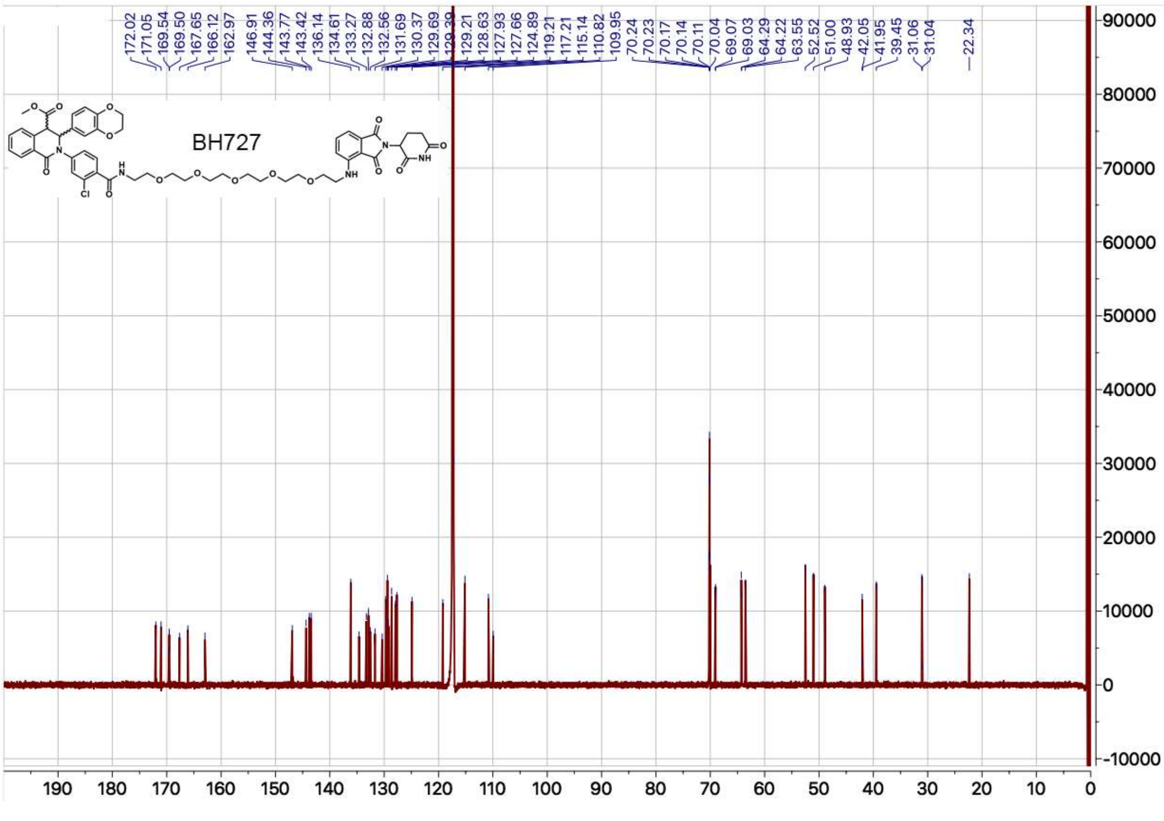
BH727

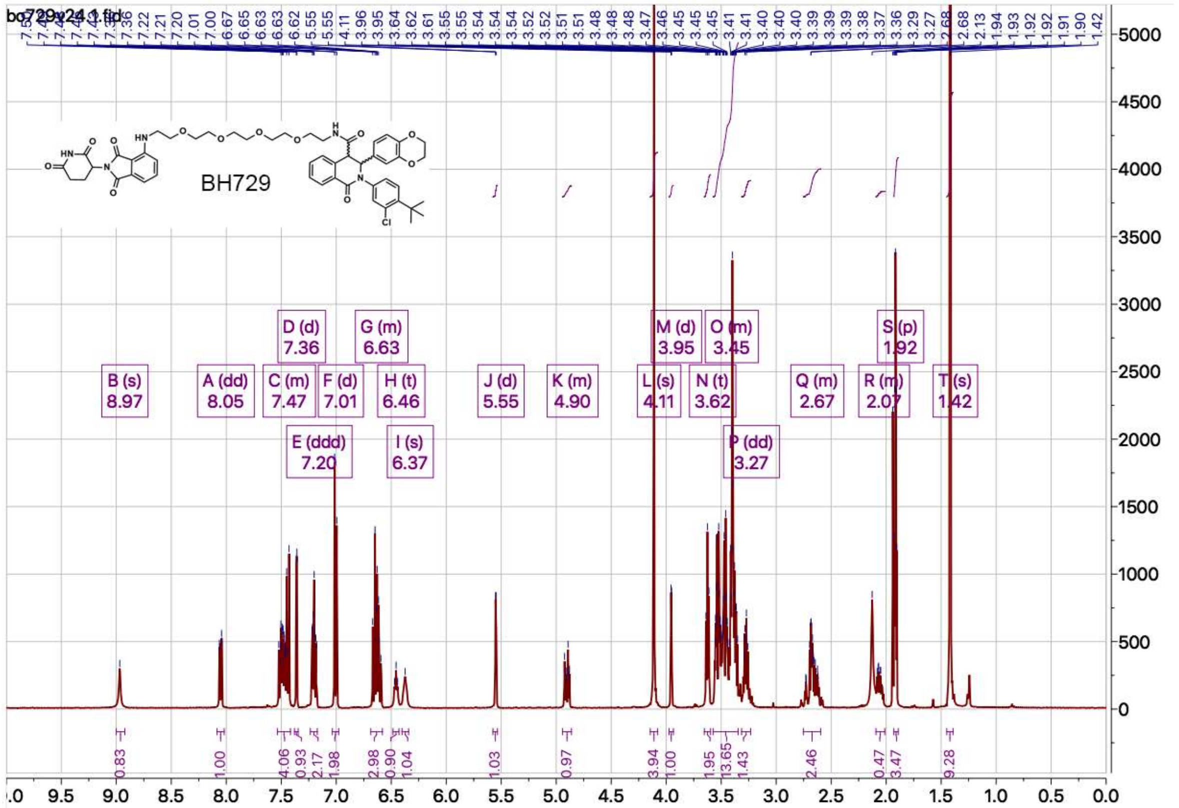

bo729y24.1.fid
BH729
B (s) 8.97
A (dd) 8.05
C (m) 7.47
D (d) 7.36
E (ddd) 7.20
F (d) 7.01
G (m) 6.63
H (t) 6.46
I (s) 6.37
J (d) 5.55
K (m) 4.90
L (s) 4.11
M (d) 3.95
N (t) 3.62
O (m) 3.45
P (dd) 3.27
Q (m) 2.67
R (m) 2.07
S (p) 1.92
T (s) 1.42
0.83
1.00
4.06
0.93
2.17
1.98
2.98
0.90
1.04
1.03
0.97
3.94
1.00
1.95
13.65
1.43
2.46
0.47
3.47
9.28

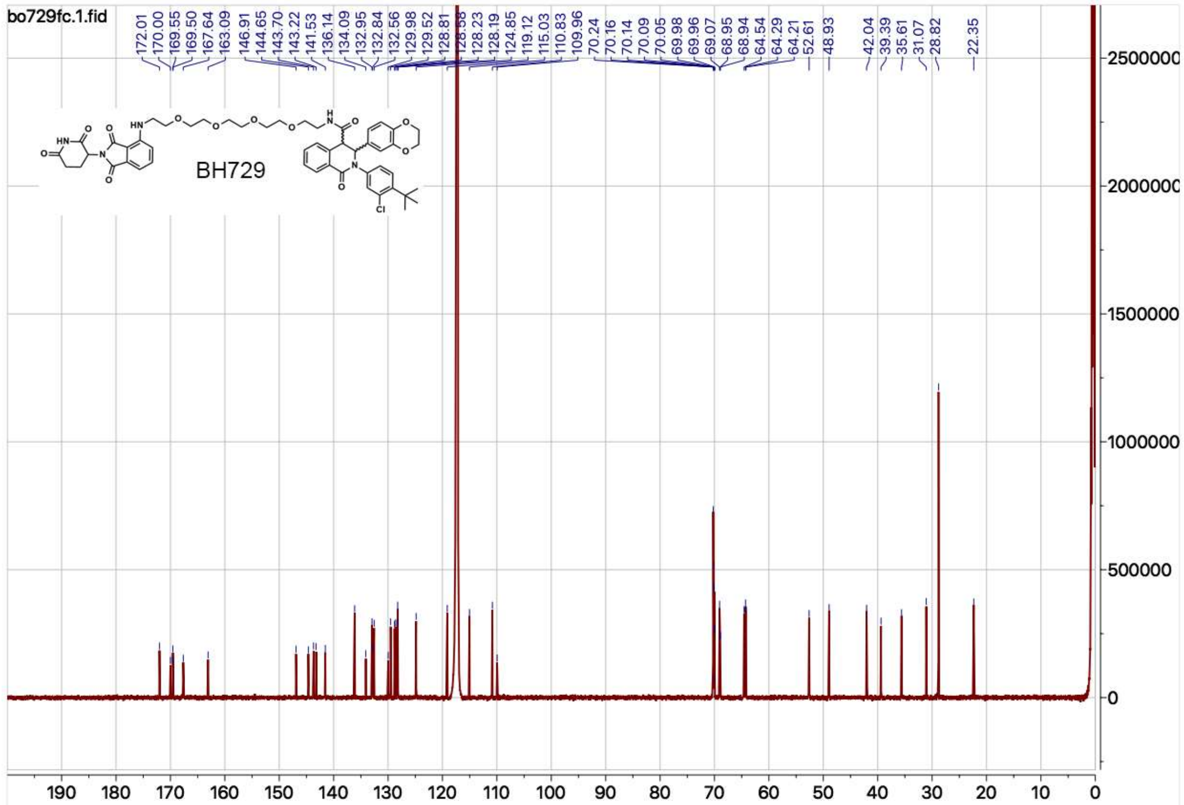

bo729fc.1.fid
172.01
170.00
169.55
169.50
167.64
163.09
146.91
144.65
143.70
143.22
141.53
136.14
134.09
132.95
132.84
132.56
129.98
129.52
128.81
128.23
128.19
124.85
119.12
115.03
110.83
109.96
70.24
70.16
70.14
70.09
70.05
69.96
69.07
68.95
68.94
64.54
64.29
64.21
52.61
48.93
42.04
39.39
35.61
31.07
28.82
22.35
BH729

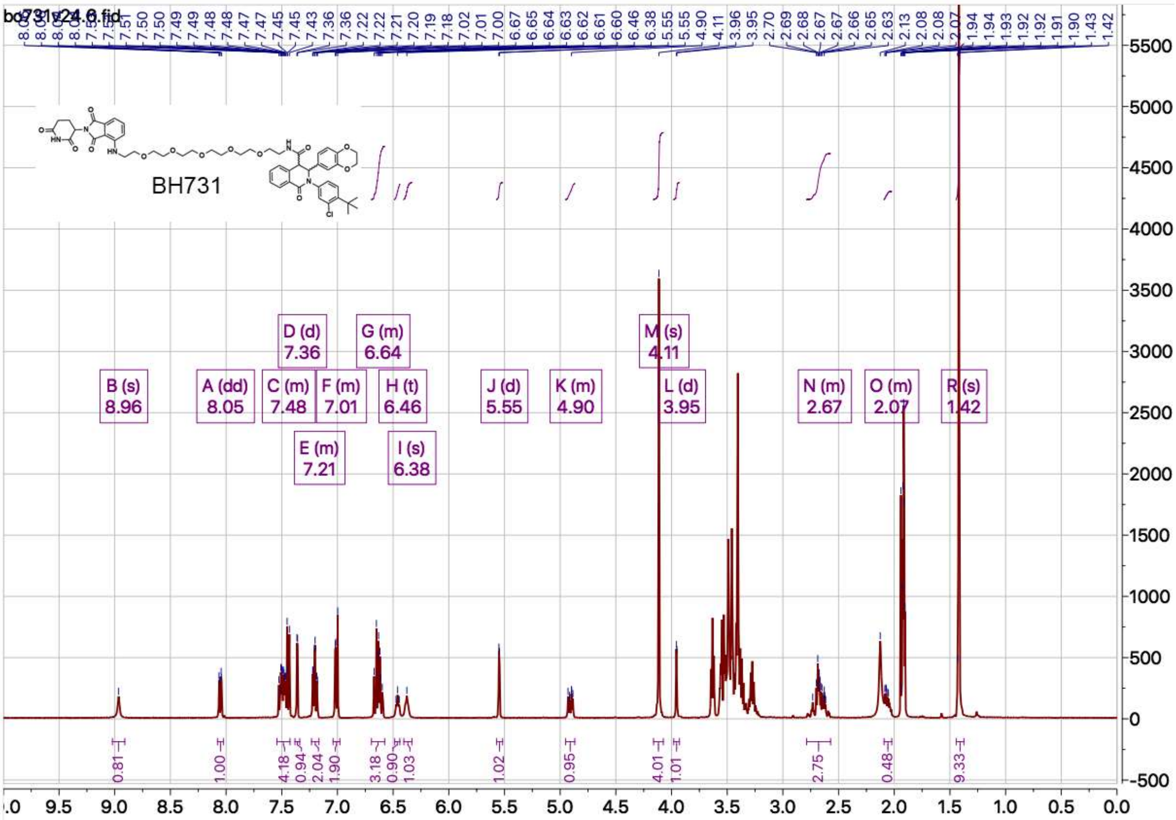
bo731v24.6.fid
BH731
B (s) 8.96
A (dd) 8.05
C (m) 7.48
D (d) 7.36
E (m) 7.21
F (m) 7.01
G (m) 6.64
H (t) 6.46
I (s) 6.38
J (d) 5.55
K (m) 4.90
M (s) 4.11
L (d) 3.95
N (m) 2.67
O (m) 2.07
R (s) 1.42
0.81
1.00
4.18
0.94
2.04
1.90
3.18
0.90
1.03
1.02
0.95
4.01
1.01
2.75
0.48
9.33

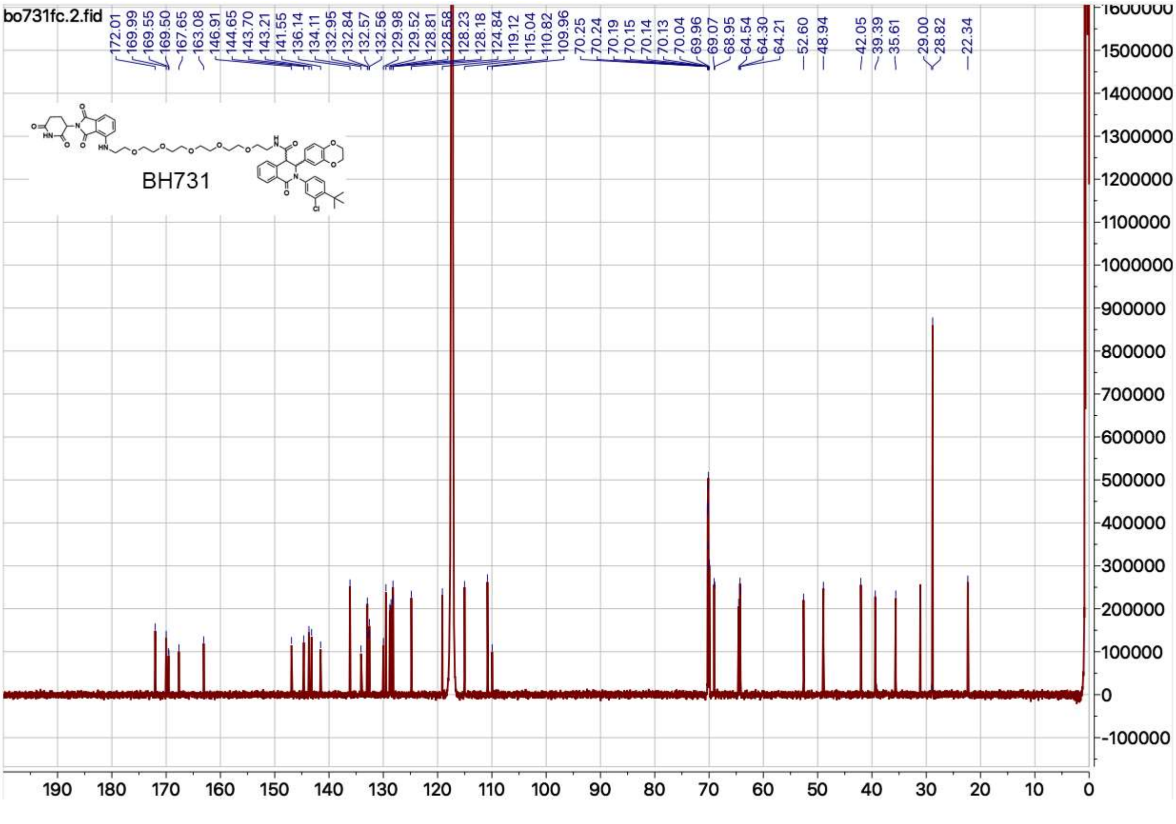
bo731fc.2.fid
172.01
169.99
169.55
169.50
167.65
163.08
146.91
144.65
143.70
143.21
141.55
136.14
134.11
132.95
132.84
132.57
132.56
129.98
129.52
128.81
128.58
128.23
128.18
124.84
119.12
115.04
110.82
109.96
70.25
70.24
70.19
70.15
70.14
70.13
70.04
69.96
69.07
68.95
64.54
64.30
64.21
52.60
48.94
42.05
39.39
35.61
29.00
28.82
22.34
BH731

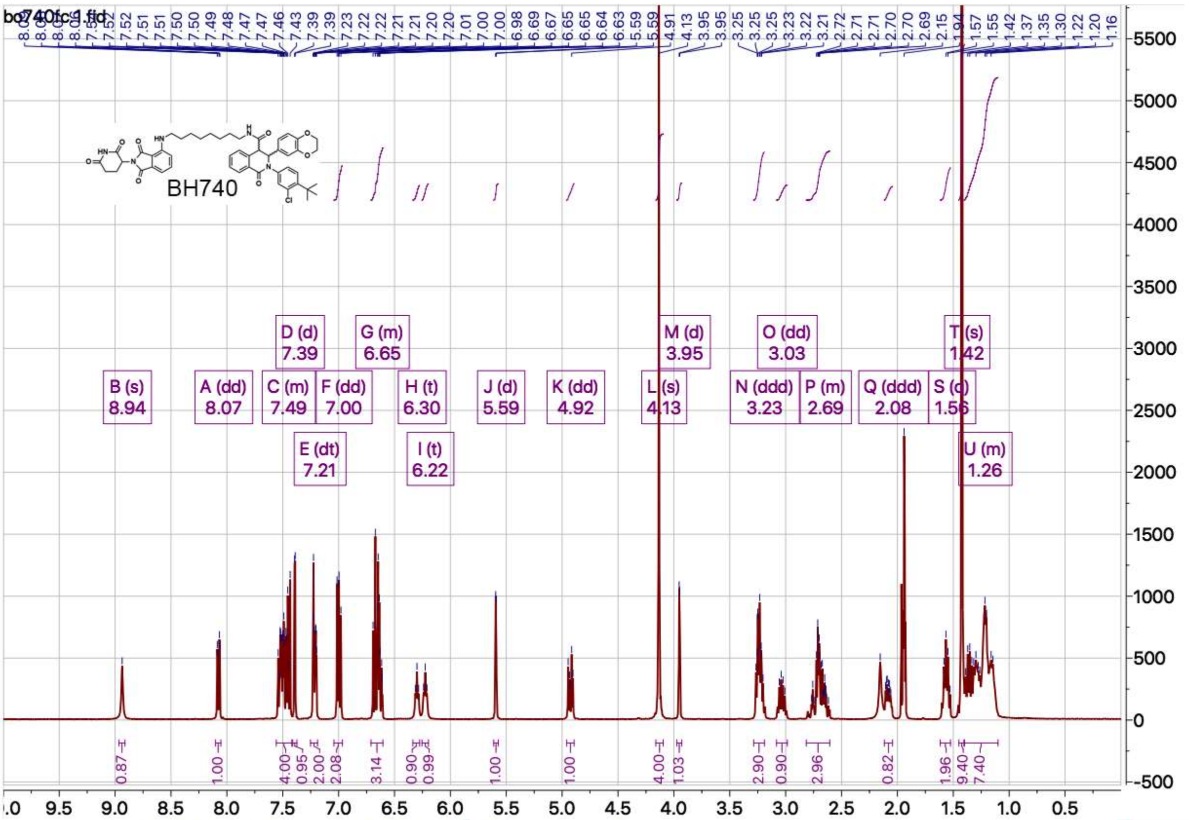
bo740fc.1.fid
BH740
B (s)
8.94
A (dd)
8.07
C (m)
7.49
D (d)
7.39
E (dt)
7.21
F (dd)
7.00
G (m)
6.65
H (t)
6.30
I (t)
6.22
J (d)
5.59
K (dd)
4.92
L (s)
4.13
M (d)
3.95
N (ddd)
3.23
O (dd)
3.03
P (m)
2.69
Q (ddd)
2.08
S (d)
1.56
T (s)
1.42
U (m)
1.26

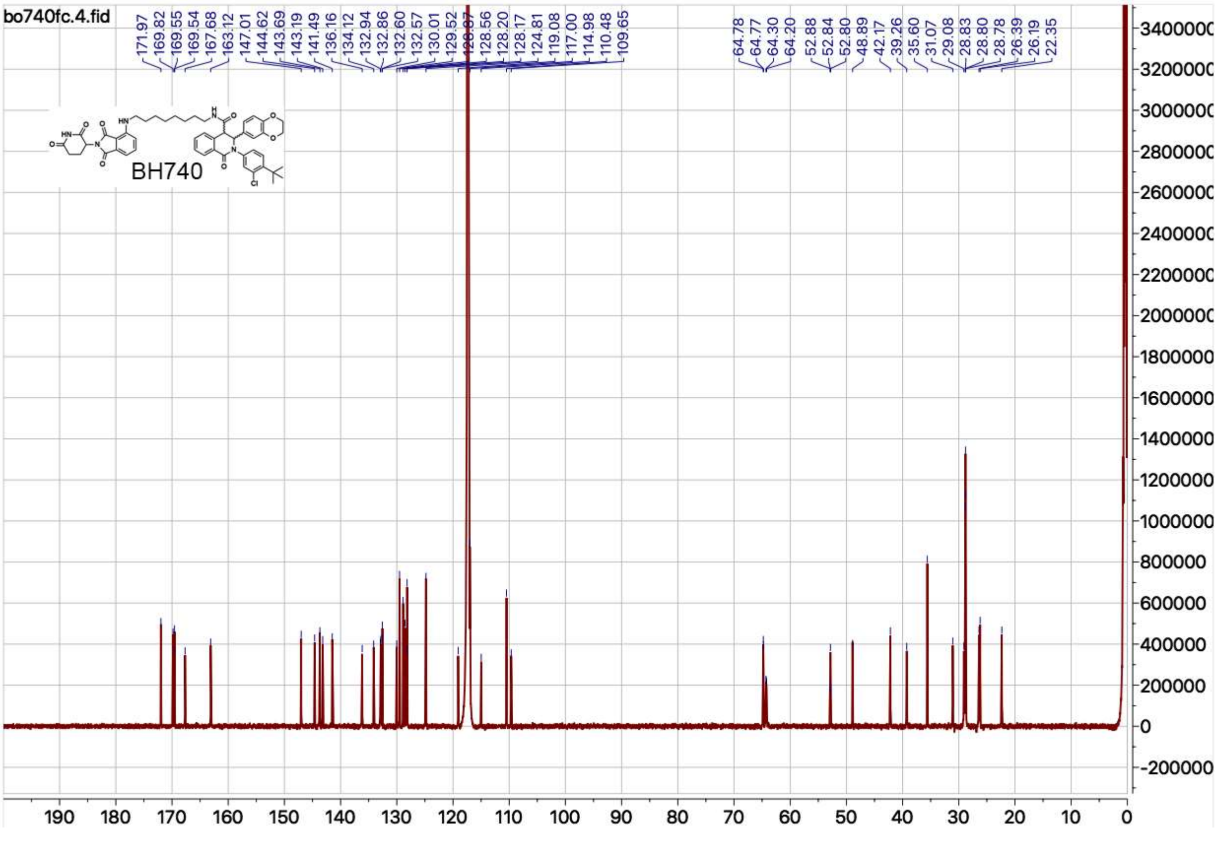
bo740fc.4.fid
BH740
171.97
169.82
169.55
169.54
167.68
163.12
147.01
144.62
143.69
143.19
141.49
136.16
134.12
132.94
132.86
132.60
132.57
130.01
129.52
128.56
128.20
128.17
124.81
119.08
117.00
114.98
110.48
109.65
64.78
64.77
64.30
64.20
52.88
52.84
52.80
48.89
42.17
39.26
35.60
31.07
29.08
28.83
28.80
28.78
26.39
26.19
22.35

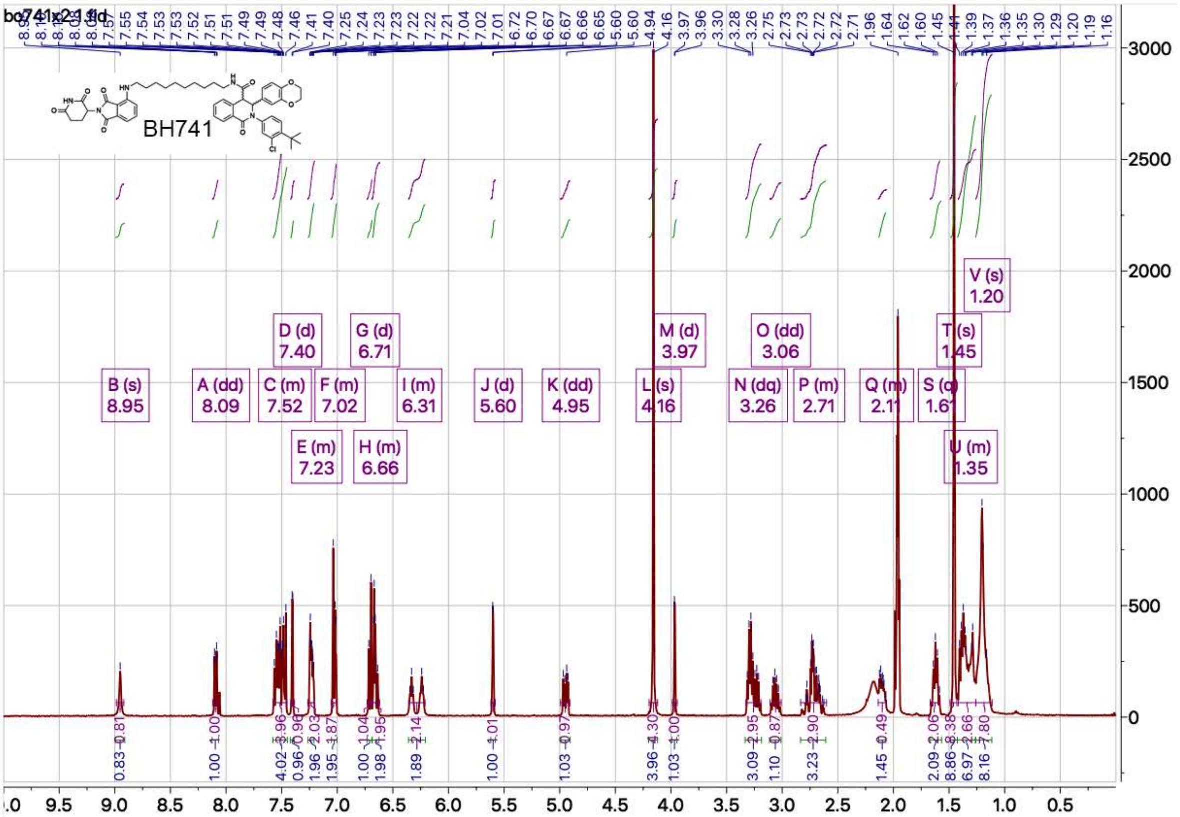
bo741x2.1.fid
BH741
B (s) 8.95
A (dd) 8.09
C (m) 7.52
D (d) 7.40
E (m) 7.23
F (m) 7.02
G (d) 6.71
H (m) 6.66
I (m) 6.31
J (d) 5.60
K (dd) 4.95
L (s) 4.16
M (d) 3.97
N (dq) 3.26
O (dd) 3.06
P (m) 2.71
Q (m) 2.11
S (q) 1.61
T (s) 1.45
U (m) 1.35
V (s) 1.20

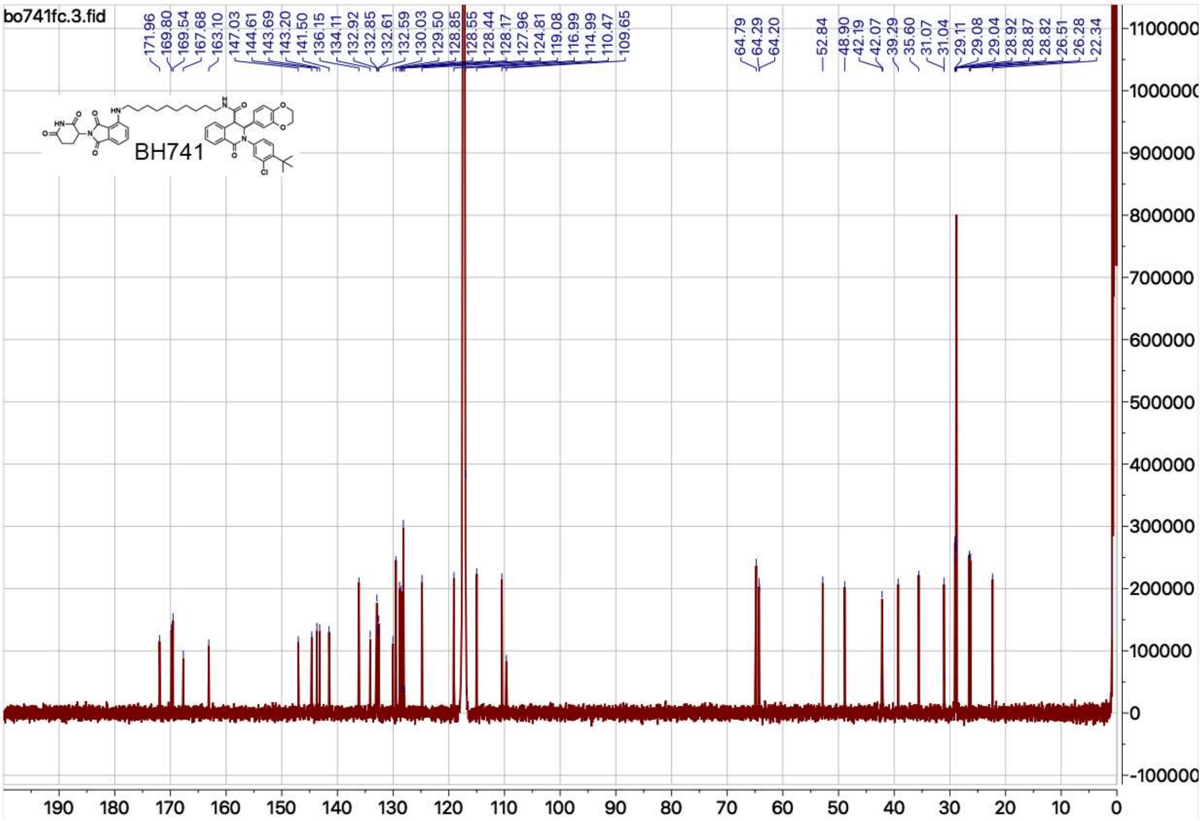
bo741fc.3.fid
BH741
171.96
169.80
169.54
167.68
163.10
147.03
144.61
143.69
143.20
141.50
136.15
134.11
132.92
132.85
132.61
132.59
130.03
129.50
128.85
128.55
128.44
128.17
127.96
124.81
119.08
116.99
114.99
110.47
109.65
64.79
64.29
64.20
52.84
48.90
42.19
42.07
39.29
35.60
31.07
31.04
29.11
29.08
29.04
28.92
28.87
28.82
26.51
26.28
22.34

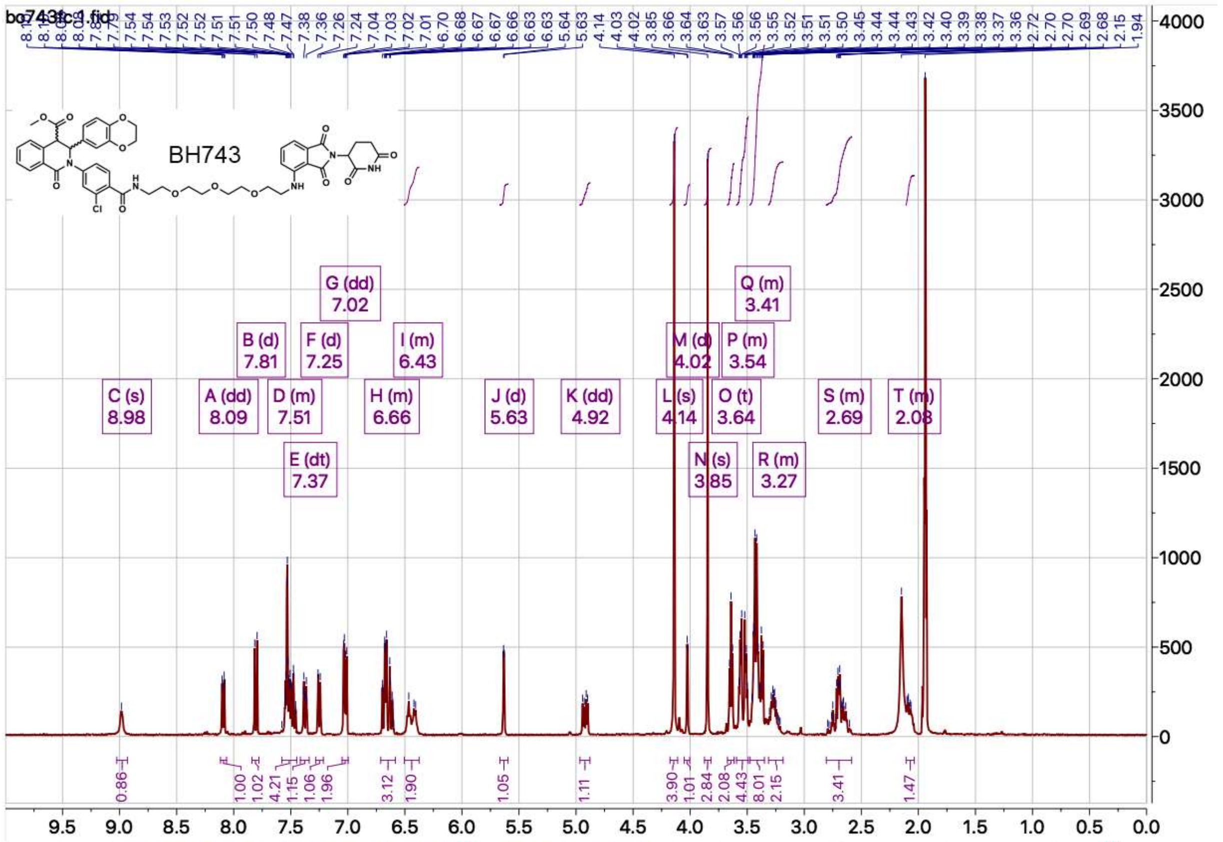
bo743fc.1.fid
BH743
C (s) 8.98
A (dd) 8.09
B (d) 7.81
D (m) 7.51
E (dt) 7.37
F (d) 7.25
G (dd) 7.02
H (m) 6.66
I (m) 6.43
J (d) 5.63
K (dd) 4.92
L (s) 4.14
M (d) 4.02
N (s) 3.85
O (t) 3.64
P (m) 3.54
Q (m) 3.41
R (m) 3.27
S (m) 2.69
T (m) 2.08
Cl
NH

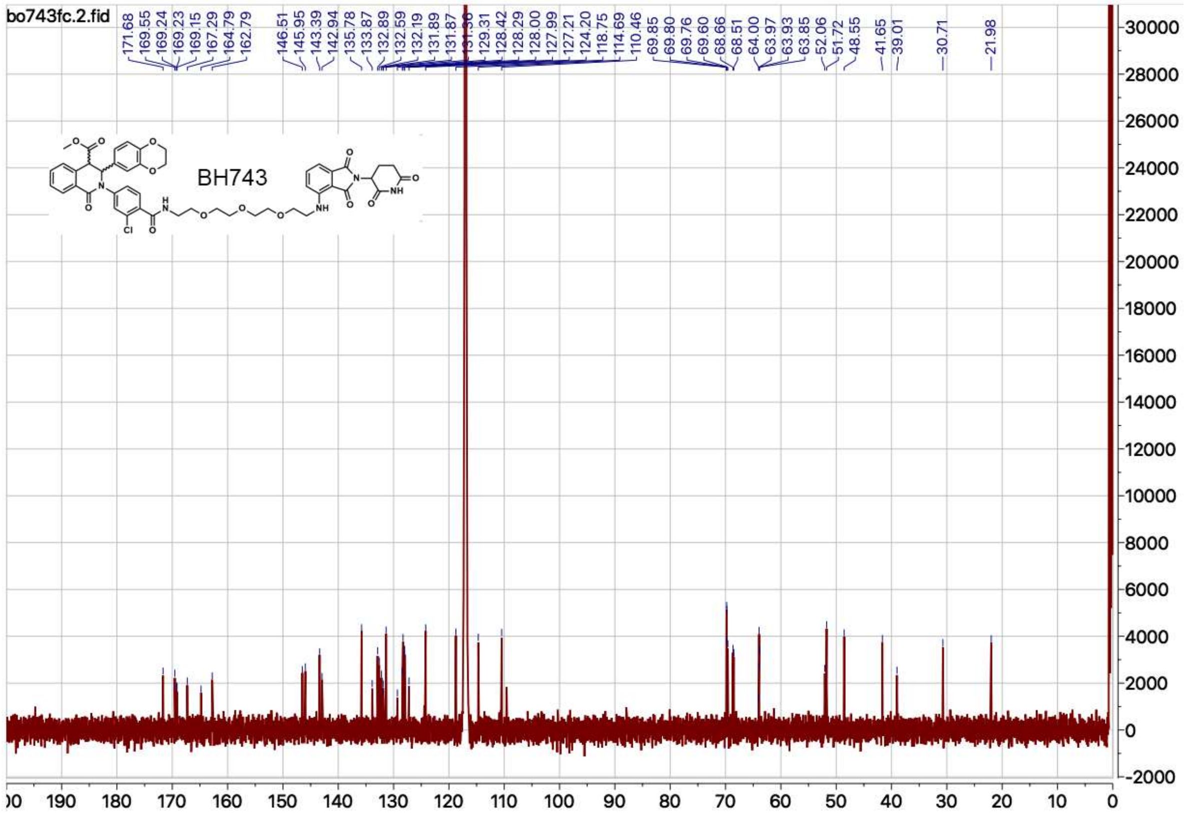
bo743fc.2.fid
BH743
Cl
NH

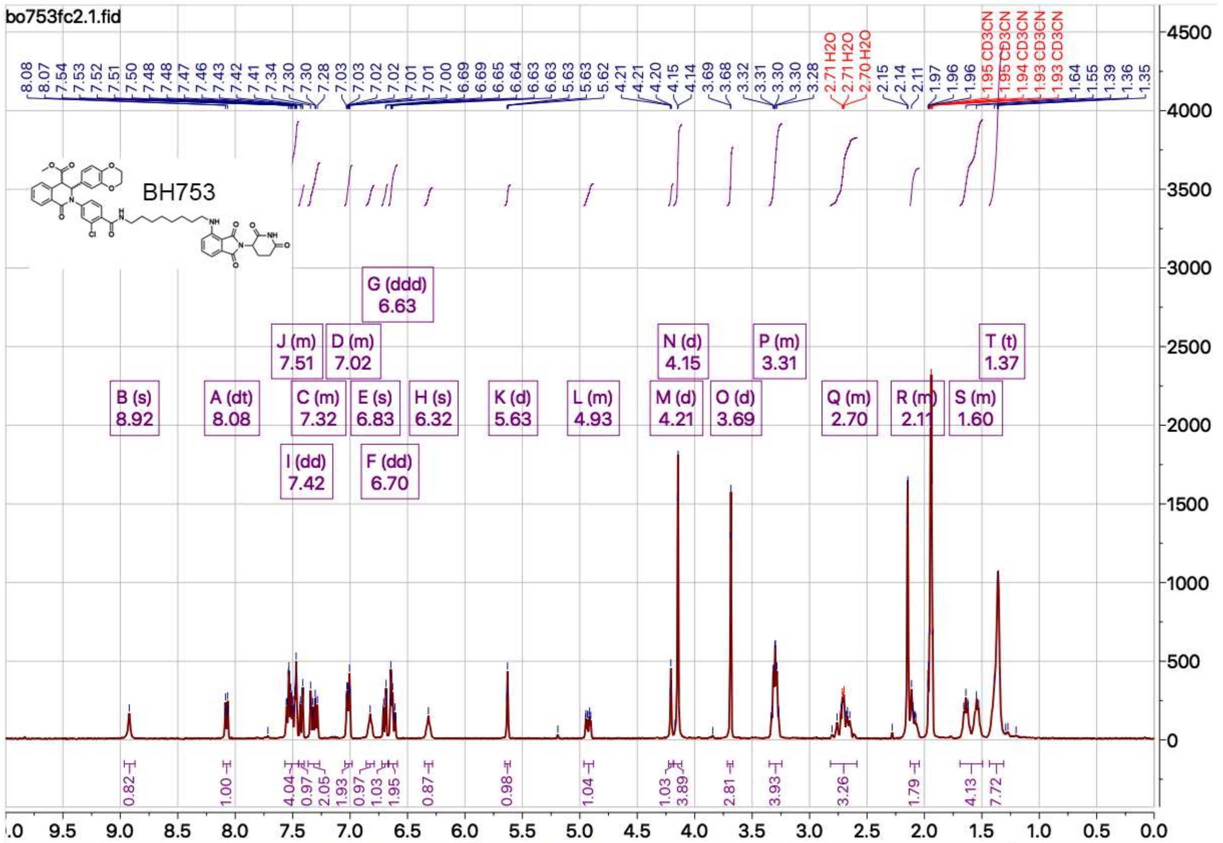
bo753fc2.1.fid
BH753
B (s) 8.92
A (dt) 8.08
J (m) 7.51
C (m) 7.32
I (dd) 7.42
D (m) 7.02
E (s) 6.83
G (ddd) 6.63
F (dd) 6.70
H (s) 6.32
K (d) 5.63
L (m) 4.93
N (d) 4.15
M (d) 4.21
O (d) 3.69
P (m) 3.31
Q (m) 2.70
R (m) 2.11
S (m) 1.60
T (t) 1.37

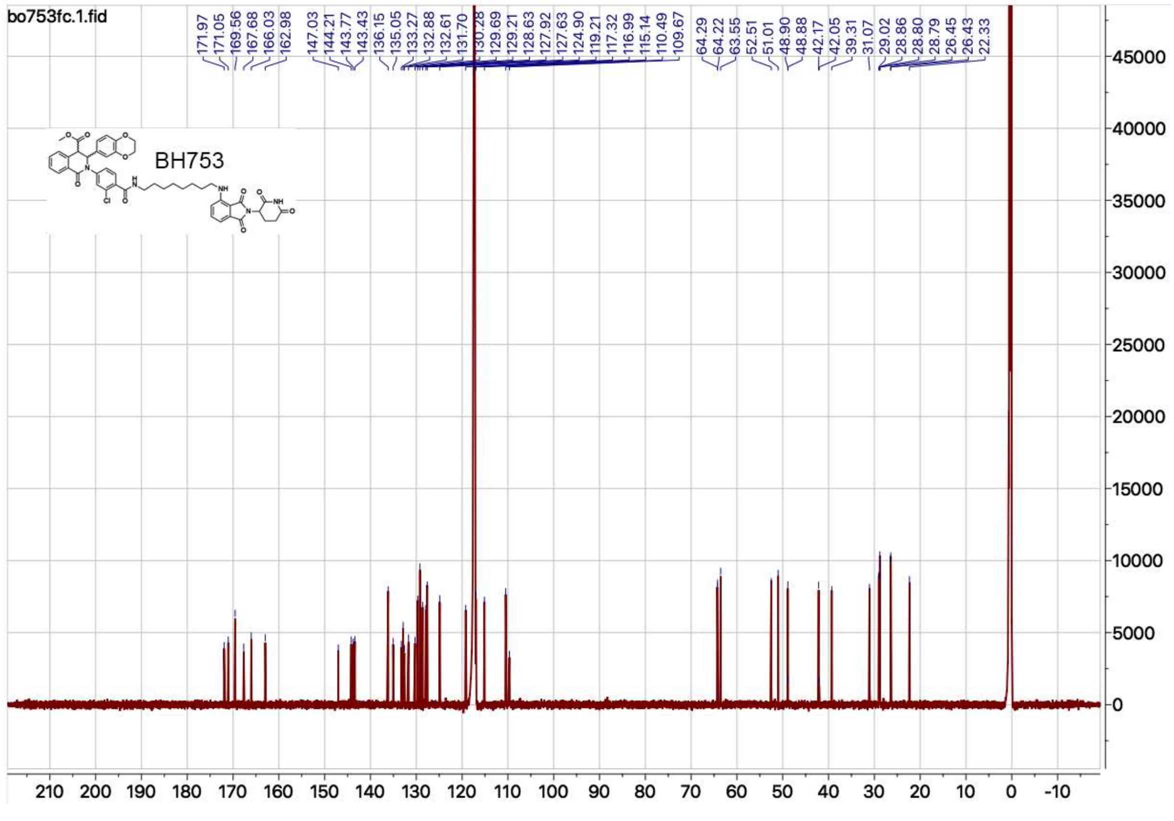
bo753fc.1.fid
BH753
171.97
171.05
169.56
167.68
166.03
162.98
147.03
144.21
143.77
143.43
136.15
135.05
133.27
132.88
132.61
131.70
130.28
129.69
129.21
128.63
127.92
127.63
124.90
119.21
117.32
116.99
115.14
110.49
109.67
64.29
64.22
63.55
52.51
51.01
48.90
48.88
42.17
42.05
39.31
31.07
29.02
28.86
28.80
28.79
26.45
26.43
22.33

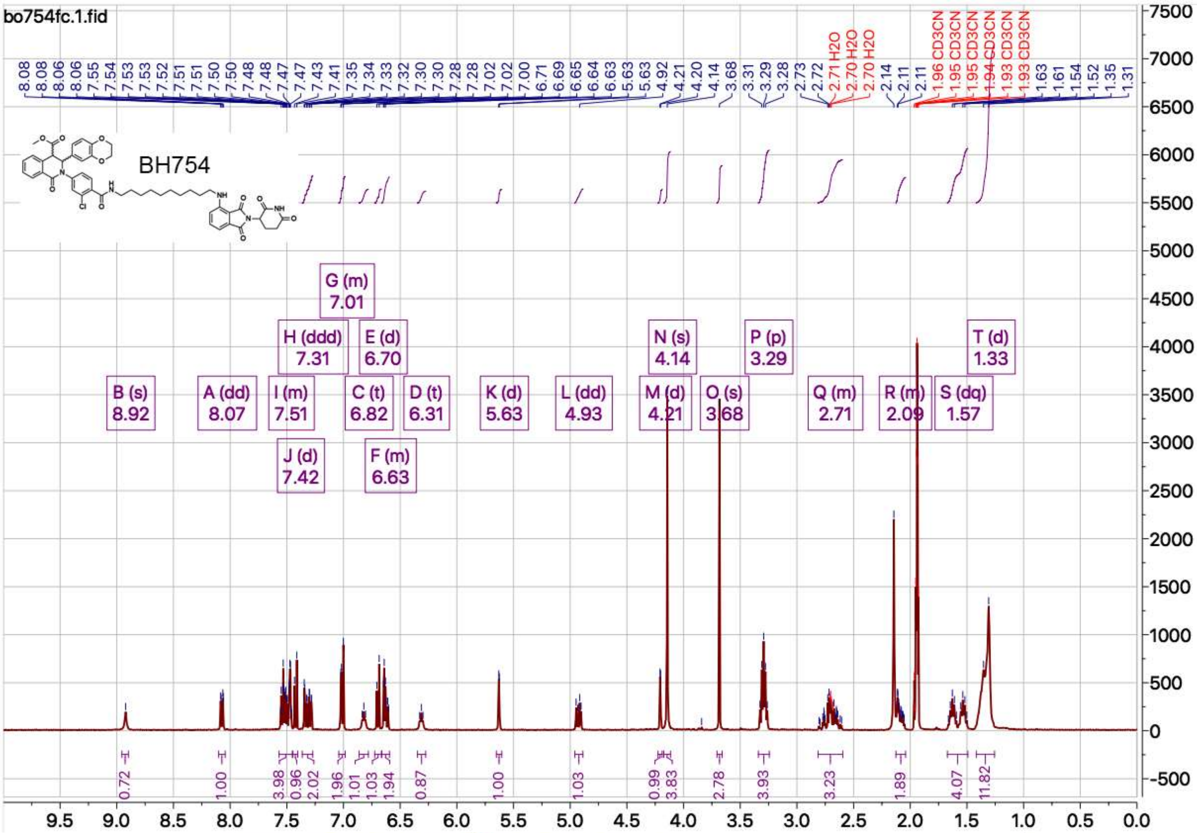
bo754fc.1.fid
BH754
8.08 8.08 8.06 8.06 7.55 7.54 7.53 7.53 7.52 7.51 7.51 7.50 7.50 7.48 7.48 7.47 7.47 7.43 7.41 7.35 7.34 7.33 7.32 7.30 7.30 7.28 7.28 7.02 7.02 7.00 6.71 6.69 6.65 6.64 6.63 5.63 5.63 4.92 4.21 4.20 4.14 3.68 3.31 3.29 3.28 2.73 2.72
2.71 H2O 2.70 H2O 2.70 H2O
2.14 2.11
1.96 CD3CN 1.95 CD3CN 1.95 CD3CN 1.94 CD3CN 1.93 CD3CN 1.93 CD3CN
1.63 1.61 1.54 1.52 1.35 1.31
B (s) 8.92
A (dd) 8.07
I (m) 7.51
J (d) 7.42
H (ddd) 7.31
G (m) 7.01
C (t) 6.82
E (d) 6.70
F (m) 6.63
D (t) 6.31
K (d) 5.63
L (dd) 4.93
M (d) 4.21
N (s) 4.14
O (s) 3.68
P (p) 3.29
Q (m) 2.71
R (m) 2.09
S (dq) 1.57
T (d) 1.33
0.72 1.00 3.98 0.96 2.02 1.96 1.01 1.03 1.94 0.87 1.00 1.03 0.99 3.83 2.78 3.93 3.23 1.89 4.07 11.82

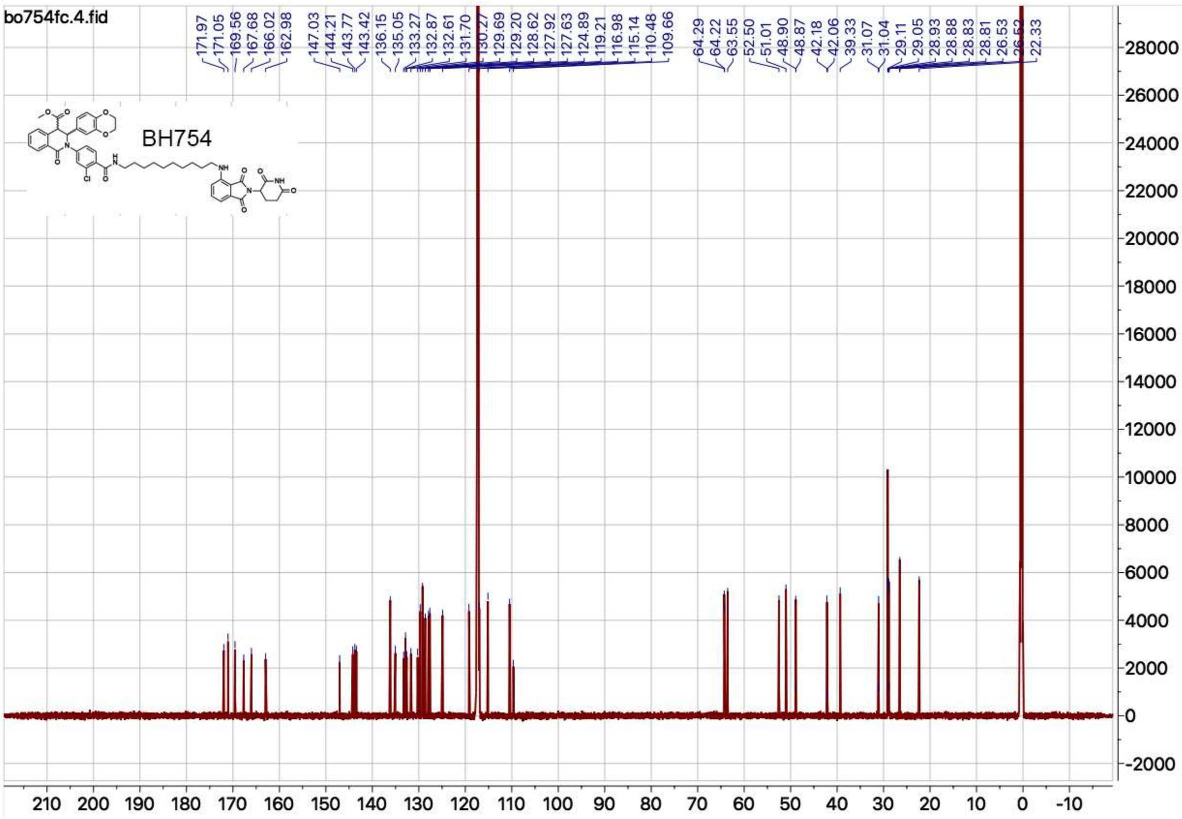
bo754fc.4.fid
BH754
171.97 171.05 169.56 167.68 166.02 162.98 147.03 144.21 143.77 143.42 136.15 135.05 133.27 132.87 132.61 131.70 130.27 129.69 129.20 128.62 127.92 127.63 124.89 119.21 116.98 115.14 110.48 109.66 64.29 64.22 63.55 52.50 51.01 48.90 48.87 42.18 42.06 39.33 31.07 31.04 29.11 29.05 28.93 28.88 28.83 28.81 26.53 26.52 22.33

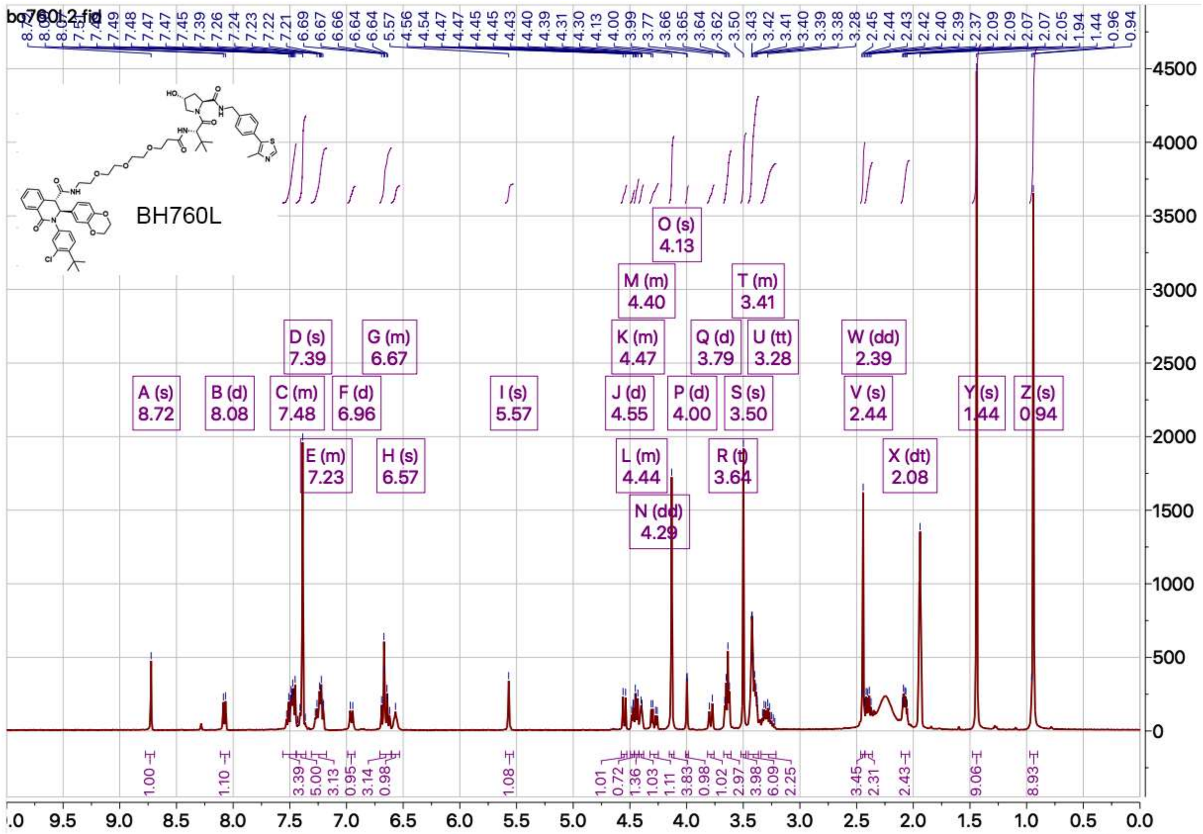
bo760L.2.fid
BH760L
A (s) 8.72
B (d) 8.08
C (m) 7.48
D (s) 7.39
E (m) 7.23
F (d) 6.96
G (m) 6.67
H (s) 6.57
I (s) 5.57
J (d) 4.55
K (m) 4.47
L (m) 4.44
M (m) 4.40
N (dd) 4.29
O (s) 4.13
P (d) 4.00
Q (d) 3.79
R (t) 3.64
S (s) 3.50
T (m) 3.41
U (tt) 3.28
V (s) 2.44
W (dd) 2.39
X (dt) 2.08
Y (s) 1.44
Z (s) 0.94

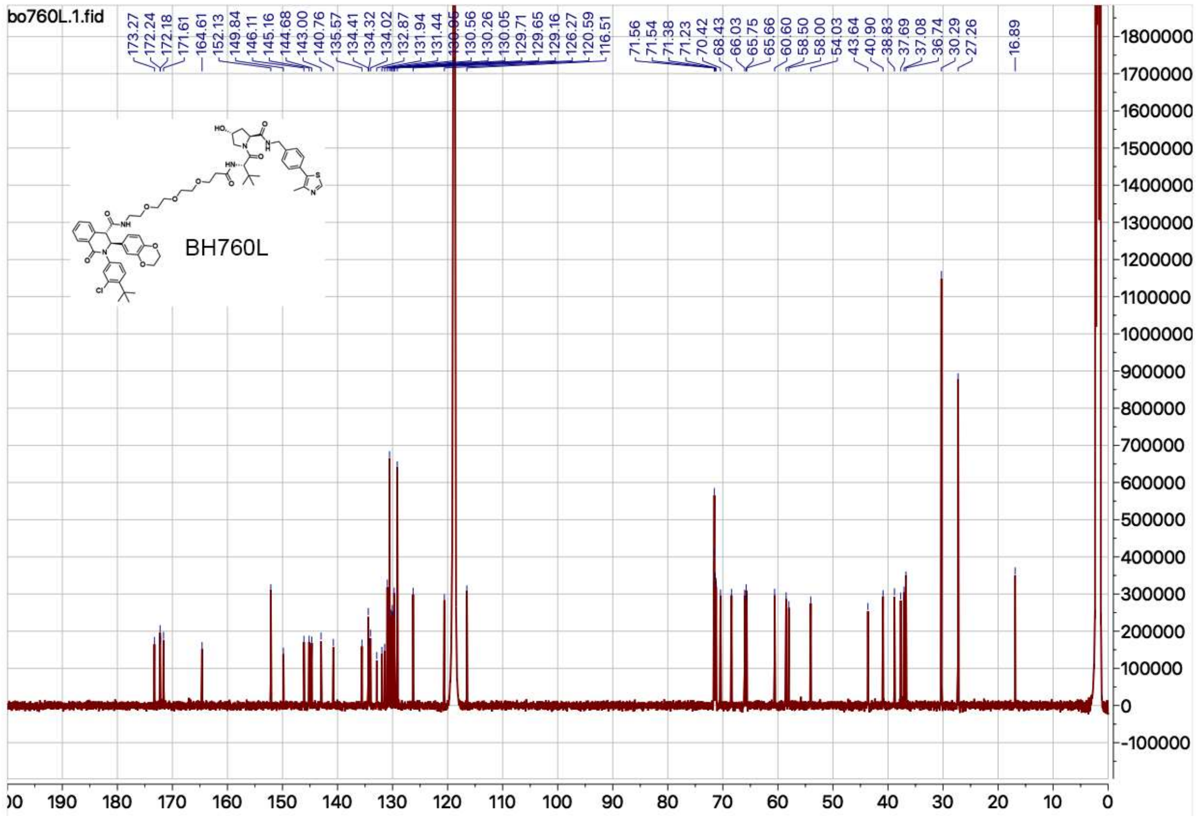
bo760L.1.fid
BH760L
173.27 172.24 172.18 171.61 164.61 152.13 149.84 146.11 145.16 144.68 143.00 140.76 135.57 134.41 134.32 134.02 132.87 131.94 131.44 130.56 130.26 130.05 129.71 129.65 129.16 126.27 120.59 116.51 71.56 71.54 71.38 71.23 70.42 68.43 66.03 65.75 65.66 60.60 58.50 58.00 54.03 43.64 40.90 38.83 37.69 37.08 36.74 30.29 27.26 16.89

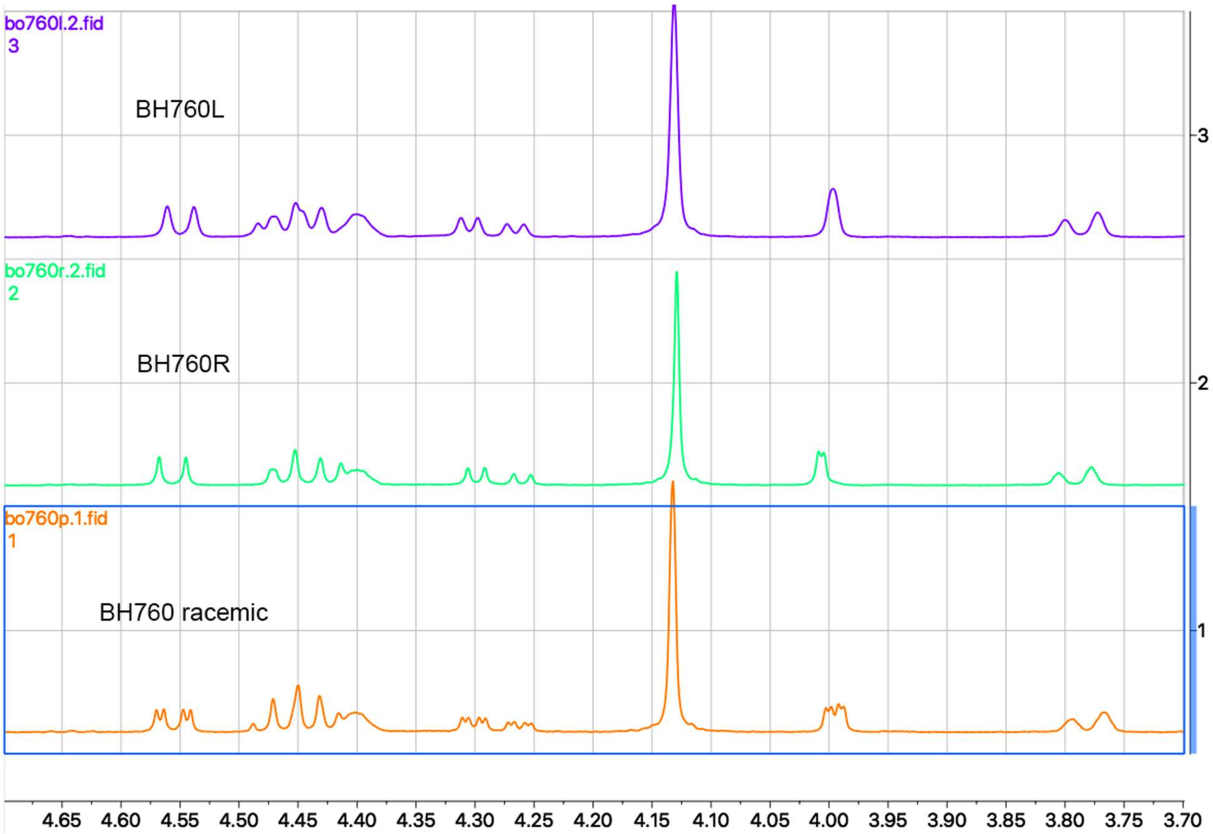
bo760l.2.fid
3
BH760L
bo760r.2.fid
2
BH760R
bo760p.1.fid
1
BH760 racemic
4.65 4.60 4.55 4.50 4.45 4.40 4.35 4.30 4.25 4.20 4.15 4.10 4.05 4.00 3.95 3.90 3.85 3.80 3.75 3.70

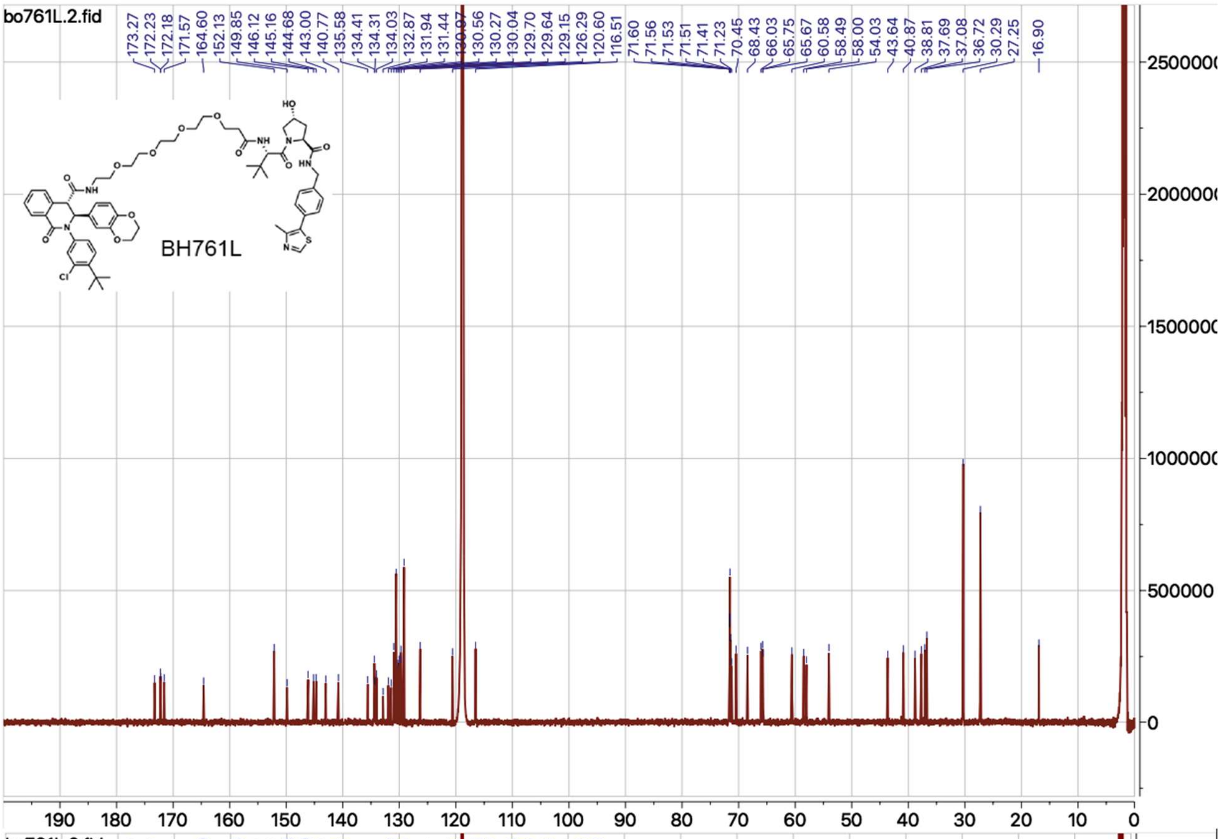
bo761L.2.fid
173.27
172.23
172.18
171.57
164.60
152.13
149.85
146.12
145.16
144.68
143.00
140.77
135.58
134.41
134.31
134.03
132.87
131.94
131.44
130.56
130.27
130.04
129.70
129.64
129.15
126.29
120.60
116.51
71.60
71.56
71.53
71.51
71.41
71.23
70.45
68.43
66.03
65.75
65.67
60.58
58.49
58.00
54.03
43.64
40.87
38.81
37.69
37.08
36.72
30.29
27.25
16.90
BH761L
190 180 170 160 150 140 130 120 110 100 90 80 70 60 50 40 30 20 10 0

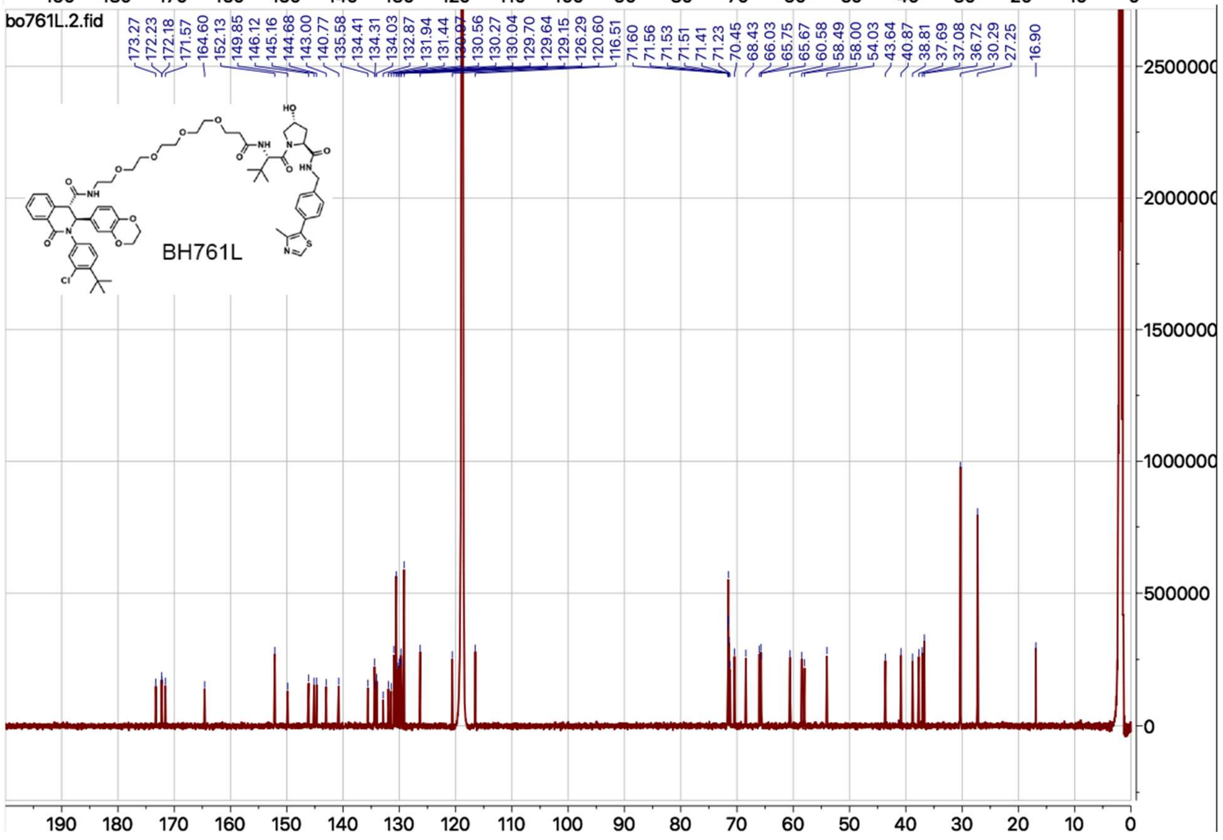
bo761L.2.fid
173.27
172.23
172.18
171.57
164.60
152.13
149.85
146.12
145.16
144.68
143.00
140.77
135.58
134.41
134.31
134.03
132.87
131.94
131.44
130.56
130.27
130.04
129.70
129.64
129.15
126.29
120.60
116.51
71.60
71.56
71.53
71.51
71.41
71.23
70.45
68.43
66.03
65.75
65.67
60.58
58.49
58.00
54.03
43.64
40.87
38.81
37.69
37.08
36.72
30.29
27.25
16.90
BH761L
190 180 170 160 150 140 130 120 110 100 90 80 70 60 50 40 30 20 10 0

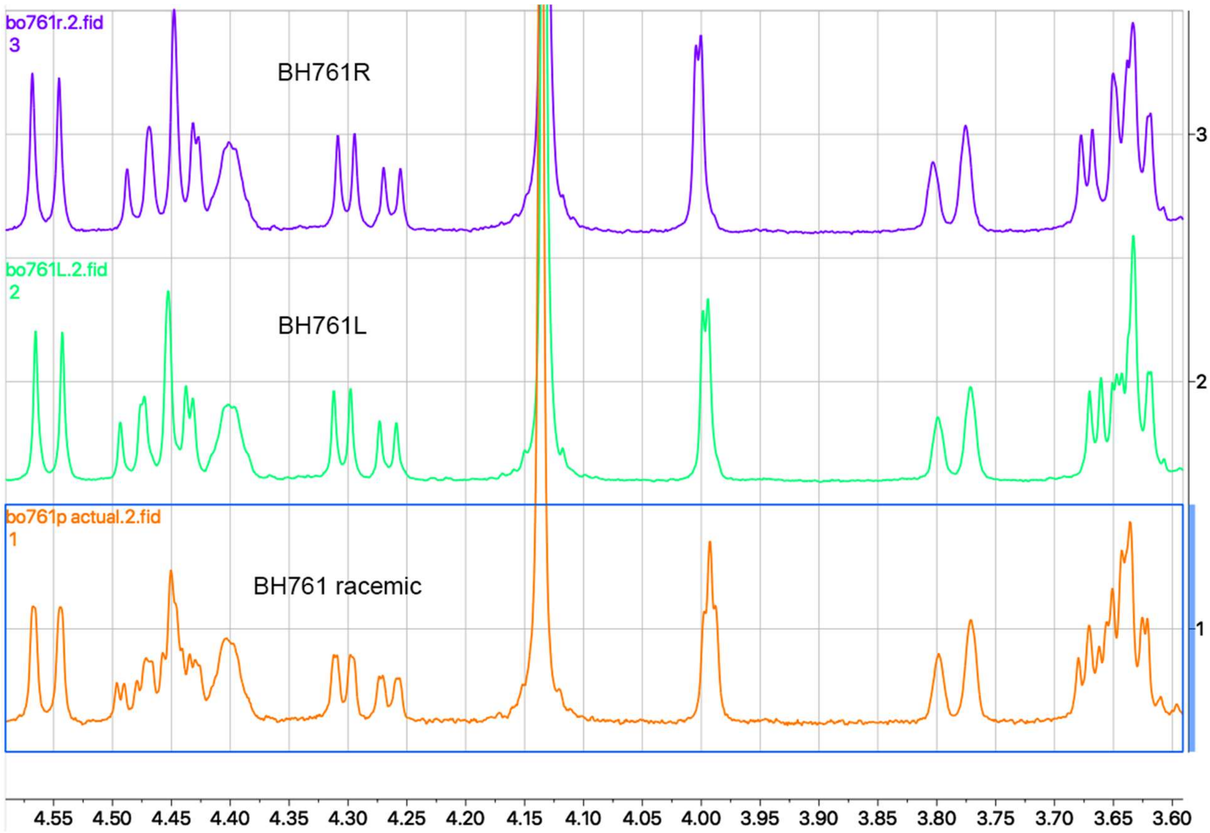
bo761r.2.fid
3
BH761R
bo761L.2.fid
2
BH761L
bo761p actual.2.fid
1
BH761 racemic
3
2
1
4.55
4.50
4.45
4.40
4.35
4.30
4.25
4.20
4.15
4.10
4.05
4.00
3.95
3.90
3.85
3.80
3.75
3.70
3.65
3.60

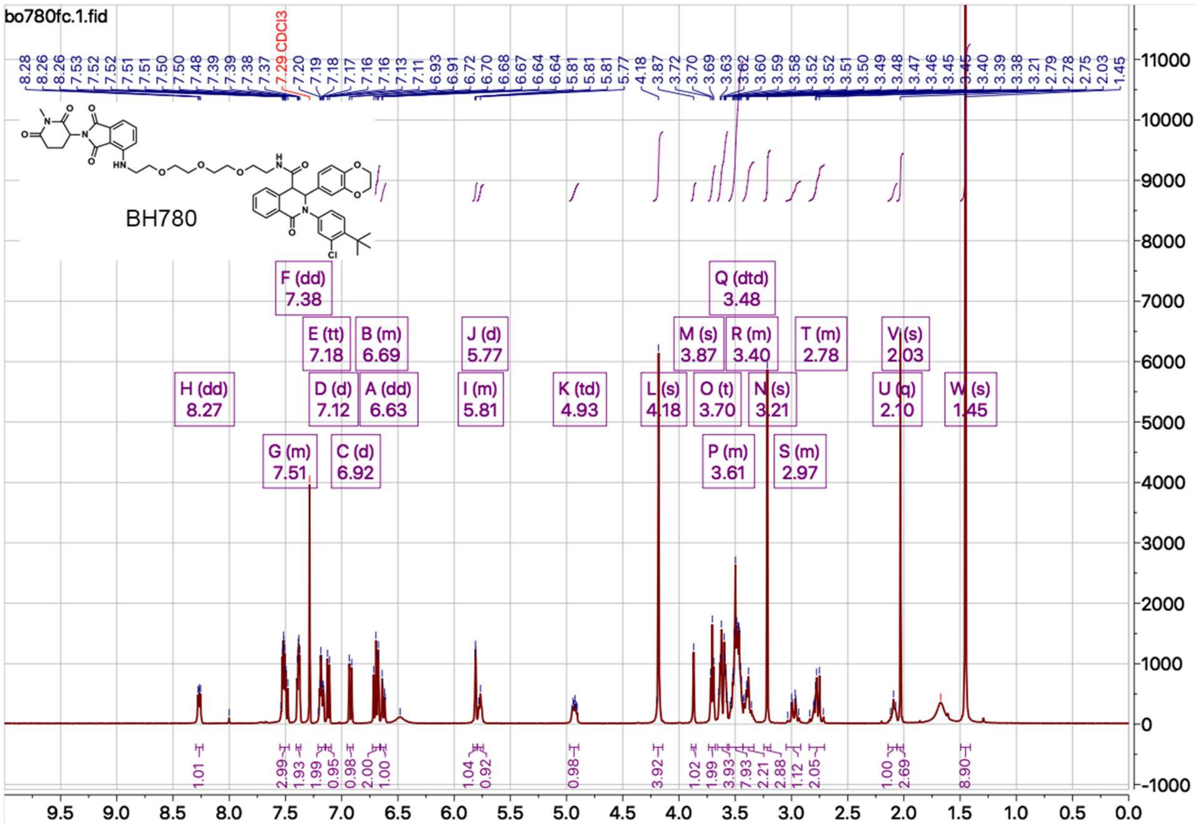
bo780fc.1.fid
BH780
7.29 CDCl3
H (dd) 8.27
G (m) 7.51
F (dd) 7.38
E (tt) 7.18
D (d) 7.12
C (d) 6.92
B (m) 6.69
A (dd) 6.63
J (d) 5.77
I (m) 5.81
K (td) 4.93
L (s) 4.18
M (s) 3.87
O (t) 3.70
P (m) 3.61
Q (dtd) 3.48
R (m) 3.40
N (s) 3.21
S (m) 2.97
T (m) 2.78
U (q) 2.10
V (s) 2.03
W (s) 1.45

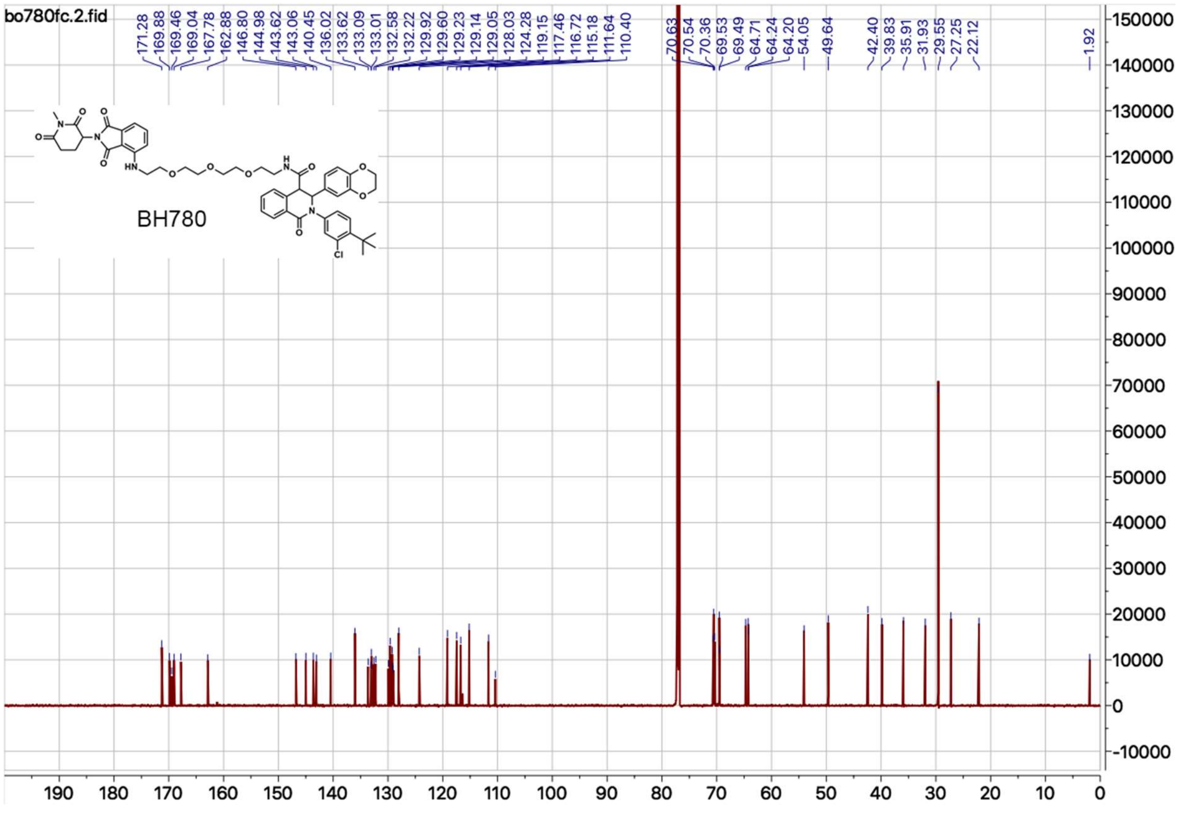
bo780fc.2.fid
BH780
171.28 169.88 169.46 169.04 167.78 162.88 146.80 144.98 143.62 143.06 140.45 136.02 133.62 133.09 133.01 132.58 132.22 129.92 129.60 129.23 129.14 129.05 128.03 124.28 119.15 117.46 116.72 115.18 111.64 110.40 70.63 70.54 70.36 69.53 69.49 64.71 64.24 64.20 54.05 49.64 42.40 39.83 35.91 31.93 29.55 27.25 22.12 1.92

Representative HPLC traces
Purified PROTAC compounds (10 mg) were separated by chiral chromatography using a Shimadzu HPLC with a Phenomenex Lux Cellulose-1 semi-prep column. The enantiomers / diastereomers were eluted with an isocratic gradient of 100% MeCN. The leftmost peak is designated L and the rightmost peak R.

BH690L/R

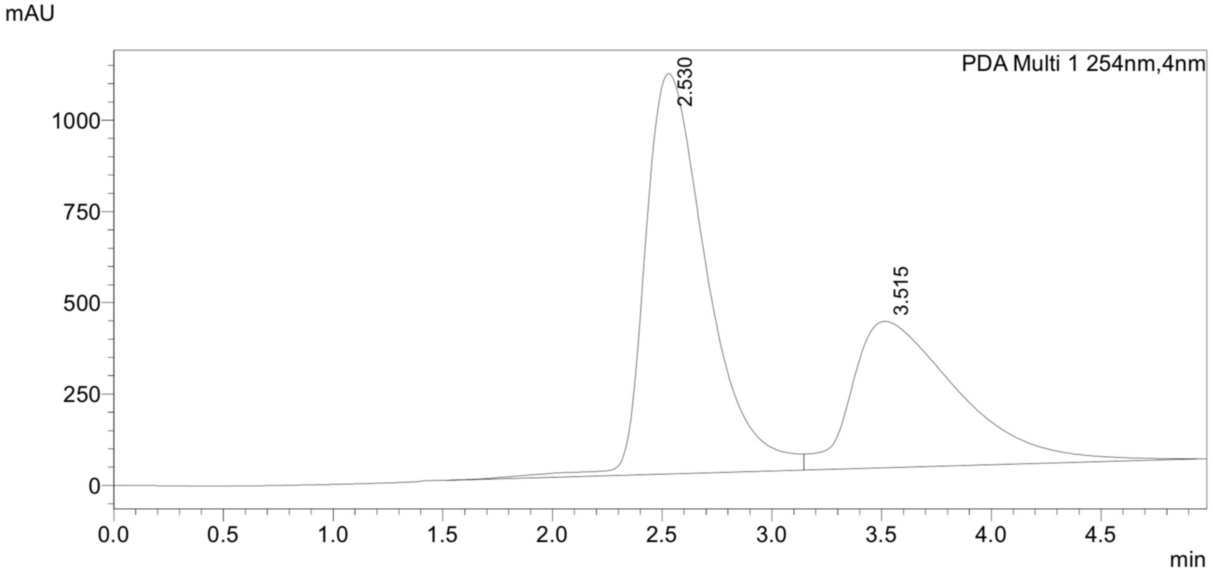

BH741L/R

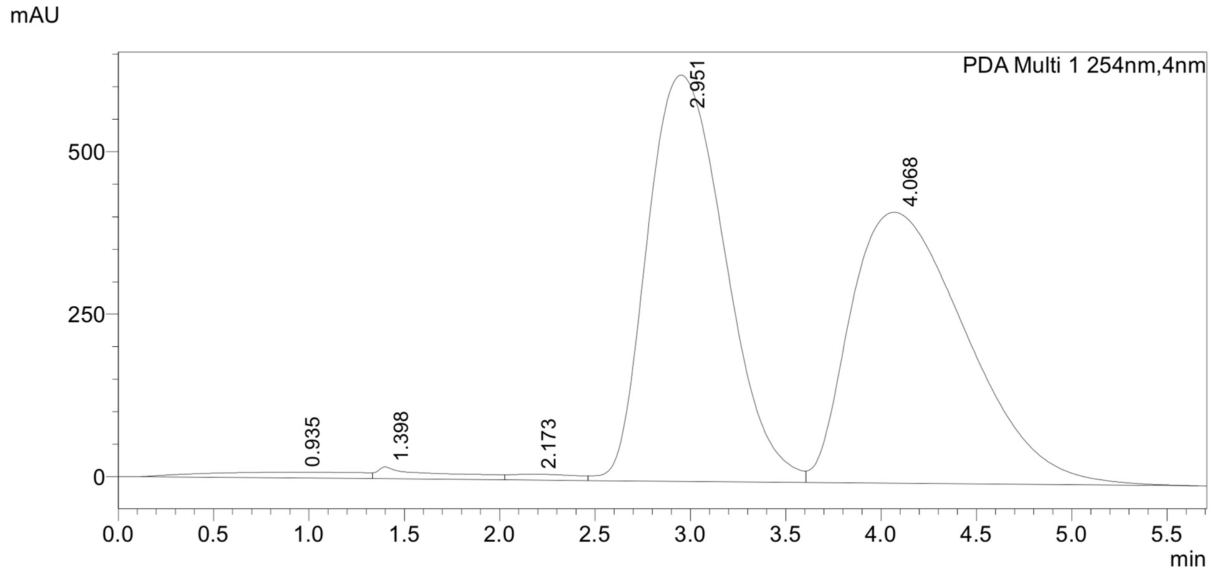

BH760L/R

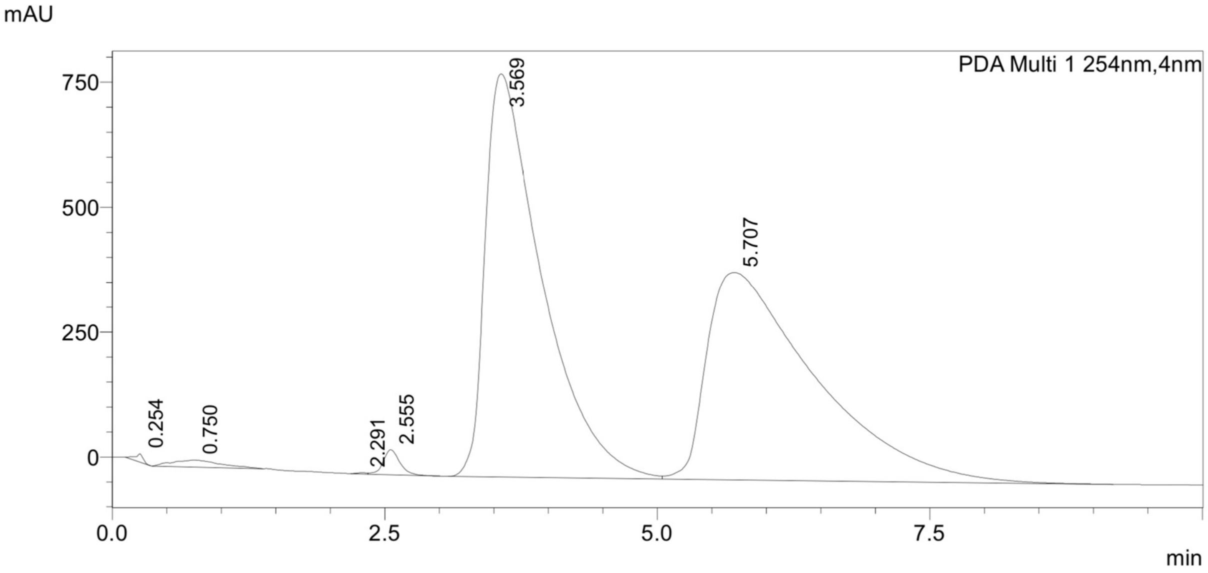

BH780L/R

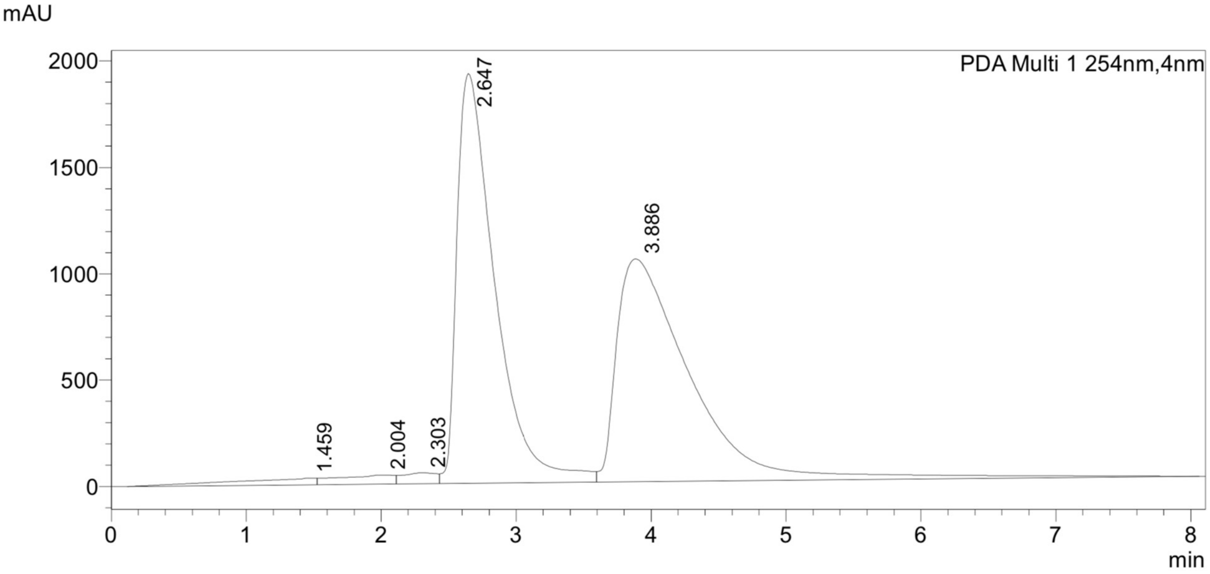

Supplement: Supplementary file 1 — Supplementary Material [file CMDC-21-e202500715-s001.pdf]
